# The transient-chelating-group-controlled stereoselective Rh(i)-catalyzed silylative aminocarbonylation of 2-alkynylanilines: access to (*Z*)-3-(silylmethylene)indolin-2-ones†

**DOI:** 10.1039/d2sc03009h

**Published:** 2022-07-27

**Authors:** Ya-Fei Han, Gui-Fen Lv, Yang Li, Li-Jun Wu, Xuan-Hui Ouyang, Jin-Heng Li

**Affiliations:** Key Laboratory of Jiangxi Province for Persistent Pollutants Control and Resources Recycle, Nanchang Hangkong University Nanchang 330063 China liyang8825490@126.com jhli@hnu.edu.cn; College of Sciences, Central South University of Forestry and Technology Changsha 410004 China lijunwu1105@hnu.edu.cn; Key Laboratory of Medicinal Chemistry for Natural Resource, Ministry of Education, Yunnan Provincial Center for Research & Development of Natural Products, School of Chemical Science and Technology, Yunnan University Kunming Yunnan 650091 China; State Key Laboratory of Applied Organic Chemistry, Lanzhou University Lanzhou 730000 China; School of Chemistry and Chemical Engineering, Henan Normal University Xinxiang Henan 453007 China

## Abstract

A new method involving mild acryl transient-chelating-group-controlled stereoselective Rh(i)-catalyzed silylative aminocarbonylation of 2-alkynylanilines with CO and silanes is presented for producing (*Z*)-3-(silylmethylene)indolin-2-ones. Upon using an acryl transient chelating group, 2-alkynylanilines undergo an unprecedented alkyne *cis*-silylrhodation followed by aminocarbonylation to assemble (*Z*)-3-(silylmethylene)indolin-2-ones. Mechanistic studies show that acryl transient chelating effects result in the key alkyne *cis*-silylrhodation process.

## Abstract 2

Transient-chelating-group-controlled stereoselective rhodium(I)-catalyzed silylative aminocarbonylation of 2- alkynylanilines with CO and silanes toward (Z)-3-(silylmethylene)indolin-2-ones is presented.

## Graphical abstract

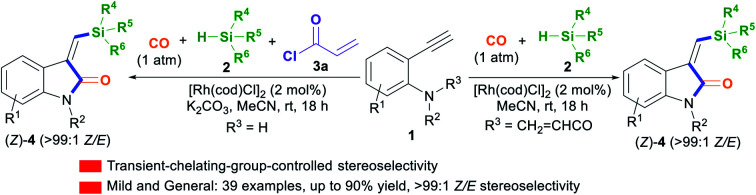

## Introduction

**Fig. 1:**
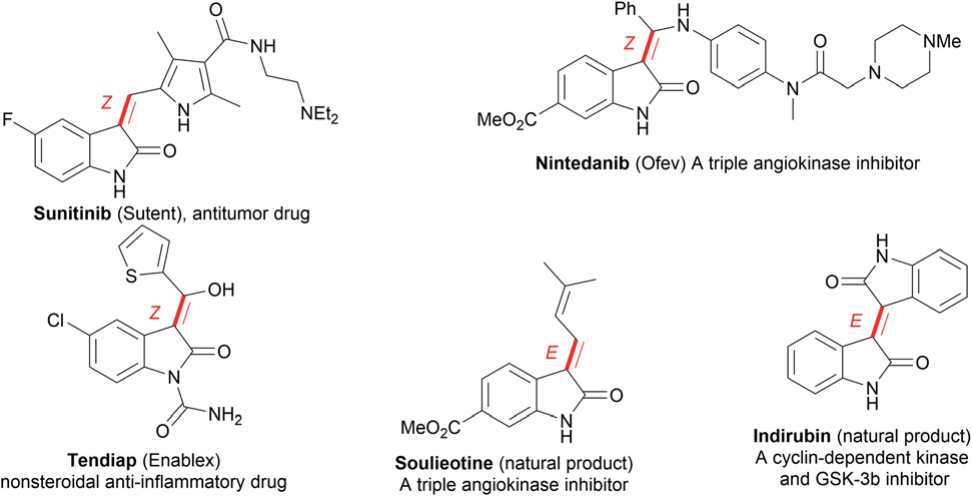
Selected examples of important 3-methylene-oxindoles.

**Scheme 1:**
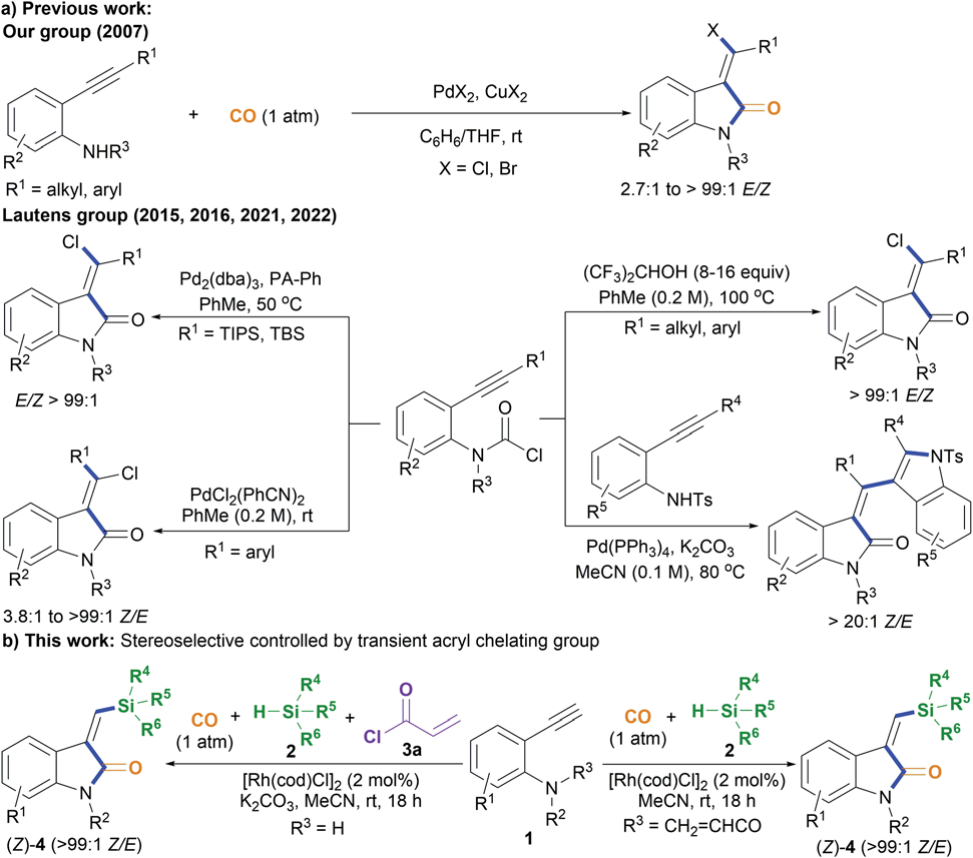
The stereoselective construction of functionalized 3-methylene-oxindoles.

Oxindoles, including methylene oxindoles, are a class of importantly coveted scaffolds for organic and medicinal chemistry purposes due to their omnipresence in natural products and biologically active molecules, and their widely established utilization as versatile synthetic building blocks.^1,2^ In particular, the use of 3-methylene-indolinone scaffolds has already been successfully established for VEGFR, Trk A, CDK, and GSK3 kinase inhibition, antitumor, antibacterial, anti-inflammatory, analgesic, and antimalarial applications (Fig. 1).^1^ As a result, developing efficient methods, especially stereoselective ones, for the synthesis of a diverse range of 3-methylene-indolinones is unarguably critical for continued progress in the area of drug development.^3–9^ Despite this growing demand, the stereoselective construction of the substituted methylene moiety of 3-methylene-oxindoles has been a longstanding challenge and, for these reasons, highly stereoselective preparation methods remain rare to date. Classical approaches for the assembly of methylene oxindoles mainly involve the intermolecular condensation of oxindoles with aryl carbonyl compounds, including diaryl ketones and aromatic formaldehydes, but these transformations face serious stereoselective control issues and substrate scope limitations.^1,3^ To overcome these issues, transition-metal-catalyzed tandem annulation reactions with unsaturated hydrocarbons,^4^ such as cross-coupling-enabled annulation cascades of *N*-(2-haloaryl)propiolamides,^5^*N*-arylpropiolamides,^6^ or 2-(alkynyl)arylisocyanates;^7^ the carbonylative annulation of 2-alkynylanilines or 2-alkenylanilines;^8^ the chloroacylation of alkyne-tethered carbamoyl chlorides;^9^ and the cross-dehydrogenation coupling (CDC) of 2,3-diarylacrylamides or *N*-cinnamoylanilines,^10^ have been developed. Common transition-metal catalysts (such as those containing Pd, Rh, Co_2_Rh_2_, and Ni) are efficient for use in these transformations to access various functionalized 3-methylene-oxindoles; however, the careful control of stereoselectivity sometimes remains a problem, with most configurations being unknown before the conclusion of the reaction. Moreover, reports detailing the deliberate control of stereoselectivity are dominated by the introduction of halogen atoms to construct 3-(halogenated methylene) scaffolds; as a result, there is an urgent need to discover conceptually novel stereoselectivity-control strategies for building diverse functionalized scaffolds other than halogenated ones. For example, our group has reported the palladium-catalyzed carbonylative annulation of 2-alkynylanilines with CO for producing 3-(halomethylene)-indolin-2-ones using stoichiometric CuX_2_ (X = Br, Cl) as both the halogen source and oxidant (Scheme 1a).^8*a*^ The stereoselectivity mainly depended on the substrate choice, and the assembly of (*E*)-3-(halomethylene)-indolin-2-ones is limited to 2-(alkylalkynyl)-anilines and sterically bulky 2-(3-substituted arylalkynyl)anilines. Lautens, Schoenebeck, and coworkers disclosed the Pd(0)-catalyzed *trans*-selective intramolecular chloroacylation of alkyne-tethered carbamoyl chlorides for assembling (*E*)-3-(halomethylene)indolin-2-ones, in which both sterically bulky silyl alkynyl substituents (such as TIPS and TBS) and bulky phosphorus ligands (such as phenyl phosphaadamantanes (PA-Ph)) are necessary to precisely direct the stereoselectivity toward (*E*)-isomers.^9*a*,*b*^ Very recently, Lautens and coworkers found that the use of hexafluoroisopropanol at high temperature (about 100 °C) allowed for the cycloisomerization of alkyne-tethered carbamoyl chlorides to forge only (*E*)-3-(chloromethylene)oxindoles.^9*d*^ This method has the advantage of simple operation and stereospecificity under metal-free conditions, but it is not applicable to sterically bulky TIPS alkynyl substituents. The same group developed a Pd(ii)-catalysis-based method to shift the stereoselectivity of the intramolecular chloroacylation of alkyne-tethered carbamoyl chlorides mainly toward the corresponding (*Z*)-isomers, with *Z*/*E* ratios ranging from 3.8 : 1 to >99 : 1.^9*c*^ Subsequently, they employed a similar Pd(ii) catalysis strategy to allow the domino cyclization of alkyne-tethered carbamoyl chlorides with 2-ethynylanilines through linked C(sp^2^)–C(sp^2^) bond stereospecific formation to access (*Z*)-3-((1*H*-indol-3-yl)methylene)indolin-2-ones.^9*e*^ By comparing these findings,^8,9^ steric hindrance effects and, especially, cooperative ligand/substrate coordination with transition-metal catalysts unarguably play important roles in the stereoselectivity control.

On that basis, we envisioned that if a transient chelating group^11^ was present to coordinate with transition-metal catalysts, it may be possible to carefully control the corresponding stereoselectivity. Herein, we report a new method involving the acryl-transient-chelating-group-controlled stereoselective [Rh^I^(cod)Cl]_2_-catalyzed silylative aminocarbonylation of 2-alkynylanilines with CO and silanes,^12^ enabling the synthesis of (*Z*)-3-(silylmethylene)indolin-2-ones in moderate to good yields and with >99 : 1 *Z*/*E* stereoselectivity (Scheme 1b). The method utilizes an *in situ* generated acryl group on the nitrogen atom as the transient chelating group to coordinate with the active Rh^I^ species, thus resulting in unprecedented alkyne *cis*-silylrhodation followed by aminocarbonylation, providing (*Z*)-3-(silylmethylene)indolin-2-ones.

## Results and discussion

We began to test our hypothesis that a transient chelating group could control the stereoselectivity during the silylative aminocarbonylation reaction with the use of *N*-(4-bromobenzyl)-2-ethynylaniline 1a, CO, triethylsilane 2a, and acryloyl chloride 3a as starting materials (Table 1). In the presence of 2 mol% [Rh^I^(cod)Cl]_2_, 2 equiv. of K_2_CO_3_, and 1 equiv. of chloride 3a, the silylative aminocarbonylation of substrate 1a with CO (1 atm) and triethylsilane 2a at room temperature after 18 h was efficiently performed, giving the desired (*Z*)-1-methyl-3-((triethylsilyl)methylene)indolin-2-one 4aa with 58% yield and >99 : 1 *Z*/*E* stereoselectivity (Table 1, entry 1). However, omitting the chloride 3a led to a lower yield (30%) and stereoselectivity inversion (1 : 5 *Z*/*E*) (Table 1, entry 2). Gratifyingly, the reaction could be efficiently executed to deliver 75% yield of 4aa in the absence of both K_2_CO_3_ and chloride 3a, but the stereoselectivity was shifted to 1 : 5 *Z*/*E* (Table 1, entry 3). Decreasing (0.5 equiv.) or increasing (1.5 equiv.) the amount of chloride 3a resulted in diminished yields (Table 1, entries 4 and 5). Both K_2_CO_3_ and [Rh^I^(cod)Cl]_2_ are essential for this reaction, since the omission of either resulted in no detectable desired product 4aa (Table 1, entries 6 and 11). A brief assessment of the effects of the loading of K_2_CO_3_ and the effects of the base (K_2_CO_3_, Na_2_CO_3_, Cs_2_CO_3_, NaHCO_3_, or Et_3_N) revealed that the reaction with 2 equiv. of K_2_CO_3_ afforded the best results (Table 1, entries 1 and 7–10). An evaluation of the Rh loading showed that 2 mol% [Rh^I^(cod)Cl]_2_ was the best option (Table 1, entry 1 *versus* entries 12 and 13). A series of Rh salts (Table 1, entries 14–17), including Rh^I^(cod)_2_BF_4_, Rh^II^(OAc)_2_, [Rh^II^(CH_3_(CH_2_)_6_CO_2_)_2_]_2_, and [{CP*Rh^III^Cl_2_}_2_], were examined; they displayed high catalytic activity but all were less effective than [Rh^I^(cod)Cl]_2_, partly due to the need for triethylsilane to reduce them and form active Rh^I^ species. The solvent (MeCN, CH_2_Cl_2_, or DMF) was found to affect the yield and stereoselectivity, and MeCN was shown to be the optimal medium (Table 1, entry 1 *versus* entries 18 and 19).

**Table 1:** Optimization of the reaction conditions^a^

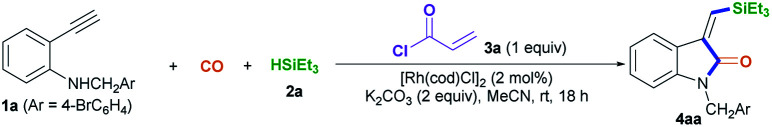		
Entry	Variation from the standard conditions	Yield^b^/%
1^b^	None	58 (>99 : 1)
2	Without 3a	30 (1 : 5)
3	Without 3a and K_2_CO_3_	75 (1 : 5)
4	3a (0.5 equiv.)	40 (>99 : 1)
5	3a (1.5 equiv.)	11 (>99 : 1)
6	Without base	Trace
7	K_2_CO_3_ (1.5 equiv.)	45 (>99 : 1)
8	K_2_CO_3_ (2.5 equiv.)	56 (>99 : 1)
9	Na_2_CO_3_ instead of K_2_CO_3_	41 (>99 : 1)
10	Cs_2_CO_3_, NaHCO_3_, or Et_3_N instead of K_2_CO_3_	Trace
11	Without [Rh(cod)Cl]_2_	0
12	[Rh(cod)Cl]_2_ (1 mol%)	53
13	[Rh(cod)Cl]_2_ (5 mol%)	60
14	Rh(cod)_2_BF_4_ instead of [Rh(cod)Cl]_2_	38 (>99 : 1)
15	Rh(OAc)_2_ instead of [Rh(cod)Cl]_2_	25 (>99 : 1)
16	[Rh(CH_3_(CH_2_)_6_CO_2_)_2_]_2_ instead of [Rh(cod)Cl]_2_	20 (>99 : 1)
17	[{CP*RhCl_2_}_2_] (2) instead of [Rh(cod)Cl]_2_	51 (>99 : 1)
18	CH_2_Cl_2_ instead of MeCN	55 (>99 : 1)
19	DMF instead of MeCN	34 (15 : 1)

a Standard conditions: 1a (0.2 mmol), 2a (0.2 mmol), 3a (0.2 mmol), [Rh(cod)Cl]_2_ (2 mol%), K_2_CO_3_ (0.4 mmol; 2 equiv.), and MeCN (2 mL), in argon, at room temperature, for 18 h.

b Isolated yield. The *Z*/*E* value is given in parentheses, determined based on GC-MS analysis of the crude product.

**Scheme 2:**
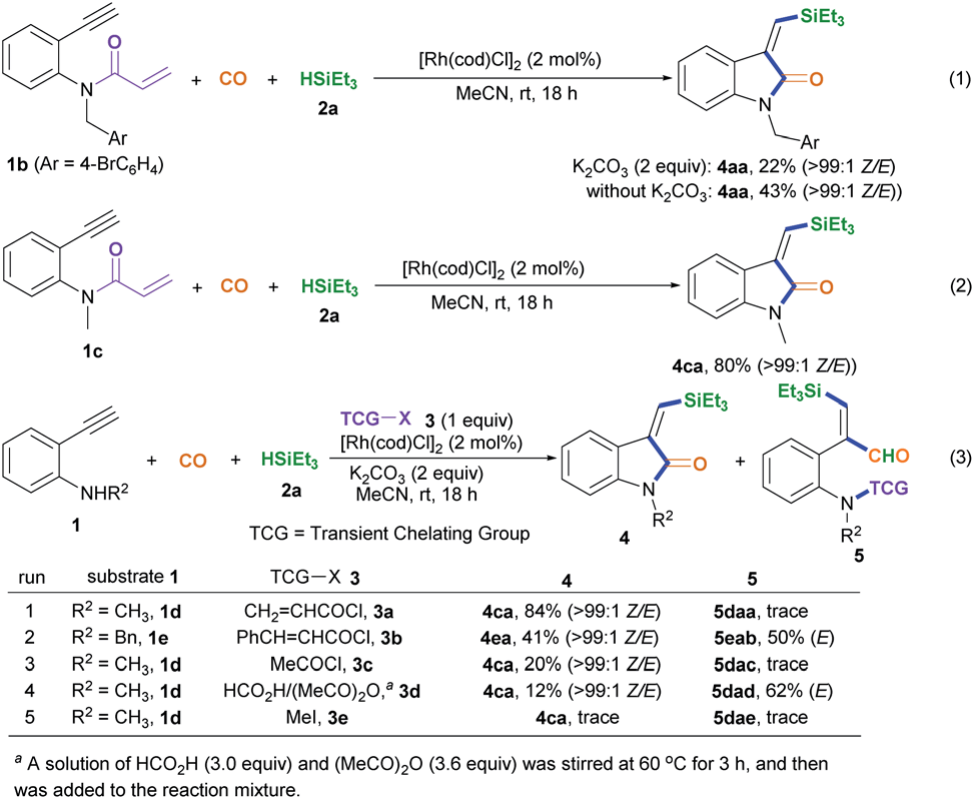
Screening the transient chelating groups (3).

With the optimized conditions in hand, we set out to further investigate the feasibility of this transient-chelating-group-based strategy (Scheme 2). Directly using *N*-(4-bromobenzyl)-*N*-(2-ethynylphenyl)acrylamide 1b in a reaction with CO, silane 2a, [Rh(cod)Cl]_2_, and K_2_CO_3_ afforded (*Z*)-4aa with a lower yield (22%) (Scheme 2, eqn (1)), whereas the omission of K_2_CO_3_ increased the yield of (*Z*)-4aa to 43%. The results show that the base can improve the acylation process *via* the removal of chloride ions, but it suppresses the silylative aminocarbonylation. Furthermore, the in-situ-generated transient chelating group process is more efficient than the process involving the direct use of substrate 1b, probably because coordination effects relating to the acryloyl chloride may improve the catalytic activity of the Rh catalyst. Similarly, the treatment of *N*-(2-ethynylphenyl)-*N*-methylacrylamide 1c with CO, silane 2a, and [Rh(cod)Cl]_2_ also afforded the (*Z*)-isomer 4ca in 80% yield (Scheme 2, eqn (2)); meanwhile acryloyl chloride was found to be the optimal transient-chelating-group reagent and it could efficiently allow the silylative aminocarbonylation of substrate 1d, stereoselectively assembling (*Z*)-4ca exclusively with a slightly increased yield (84%; Scheme 2, eqn (3), run 1). Using cinnamoyl chloride 3b in a reaction with the *N*-benzyl-substituted substrate 1e decreased the yield of (*Z*)-4ea to 41%, with 50% yield of the alkyne silylformylation product (*E*)-5eab (Scheme 2, eqn (3), run 2). Both acetyl chloride 3c and the formyl system 3d^13^ were less reactive (Scheme 2, eqn (3), runs 3 and 4). Notably, the treatment of the formyl system 3d with substrate 1d, CO, and silane 2a mainly resulted in the alkyne silylformylation product (*E*)-5dad in 62% yield, with a lower yield (12%) of (*Z*)-4ca (Scheme 2, eqn (3), run 4). These findings suggest that the *cis*-silyl vinyl-Rh intermediate may be initially formed *via* the *cis*-silylrhodation of the alkyne moiety, followed by the insertion of CO. However, methyl iodide 3e was inert (Scheme 2, eqn (3), run 5).

After establishing the optimal acryl transient chelating group, we next investigated the scope of this Rh-catalyzed stereoselective silylative aminocarbonylation protocol with respect to 2-ethynyl-*N*-acrylanilines 1 and silanes 2 for the synthesis of (*Z*)-3-(silylmethylene)indolin-2-ones 4 (Table 2). Various substituents, including benzyl (1e), 4-methoxybenzyl (1f), 4-(trifluoromethyl)benzyl group (1g), cyclopropylmethyl (1h), and allyl (1i) groups, on the nitrogen atom were well tolerated under the optimized conditions, affording the corresponding (*Z*)-isomers 4ea–ia in moderate to good yields. The substitution effects of the aniline moiety were evaluated (4ja–na), and the results showed that electronic effects and steric hindrance had no obvious influence on the reaction. 2-Alkynylanilines 1j–l, bearing a 5-Me, 5-F, or 5-Cl group on the aryl ring, could be stereoselectively converted to (*Z*)-4ja–la, respectively, with yields of 67–82%. Most importantly, the halogen atom, such as F, Cl, and Br, remains intact, so it can serve as a functional handle for further derivatization (4aa, 4ka–la). 2-Alkynylanilines 1m–n possessing an electron-donating Me group or an electron-withdrawing CN group at the 4-position were viable for obtaining (*Z*)-4ma–na with good yields. A variety of silanes, including trihexylsilane 2b, triisopropylsilane 2c, *tert*-butyldimethylsilane 2d, allyldimethyl-silane 2e, dimethyl(phenyl)silane 2f, methyldiphenylsilane 2g, and triphenylsilane 2h, tolerated the stereoselective silylative aminocarbonylation protocol, attaining (*Z*)-isomers 4db–dh with high yields.

**Table 2:** The reaction scope in terms of the 2-alkynylaniline (1) and silane (2)^a^

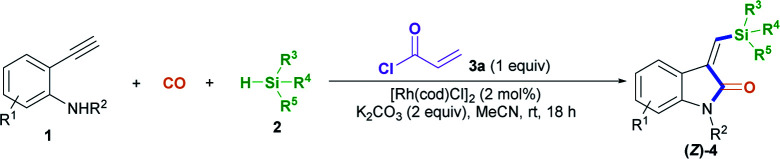
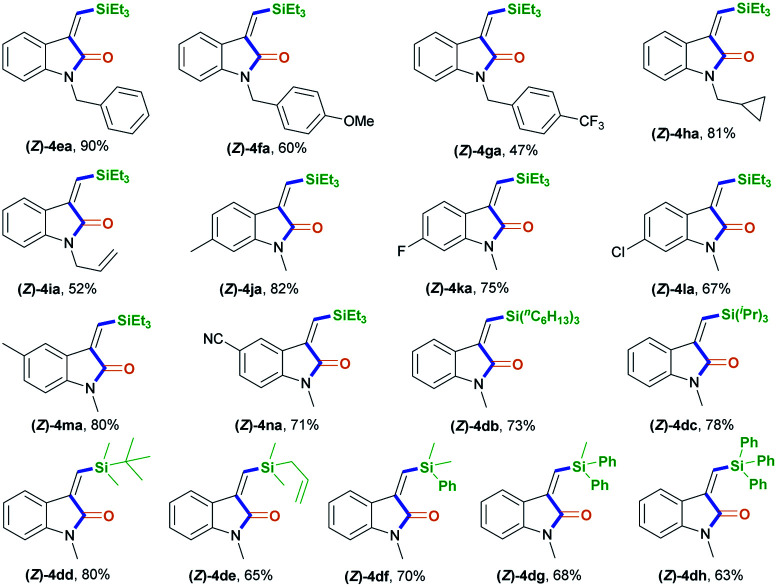

a Reaction conditions: 1 (0.2 mmol), 2 (0.2 mmol), 3a (0.2 mmol), [Rh(cod)Cl]_2_ (2 mol%), K_2_CO_3_ (0.4 mmol; 2 equiv.), and MeCN (2 mL), in argon, at room temperature, and for 18 h. Some side-products, such as the alkyne silylformylation product 5 and C–N decomposition products, were observed.

To demonstrate the generality of this silylative aminocarbonylation protocol, we directly used 2-ethynyl-*N*-acrylanilines 1 to execute silylative aminocarbonylation with CO and silanes 2 (Table 3). In the presence of [Rh(cod)Cl]_2_, CO, and silanes 2, *N*-acrylanilines 1o–r possessing substituents such as benzyl (1o), 4-methoxybenzyl (1p), 4-(trifluoromethyl)benzyl (1q), cyclopropylmethyl (1h), and allyl (1r) groups on the nitrogen atom could be successfully converted to 4ea–ia with moderate to good yields and >99 : 1 *Z*/*E* stereoselectivity. The substitution effects on the aromatic ring of the aniline moiety were investigated, and electron-donating substituents (such as Me; 4ja and 4ma) are more efficient than electron-withdrawing ones (such as F, Cl, and CN; 4ka–la and 4na). For example, the *N*-acrylanilines 1s and 1v possessing an electron-donating Me group at the 4- or 5-position, donating to the aniline moiety efficiently, underwent the reaction to afford 4ja and 4ma, respectively, with yields of 76% and 81%, whereas substrate 1w, having an electron-withdrawing CN group, delivered 4na with diminished yield (67%). The array of silanes 2b–h displayed high reactivity when reacting with substrate 1c, giving 3-(silylmethylene)indolin-2-ones 4db–dh with good yields.

**Table 3:** The silylative aminocarbonylation of *N*-(2-ethynylaryl) acrylamide (1) with CO and silanes (2)^a^

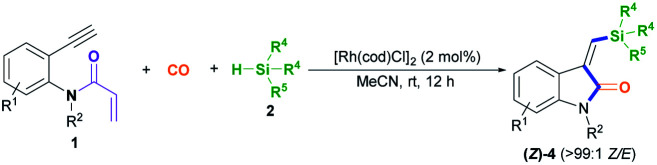
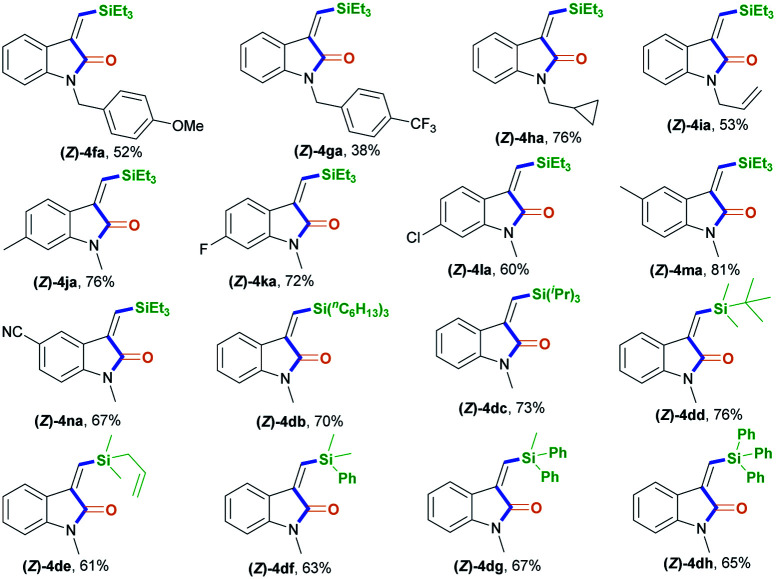

a Standard conditions: 1 (0.2 mmol), 2 (0.2 mmol), CO (1 atm), [Rh(cod)Cl]_2_ (2 mol%), and MeCN (2 mL), at room temperature and for 18 h. Some side-products, such as the alkyne silylformylation product 5 and C–N decomposition products, were observed.

**Scheme 3:**
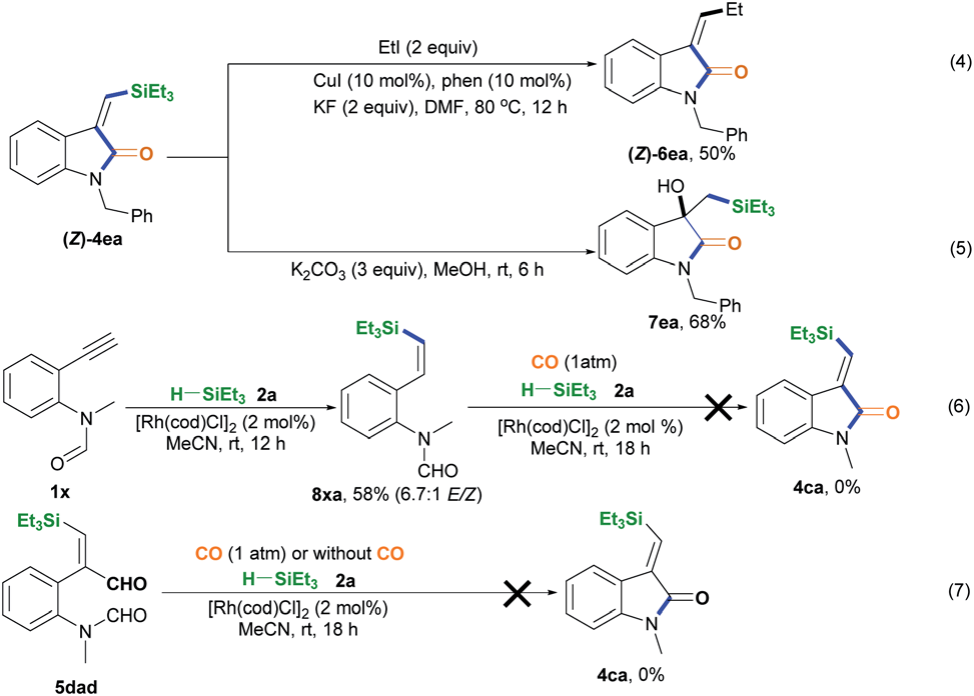
The utilization of (*Z*)-4ea and control experiments.

The synthetic utilization of the Si-containing product (*Z*)-4ea was conducted (Scheme 3).^14^ Hiyama coupling of (*Z*)-4ea with ethyl iodide, CuI, 1,10-phenanthroline (phen), and KF in DMF at 80 °C for 12 h was performed, successfully affording (*Z*)-4ea with 50% yield (Scheme 3, eqn (4)).^14*a*^ Using K_2_CO_3_, (*Z*)-4ea was converted into 1-benzyl-3-hydroxy-3-((triethylsilyl)methyl)indolin-2-one 7ea with 68% yield (Scheme 3, eqn (5)).^1^ Some control experiments were performed to understand the mechanism of this silylative aminocarbonylation protocol. Without CO, 2-alkynylaniline 1× underwent the alkyne hydrosilylation reaction with silane 2a to afford 8xa in 58% yield and with 6.7 : 1 *E*/*Z* stereoselectivity (Scheme 3, eqn (6)). However, substrate 8xa is inert toward the carbonylation reaction in the presence of CO, silane 2a, and [Rh(cod)Cl]_2_ (Scheme 3, eqn (6)). These observations suggest that the Si–Rh complex intermediate is initially formed and then addition across the C–C bond generates the silyl vinyl-Rh intermediate, followed by reductive elimination and protonation, ruling out the generation of the alkynehydrosilylation intermediate 8 during this silylative aminocarbonylation process. We found that substrate 5dad treated with silane 2a and [Rh(cod)Cl]_2_ in the presence or absence of CO could not afford the desired product 4ca (Scheme 3, eqn (7)), supporting the idea that the silylative aminocarbonylation reaction does not involve the formation of intermediate 5.

**Scheme 4:**
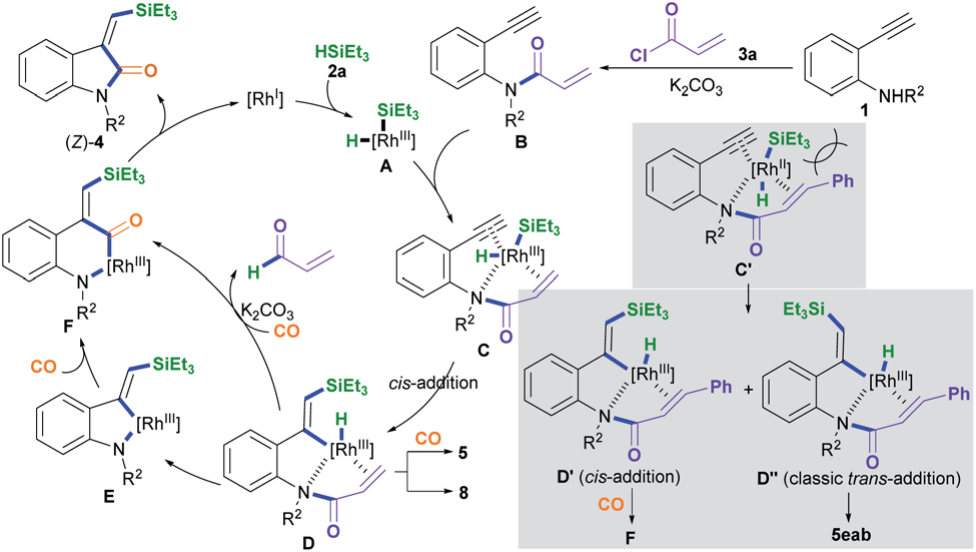
A possible reaction mechanism.

A plausible mechanism for the silylative aminocarbonylation protocol was proposed (Scheme 4).^5–12^ Oxidative addition of the active Rh^I^ species to silane 2a forms the H–Rh^III^–Si complex intermediate A.^12^ Subsequently, coordination of the Rh^III^ complex intermediate A with the *N*-(2-ethynylphenyl)acrylamide intermediate B, which is *in situ* generated from the reaction of 2-alkynylaniline 1 with acryloyl chloride and K_2_CO_3_, affords the intermediate C. Therein, intermediate C containing an acryl transient chelating group can strongly coordinate with the Rh^III^ species, thus resulting in *cis*-silylrhodation across the C≡C bond to form the *cis*-silyl vinyl-Rh^III^ intermediate D.^11,12^ Intermediate D may undergo two pathways for the insertion of CO:^5*c*–*f*,8,12^ One is the direct insertion of CO into the vinyl–Rh bond with the simultaneous formation of a N–Rh bond *via* the reductive loss of the acryl group with the aid of the base (K_2_CO_3_)^12*j*,*k*^ to generate the carbonyl-Rh^III^–N six-membered ring intermediate F; the other involves the formation of the vinyl-Rh^III^–N five-membered ring intermediate E through the reductive decomposition of the acryl C(sp^2^)–N bond with the aid of the base,^12*j*,*k*^ followed by the insertion of CO to generate the intermediate F. The reductive elimination of intermediate F results in the desired product (*Z*)-4 and regenerates the active Rh^I^ species.

Using cinnamoyl chloride 3b as the transient chelating group may afford the alkyne *cis*-addition intermediate D′ and the alkyne *trans*-addition intermediate D′′ due to steric hindrance and electron effects from the cinnamoyl group.^11,12^ The alkyne *cis*-addition intermediate D′ undergoes CO insertion, C–N bond cleavage, and N–Rh bond formation to afford the intermediate F, whereas the alkyne classic *trans*-addition intermediate D′′ may undergo hydroformylation with CO to form (*E*)-5eab. This is because the *in situ* generated cinnamyl C–N bond in the intermediate D′′ involving conjugative effects is more stable than the acryl C–N bond, leading to no cleavage of the cinnamyl C–N bond.

## Conclusions

In summary, we have developed a novel strategy involving a mild acryl transient chelating group for the stereoselective rhodium(i)-catalyzed silylative aminocarbonylation of 2-alkynylanilines with CO and silanes, enabling the formation of (*Z*)-3-(silylmethylene)indolin-2-ones. The method involves the use of an acryl transient chelating group to enable the unprecedented *cis*-silylrhodation of alkynes and aminocarbonylation cascades to produce (*Z*)-3-(silylmethylene)indolin-2-ones; the highlights include exquisite stereoselectivity, a wide substrate scope, and excellent functional group tolerance. This acryl-transient-chelating-group-controlled stereoselectivity strategy provides a conceptually novel approach for stereoselective transformations of unsaturated hydrocarbons and it could inspire the further development of new and efficient methods for stereoselective synthesis.

## Data availability

Experimental data is provided in the ESI.†

## Author contributions

L.-J. W. and J.-H. L. conceived and designed the experiments. Y.-F. H. and L.-J. W. carried out most of the experiments. Y.-F. H., Y. L., X.-H. O., L.-J. W. and J.-H. L. analysed the data. Y.-F. H., L.-J. W. and J.-H. L. prepared the manuscript. Y. L., L.-J. W. and J.-H. L. directed the project.

## Conflicts of interest

The authors declare no competing financial interests.

## Supplementary Material

SC-013-D2SC03009H-s001
