# Supplementary material for: The transient-chelating-group-controlled stereoselective Rh(i)-catalyzed silylative aminocarbonylation of 2-alkynylanilines: access to (Z)-3-(silylmethylene)indolin-2-ones
Source: Chem Sci. 2022 Jul 27;13(32):9425–31. doi: 10.1039/d2sc03009h (PMC9383873; doi:10.1039/d2sc03009h)
Supplement: SC-013-D2SC03009H-s001 [file SC-013-D2SC03009H-s001.pdf]

Electronic Supplementary Material (ESI) for Chemical Science.
This journal is © The Royal Society of Chemistry 2022

Supplementary Information

Transient Chelating Group-Controlled Stereoselective Rh(I)-Catalyzed Silylative Aminocarbonylation of 2-Alkynylanilines: Entry to (*Z*)-3-(Silylmethylene)indolin-2-ones

Ya-Fei Han,[a] Gui-Feng Lv,[a] Yang Li,*[a] Li-Jun Wu,*[b] Xuan-Hui Ouyang,[a] and Jin-Heng Li*[acde]

[a] *Key Laboratory of Jiangxi Province for Persistent Pollutants Control and Resources Recycle, Nanchang Hangkong University, Nanchang 330063, China. E-mail: liyang8825490@126.com, and jhli@hnu.edu.cn*

[b] *College of Sciences, Central South University of Forestry and Technology, Changsha, 410004, China. E-mail: lijunwu1105@hnu.edu.cn,*

[c] *Key Laboratory of Medicinal Chemistry for Natural Resource, Ministry of Education; Yunnan Provincial Center for Research & Development of Natural Products; School of Chemical Science and Technology, Yunnan University, Kunming, Yunnan 650091, China*

[d] *State Key Laboratory of Applied Organic Chemistry, Lanzhou University, Lanzhou 730000, China.*

[e] *School of Chemistry and Chemical Engineering, Henan Normal University, Xinxiang, Henan 453007, China*

List of Contents

(A) Typical experimental procedure

(B) Analytical data

(C) Spectra

(D) References

(A) Typical Experimental Procedure

(a) General Information

^{1}H NMR, ^{13}C NMR and ^{19}F NMR spectra were recorded on a Bruker 400 or 500 MHz advance spectrometer at room temperature in $CDCl_3$ using TMS as internal standard. Low-resolution mass spectra (LRMS) data were measured on GCMS-QP2010 Ultra or LC-MS: HPLC (Dionex Ultimate 3000) and MS (Thermo Scientific ISQ EC). High-resolution mass spectra (HRMS) was recorded on an electrospray ionization (ESI) apparatus using time-of-flight (TOF) mass spectrometry. Melting Points were recorded on Hanon MP100 Apparatus. Unless otherwise noted, all reactions were carried out using standard Schlenk techniques, and all starting materials and solvents were commercially available and were used without further purification. Column chromatography was performed on silica gel (300-400 mesh) using petroleum ether (PE)/ethyl acetate (EA).

(b) General Procedure for the Synthesis 2-Ethynylanilines (1).[1]

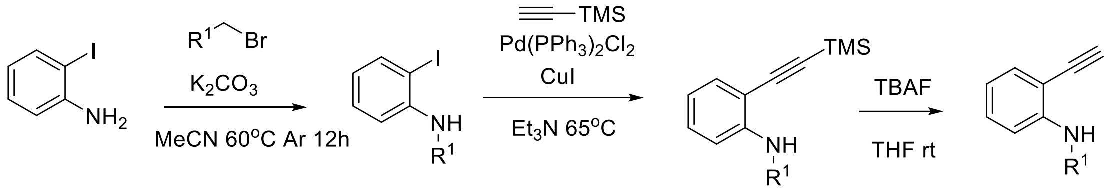

To a solution of o-iodoaniline (1 equiv), K_2CO_3 (1.5 equiv) in MeCN (10 mL) was added Bromide (1.2 equiv), the reaction mixture was heated to reflux for 12 h under Ar atmosphere. After the completion of the reaction as shown by TLC, water (10 mL) was used to dilute the reaction. The organic layer was extracted with ethyl acetate (10 mL × 3). The combined organic layer was washed with saturated brine, dried with Na_2SO_4. The crude product was purified by flash chromatography with PE/EA as elute.

Under a nitrogen atmosphere, to a triethylamine solution (3 equiv) of $Pd(PPh_3)_2Cl_2$ (2 mol%) and CuI (1 mol%) was added o-iodoaniline derivatives (1 equiv) and stirred for 10 mins, then added trimethylsilylacetylene (2 equiv) dropwise over 30 mins. The resulting suspension was allowed to be stirred for 4 hours at 65 ^{o}C. After completion of the reaction, the mixture was filtered through a short celite bed and concentrated

under reduced pressure. The crude product was purified by flash chromatography with PE/EA as elute.

Under a nitrogen atmosphere, TBAF (2 equiv) was added. The reaction mixture was stirred at rt for 30 min. Then the mixture was concentrated in vacuo. The residue was extracted with DCM (10 mL), and the combined organic layers were washed with brine (20 mL), dried over Na_2SO_4, filtered and evaporated in vacuo. The crude product was purified by flash chromatography with PE/EA as elute to afford 2-ethynylanilines **1**.

(c) Typical Experimental Procedure for Transient Chelating Group-Controlled Stereoselective Rh(I)-Catalyzed Silylative Aminocarbonylation of 2-Alkynylanilines (1) with CO and Silanes (2):

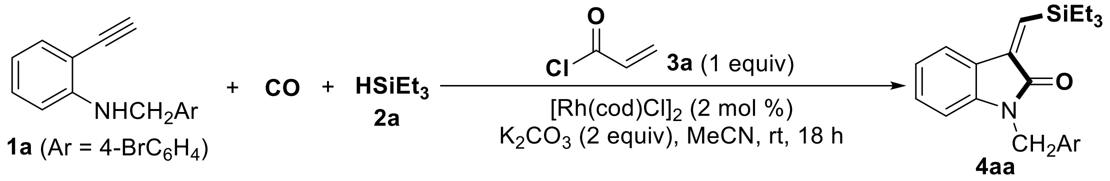

To a Schlenk tube were added 2-alkynylaniline **1a** (0.2 mmol), silane **2a** (0.2 mol), acryloyl chloride **3a** (0.2mmol), $[Rh(cod)Cl]_2$ (2 mol%), K_2CO_3 (0.4mmol) and CH_3CN (2 mL).Then the tube was charged with CO, and was stirred under room temperature for 18 h until complete consumption of starting material as monitored by TLC and/or GC-MS analysis. After the reaction was finished, the concentrated in vacuum, and the resulting residue was purified by silica gel column chromatography (hexane/ethyl acetate) to afford the desired products (*Z*)-**4**.

(B) Analytical data

(*Z*)-1-(4-bromobenzyl)-3-((triethylsilyl)methylene)indolin-2-one (4aa):

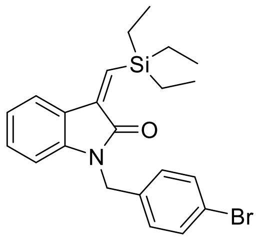

49.5 mg, 58% yield; Colorless oil; R_f = 0.2 (PE/EA = 20 : 1); ^{1}H NMR (500 MHz, $CDCl_3$) δ (ppm) 7.45 (t, *J* = 10.0 Hz, 3H), 7.16 (d, *J* = 7.0 Hz, 3H), 7.49 – 7.40 (m, 2H), 6.61 (d, *J* = 7.0 Hz, 1H), 4.88 (s, 2H), 1.00 (t, *J* = 7.5 Hz, 9H), 0.90 (q, *J* = 8.5 Hz, 6H); ^{13}C NMR (125 MHz, $CDCl_3$) δ (ppm) 167.1, 142.4, 141.8, 139.3, 135.2, 131.9, 129.7, 129.0, 123.3, 122.2, 121.4, 120.3, 108.8, 77.3, 77.1, 76.8, 42.9, 7.8, 3.7; LRMS (EI, 70 eV) m/z (%): 429 (M^++2, 1), 427 (M^+, 1), 398 (99), 400 (100), 171 (87), 169 (89); HRMS *m/z* (ESI) calcd for $C_{22}H_{27}BrNOSi$ ($[M+H]^+$) 428.1040, found 428.1058.

(***Z***)**-1-methyl-3-((triethylsilyl)methylene)indolin-2-one (4ca)：**

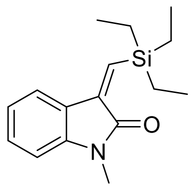

45.9 mg, 84% yield; Colorless oil; R_f = 0.2 (PE/EA = 20 : 1); ^{1}H NMR (400 MHz, $CDCl_3$) δ (ppm) 7.44 (d, *J* = 7.2 Hz, 1H), 7.28 (t, *J* = 7.6 Hz, 1H), 7.02 (d, *J* = 7.2 Hz, 1H), 6.94 (s, 1H), 6.78 (t, *J* = 7.6 Hz, 1H), 3.23 (s, 3H), 0.99 (t, *J* = 6.8 Hz, 9H), 0.99 (q, *J* = 7.2 Hz, 6H); ^{13}C NMR (100 MHz, $CDCl_3$) δ (ppm) 167.2, 143.7, 142.2, 138.2, 129.7, 123.2, 121.9, 120.1, 107.9, 25.8, 7.75, 3.68; LRMS (EI, 70 eV) *m/z* (%): 273 (M^+, 2), 244 (100), 186 (12); HRMS *m/z* (ESI) calcd for $C_{16}H_{24}NOSi$ ($[M+H]^+$) 274.1622, found 274.1620.

(*Z*)-1-benzyl-3-((triethylsilyl)methylene)indolin-2-one (4ea):

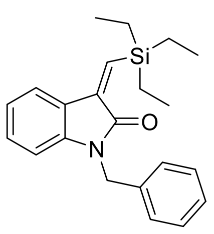

62.8 mg, 90% yield; Colorless oil; R_f = 0.2 (PE/EA = 20 : 1); ^{1}H NMR (400 MHz, $CDCl_3$) δ (ppm) 7.45 (d, *J* = 7.6 Hz, 1H), 7.33-7.23 (m, 5H), 7.15 (t, *J* = 7.6 Hz, 1H), 6.99-6.96 (m, 2H), 6.65 (d, *J* = 7.6 Hz, 1H), 4.94 (s, 1H), 1.01 (t, *J* = 7.2 Hz, 9H), 0.99 (q, *J* = 7.6 Hz, 6H); ^{13}C NMR (100 MHz, $CDCl_3$) δ (ppm) 167.1, 142.8, 142.1, 138.7, 136.1, 129.6, 128.7, 127.5, 127.2, 123.3, 121.9, 120.2, 109.0, 43.5, 7.8, 3.7; LRMS (EI, 70 eV) *m/z* (%): 349 (M^+, 1), 320 (100), 172 (7); HRMS *m/z* (ESI) calcd for $C_{22}H_{28}NOSi$ ($[M+H]^+$) 350.1935, found 350.1940.

(*Z*)-1-(4-methoxybenzyl)-3-((triethylsilyl)methylene)indolin-2-one (4fa):

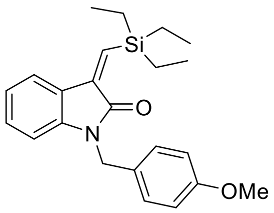

44.5 mg, 60% yield; Colorless oil; R_f = 0.2 (PE/EA = 20 : 1); ^{1}H NMR (400 MHz, $CDCl_3$) δ (ppm) 7.37 (d, *J* = 7.6 Hz, 1H), 7.19 (s, 1H), 7.16 (d, *J* = 8.8 Hz, 2H), 7.09 (t, *J* = 6.4 Hz, 1H), 6.91 (d, *J* = 7.2 Hz, 2H), 6.77 (d, *J* = 8.4 Hz, 2H), 6.61 (d, *J* = 8.0 Hz, 1H), 4.80 (s, 2H), 3.70 (s, 3H), 0.93 (t, *J* = 8.4 Hz, 9H), 0.85 (q, *J* = 7.6 Hz, 6H); ^{13}C NMR (100 MHz, $CDCl_3$) δ (ppm) 167.1, 159.0, 142.9, 142.2, 138.6, 129.6, 128.6, 128.2, 123.4, 121.9, 120.2, 114.1, 109.0, 77.3, 77.0, 76.7, 55.3, 42.9, 7.8, 3.7; LRMS (EI, 70 eV) m/z (%): 379 (M^+, 1), 350 (20), 172 (5), 144 (2), 121 (100), 91 (5); HRMS *m/z* (ESI) calcd for $C_{23}H_{30}NO_2Si$ ($[M+H]^+$) 380.2040, found 380.2046.

(*Z*)-3-((triethylsilyl)methylene)-1-(4-(trifluoromethyl)benzyl)indolin-2-one (4ga):

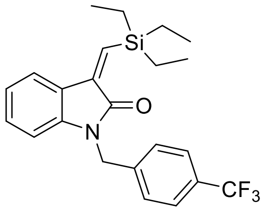

39.2 mg, 47% yield; Colorless oil; R_f = 0.2 (PE/EA = 20 : 1); ^{1}H NMR (500 MHz, $CDCl_3$) δ (ppm) 7.58 (d, *J* = 7.5 Hz, 2H), 7.48 (d, *J* = 7.5 Hz, 1H), 7.40 (d, *J* = 7.5 Hz, 2H), 7.27 (s, 1H), 7.18 (t, *J* = 7.5 Hz, 1H), 7.03 (t, *J* = 9.0 Hz, 2H), 6.61 (d, *J* = 7.5 Hz, 1H), 4.99 (s, 2H), 1.01 (t, *J* = 7.5 Hz, 9H), 0.91 (q, *J* = 8.0 Hz, 6H); ^{13}C NMR (125 MHz, $CDCl_3$) δ (ppm) 167.2, 142.3, 141.7, 140.2, 139.5, 129.8, 129.5, 127.4, 125.8 - 125.7 (m,1C), 123.3, 122.3, 120.4, 108.7, 77.3, 77.0, 76.8, 43.0, 7.8, 3.7; ^{19}F NMR (471 MHz, $CDCl_3$) δ (ppm) -62.5; LRMS (EI, 70 eV) m/z (%): 417 (M^+, 1), 388 (100), 200 (11), 159 (35), 140 (8), 109 (12); HRMS *m/z* (ESI) calcd for $C_{23}H_{27}F_3NOSi$ ($[M+H]^+$) 418.1809, found 418.1812.

(*Z*)-1-(cyclopropylmethyl)-3-((triethylsilyl)methylene)indolin-2-one (4ha):

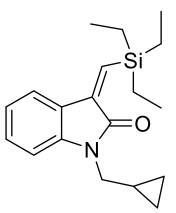

50.7 mg, 81% yield; Colorless oil; R_f = 0.2 (PE/EA = 20 : 1); ^{1}H NMR (400 MHz, $CDCl_3$) δ (ppm) 7.45 (d, *J* = 7.6 Hz, 1H), 7.27 (t, *J* = 7.6 Hz, 1H), 7.00 (t, *J* = 7.6 Hz, 1H), 6.93 (s, 1H), 6.86 (d, *J* = 8.0 Hz, 1H), 3.62 (d, *J* = 7.2 Hz, 2H), 1.20-1.16 (m, 1H), 0.99 (t, *J* = 7.2 Hz, 9H), 0.88 (q, *J* = 7.2 Hz, 6H), 0.54-0.49 (m, 2H), 0.41-0.37 (m, 2H); ^{13}C NMR (100 MHz, $CDCl_3$) δ (ppm) 167.0, 143.4, 142.4, 137.9, 129.6, 123.2, 121.6, 120.2, 108.4, 43.8, 9.8, 7.7, 3.8, 3.7; LRMS (EI, 70 eV) *m/z* (%): 313 (M^+, 2), 284 (100), 172 (18); HRMS *m/z* (ESI) calcd for $C_{22}H_{28}NOSi$ ($[M+H]^+$) 314.1935, found 314.1946.

(*Z*)-1-allyl-3-((triethylsilyl)methylene)indolin-2-one (4ia):

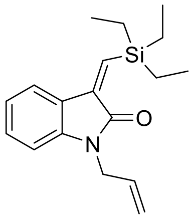

31.1 mg, 52% yield; Colorless oil; R_f = 0.2 (PE/EA = 20 : 1); ^{1}H NMR (400 MHz, $CDCl_3$) δ (ppm) 7.47 (d, *J* = 7.2 Hz, 1H), 7.26 (d, *J* = 7.6 Hz, 1H), 7.04 (t, *J* = 7.6 Hz, 1H), 6.98 (s, 1H), 6.80 (d, *J* = 7.6 Hz, 1H), 5.93 - 5.83 (m, 1H), 5.24 (t, *J* = 8.0 Hz, 2H), 4.39 (d, *J* = 5.2 Hz, 2H), 1.01 (t, *J* = 7.2 Hz, 9H), 0.90 (q, *J* = 6.0 Hz, 6H); ^{13}C NMR (100 MHz, $CDCl_3$) δ (ppm) 166.8, 142.9, 142.1, 138.5, 131.7, 129.6, 123.3, 121.9, 120.2, 117.3, 108.8, 77.3, 77.0, 76.7, 42.1, 7.8, 3.7; LRMS (EI, 70 eV) m/z (%): 299 (M^+, 2), 270 (100), 200 (16), 172 (22), 144 (8), 87 (2); HRMS *m/z* (ESI) calcd for $C_{18}H_{26}NOSi$ ($[M+H]^+$) 300.1778, found 300.1784.

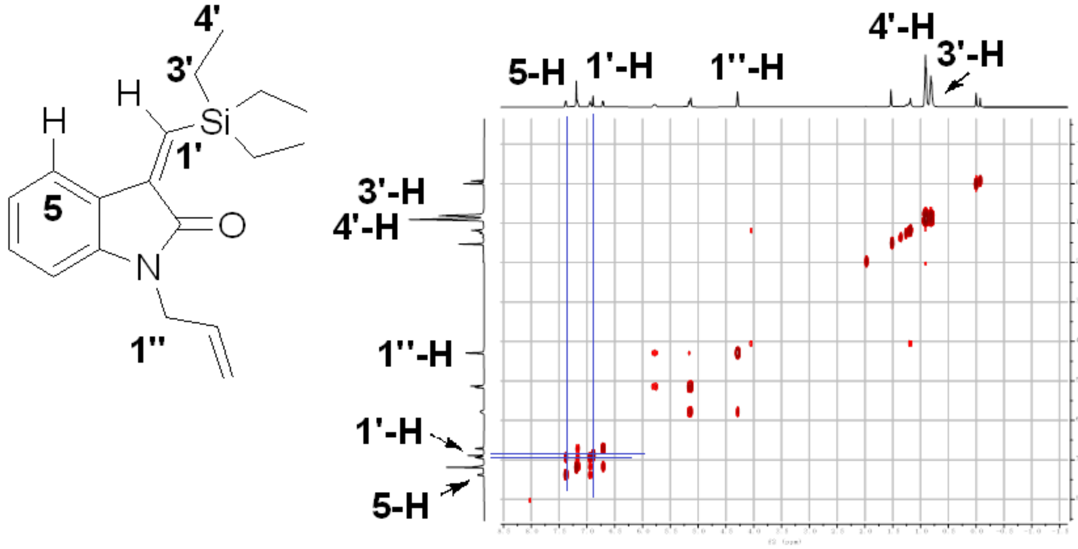

H-H COSY of **4ia**

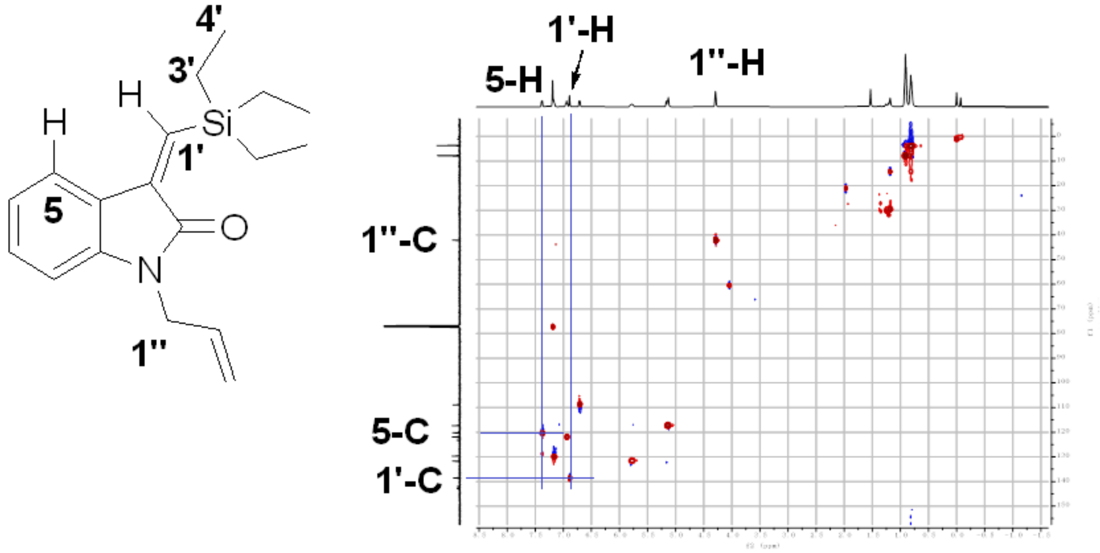

HSQC of **4ia**

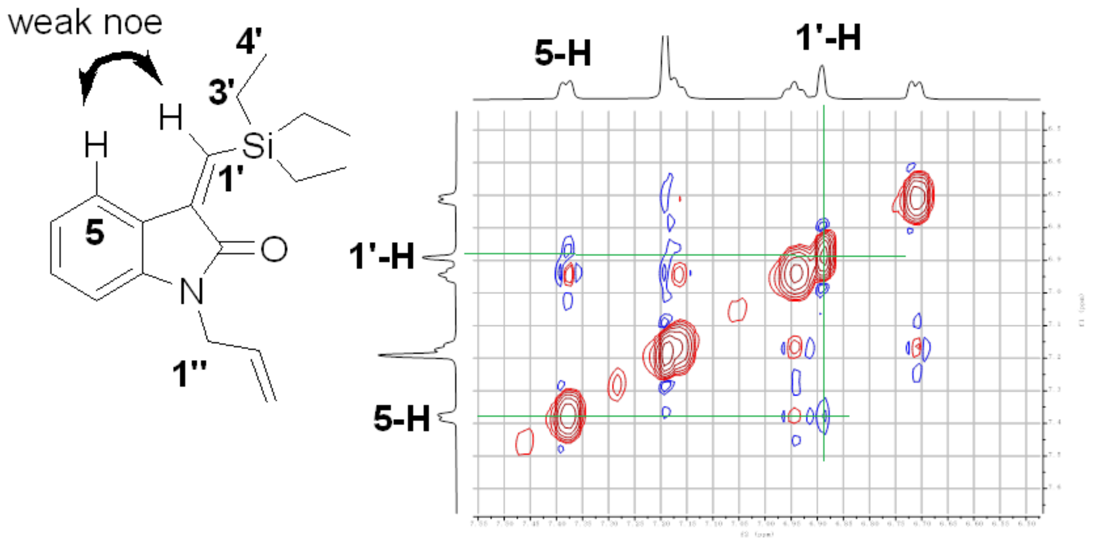

NOE of **4ia**

(*Z*)-1,6-dimethyl-3-((triethylsilyl)methylene)indolin-2-one (4ja):

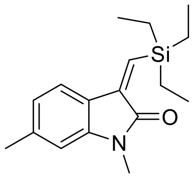

43.7 mg, 82% yield; Colorless oil; R_f = 0.2 (PE/EA = 20 : 1); ^{1}H NMR (400 MHz, $CDCl_3$) δ (ppm) 7.31 (d, *J* = 7.6 Hz, 1H), 6.83 (s, 1H), 6.82 (d, *J* = 8.8 Hz, 1H), 6.59 (s, 1H), 3.20 (s, 3H), 2.39 (s, 3H), 0.98 (t, *J* = 7.6 Hz, 9H), 0.87 (q, *J* = 7.6 Hz, 6H); ^{13}C NMR (100 MHz, $CDCl_3$) δ (ppm) 167.5, 143.8, 142.2, 140.2, 136.5, 122.4, 120.7, 119.9, 108.8, 25.8, 22.1, 7.8, 3.7; LRMS (EI, 70 eV) *m/z* (%): 287 (M^+, 2), 258 (100), 158 (10); HRMS *m/z* (ESI) calcd for $C_{17}H_{26}NOSi$ ($[M+H]^+$) 288.1778, found 288.1788.

(*Z*)-6-fluoro-1-methyl-3-((triethylsilyl)methylene)indolin-2-one (4ka):

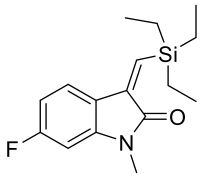

41.9 mg, 75% yield; Colorless oil; R_f = 0.2 (PE/EA = 20 : 1); ^{1}H NMR (400 MHz, $CDCl_3$) δ (ppm) 7.37 (t, *J* = 6.8 Hz, 1H), 6.87 (s, 1H), 6.69 (t, *J* = 8.8 Hz, 1H), 6.51 (d, *J* = 8.0 Hz, 1H), 3.20 (s, 3H), 2.3 (s, 3H), 0.99 (t, *J* = 7.6 Hz, 9H), 0.88 (q, *J* = 7.6 Hz, 6H); ^{13}C NMR (100 MHz, $CDCl_3$) δ (ppm) 167.3, 164.2 (d, *J* = 302.6 Hz), 145.2 (d, *J* = 11.6 Hz), 141.0 (d, *J* = 2.2 Hz), 137.8 (d, *J* = 3.0 Hz), 121.4 (d, *J* = 9.8 Hz), 119.0 (d, *J* = 3.0 Hz), 108.1 (d, *J* = 22.8 Hz), 96.6 (d, *J* = 27.5 Hz), 26.0, 7.7, 3.6; LRMS (EI, 70 eV) *m/z* (%): 291 (M^+, 0.6), 262 (100), 162 (13); HRMS *m/z* (ESI) calcd for $C_{16}H_{23}NOSi$ ($[M+H]^+$) 292.1527, found 292.1534.

(*Z*)-6-chloro-1-methyl-3-((triethylsilyl)methylene)indolin-2-one (4la):

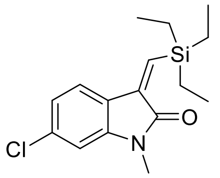

36.9 mg, 67% yield; Colorless oil; R_f = 0.2 (PE/EA = 20 : 1); ^{1}H NMR (400 MHz, $CDCl_3$) δ (ppm) 7.33 (d, *J* = 8.0 Hz, 1H), 6.98 (d, *J* = 8.0 Hz, 1H), 6.94 (s, 1H), 6.77 (s, 1H), 3.20 (s, 1H), 0.98 (t, *J* = 7.6 Hz, 9H), 0.89 (q, *J* = 7.2 Hz, 6H); ^{13}C NMR (100 MHz, $CDCl_3$) δ (ppm) 167.0, 144.6, 141.0, 139.3, 135.4, 121.7, 121.6, 121.0, 108.6, 25.9, 7.7, 3.6; LRMS (EI, 70 eV) *m/z* (%): 307 (M^+, 0.9), 278 (100), 178 (8); HRMS *m/z* (ESI) calcd for $C_{16}H_{23}NOSi$ ($[M+H]^+$) 308.1232, found 308.1233.

(*Z*)-1,5-dimethyl-3-((triethylsilyl)methylene)indolin-2-one (4ma):

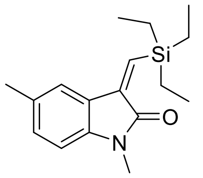

43.1 mg, 80% yield; Colorless oil; R_f = 0.2 (PE/EA = 20 : 1); ^{1}H NMR (400 MHz, $CDCl_3$) δ (ppm) 7.26 (s, 1H), 7.08 (d, *J* = 8.0 Hz, 1H), 6.90 (s, 1H), 6.66 (d, *J* = 8.0 Hz, 1H), 3.20 (s, 3H), 2.3 (s, 3H), 0.98 (t, *J* = 7.2 Hz, 9H), 0.88 (q, *J* = 7.6 Hz, 6H); ^{13}C NMR (100 MHz, $CDCl_3$) δ (ppm) 167.2, 142.5, 141.5, 137.6, 131.3, 130.0, 123.2, 120.8, 107.6, 25.9, 21.0, 7.8, 3.7; LRMS (EI, 70 eV) *m/z* (%): 287 (M^+, 3), 258 (100), 115 (7); HRMS *m/z* (ESI) calcd for $C_{17}H_{26}NOSi$ ($[M+H]^+$) 288.1778, found 288.1791.

(*Z*)-1-methyl-2-oxo-3-((triethylsilyl)methylene)indoline-5-carbonitrile (4na):

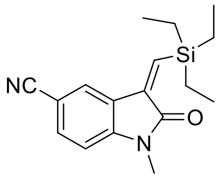

39.9 mg, 71% yield; Colorless oil; R_f = 0.2 (PE/EA = 20 : 1); ^{1}H NMR (400 MHz, $CDCl_3$) δ (ppm) 7.69 (s, 1H), 7.60 (d, *J* = 8.0 Hz, 1H), 7.13 (s, 1H), 6.87 (d, *J* = 8.0 Hz, 1H), 3.27 (s, 3H), 2.3 (s, 3H), 0.98 (t, *J* = 7.6 Hz, 9H), 0.89 (q, *J* = 7.2 Hz, 6H); ^{13}C NMR (100 MHz, $CDCl_3$) δ (ppm) 166.7, 146.7, 143.1, 139.8, 134.2, 123.5, 119.1, 108.4, 105.1, 26.1, 7.7, 3.5; LRMS (EI, 70 eV) *m/z* (%): 298 (M^+, 0.5), 269 (100), 169 (9); HRMS *m/z* (ESI) calcd for $C_{17}H_{26}NOSi$ ($[M+H]^+$) 299.1574, found 299.1580.

(*Z*)-1-methyl-3-((trihexylsilyl)methylene)indolin-2-one (4db):

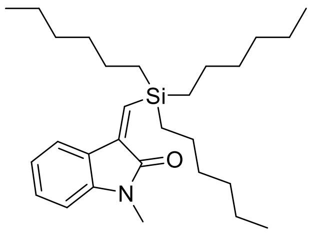

57.6 mg, 73% yield; Colorless oil; R_f = 0.2 (PE/EA = 20 : 1); ^{1}H NMR (400 MHz, $CDCl_3$) δ (ppm) 7.42 (d, *J* = 7.6 Hz, 1H), 7.27 (t, *J* = 7.2 Hz, 1H), 7.01 (t, *J* = 7.6 Hz, 1H), 6.94 (s, 1H), 6.77 (d, *J* = 7.6 Hz, 1H), 3.22 (s, 3H), 1.34-1.26 (m, 24H), 0.88-0.85 (m, 15H); ^{13}C NMR (100 MHz, $CDCl_3$) δ (ppm) 167.0, 143.7, 141.7,

139.1, 129.6, 123.2, 121.8, 120.0, 107.8, 33.5, 31.6, 25.9, 24.1, 22.6, 14.1, 12.6; LRMS (EI, 70 eV) *m/z* (%): 441 (M^+, 0.4), 356 (100), 272 (6); HRMS *m/z* (ESI) calcd for $C_{28}H_{48}NOSi$ ($[M+H]^+$) 442.3500, found 442.3512.

(*Z*)-1-methyl-3-((triisopropylsilyl)methylene)indolin-2-one (4dc):

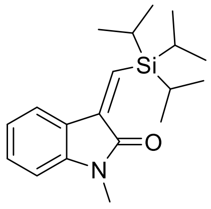

49.1 mg, 78% yield; Colorless oil; R_f = 0.2 (PE/EA = 20 : 1); ^1H NMR (400 MHz, $CDCl_3$) δ (ppm) 7.46 (d, *J* = 7.6 Hz, 1H), 7.29 (t, *J* = 7.2 Hz, 1H), 7.03 (t, *J* = 7.6 Hz, 1H), 6.97 (s, 1H), 6.78 (d, *J* = 7.6 Hz, 1H), 3.23 (s, 3H), 1.59-1.54 (m, 3H), 1.11 (d, *J* = 9.2 Hz, 9H); ^{13}C NMR (100 MHz, $CDCl_3$) δ (ppm) 167.0, 143.4, 142.9, 137.1, 129.7, 123.4, 121.8, 119.9, 107.9, 25.9, 19.2, 12,3; LRMS (EI, 70 eV) *m/z* (%): 315 (M^+, 0.2), 272 (100), 144 (15); HRMS *m/z* (ESI) calcd for $C_{19}H_{30}NOSi$ ($[M+H]^+$) 316.2091, found 316.2098.

(*Z*)-3-((tert-butyldimethylsilyl)methylene)-1-methylindolin-2-one (4dd):

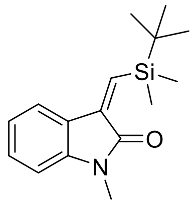

43.7 mg, 80% yield; Colorless oil; R_f = 0.2 (PE/EA = 20 : 1); ^1H NMR (400 MHz, $CDCl_3$) δ (ppm) 7.44 (d, *J* = 7.6 Hz, 1H), 7.28 (t, *J* = 7.6 Hz, 1H), 7.03 (s, 1H), 7.01 (d, *J* = 7.6 Hz, 1H), 6.77 (t, *J* = 7.6 Hz, 1H), 3.22 (s, 3H), 1.00 (s, 9H), 0.31 (s, 6H); ^{13}C NMR (100 MHz, $CDCl_3$) δ (ppm) 167.1, 143.6, 142.3, 138.0, 129.8, 123.2, 121.9, 120.0, 107.9, 26.5, 25.8, 17.1, - 6.2; LRMS (EI, 70 eV) *m/z* (%): 273 (M^+, 0.1), 216 (100), 157 (2); HRMS *m/z* (ESI) calcd for $C_{16}H_{24}NOSi$ ($[M+H]^+$) 274.1622, found 274.1618.

(*Z*)-3-((allyldimethylsilyl)methylene)-1-methylindolin-2-one (4de):

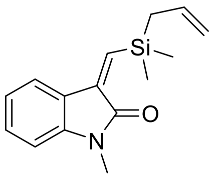

32.4 mg, 65% yield; Colorless oil; R_f = 0.2 (PE/EA = 20 : 1); ^1H NMR (400 MHz, $CDCl_3$) δ (ppm) 7.42 (d, *J* = 7.6 Hz, 1H), 7.28 (t, *J* = 7.2 Hz, 1H), 7.01 (t, *J* = 7.6 Hz, 1H), 6.94 (s, 1H), 6.77 (d, *J* = 7.6 Hz, 1H), 5.90-5.79 (m, 1H), 4.92 (d, *J* = 16.8 Hz, 1H), 4.86 (d, *J* = 10.4 Hz, 1H), 3.22 (s, 3H), 1.87 (d, *J* = 8.4 Hz, 1H), 0.32 (s, 6H); ^{13}C NMR (100 MHz, $CDCl_3$) δ (ppm) 167.1, 143.9, 141.7, 138.5, 135.0, 129.9, 123.0, 122.0, 120.2, 113.2, 108.0, 25.8, 23.4, 3.1; LRMS (EI, 70 eV) *m/z* (%): 257 (M^+, 0.3), 216 (100), 142 (4); HRMS *m/z* (ESI) calcd for $C_{15}H_{20}NOSi$ ($[M+H]^+$) 258.1309, found 258.1315.

(*Z*)-3-((dimethyl(phenyl)silyl)methylene)-1-methylindolin-2-one (4df):

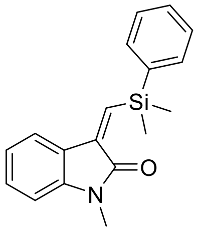

41.0 mg, 70% yield; Colorless oil; R_f = 0.2 (PE/EA = 20 : 1); ^{1}H NMR (400 MHz, $CDCl_3$) δ (ppm) 7.67-7.66 (m, 2H), 7.41-7.38 (m, 4H), 7.26 (d, *J* = 8.4 Hz, 1H), 7.06 (s, 1H), 7.00 (t, *J* = 7.6 Hz, 1H), 6.77 (d, *J* = 8.0 Hz, 1H), 3.21 (s, 3H), 0.61 (s, 6H); ^{13}C NMR (100 MHz, $CDCl_3$) δ (ppm) 167.0, 144.0, 142.1, 139.4, 138.2, 133.8, 130.0, 128.9, 127.8, 123.0, 122.0, 120.3, 108.0, 25.9, 2.6; LRMS (EI, 70 eV) *m/z* (%): 293 (M^+, 64), 216 (100), 186 (23); HRMS *m/z* (ESI) calcd for $C_{18}H_{20}NOSi$ ($[M+H]^+$) 294.1309, found 294.1303.

(*Z*)-1-methyl-3-((methyldiphenylsilyl)methylene)indolin-2-one (4dg):

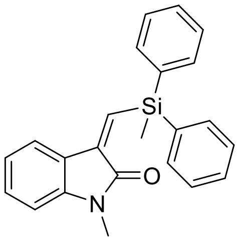

48.3 mg, 68% yield; Colorless oil; R_f = 0.2 (PE/EA = 20 : 1); ^{1}H NMR (400 MHz, $CDCl_3$) δ (ppm) 7.62-7.60 (m, 4H), 7.45 (d, *J* = 8.4 Hz, 1H), 7.36-7.34 (m, 6H), 7.27 (t, *J* = 7.6 Hz, 1H),7.23 (s, 1H), 6.99 (t, *J* = 7.6 Hz, 1H), 6.74 (d, *J* = 8.0 Hz, 1H), 3.13 (s, 3H), 0.93 (s, 3H); ^{13}C NMR (100 MHz, $CDCl_3$) δ (ppm) 166.5, 144.2, 143.2, 137.2, 135.4, 134.5, 130.3, 129.1, 127.8, 122.9, 122.0, 120.6, 108.0, 25.9, 4.0; LRMS (EI, 70 eV) *m/z* (%): 355 (M^+, 26), 278 (100), 138 (8); HRMS *m/z* (ESI) calcd for $C_{23}H_{22}NOSi$ ($[M+H]^+$) 356.1465, found 356.1480.

(*Z*)-1-methyl-3-((triphenylsilyl)methylene)indolin-2-one (4dh):

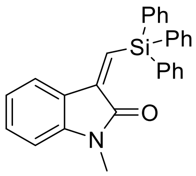

52.5 mg, 63% yield; Colorless oil; R_f = 0.2 (PE/EA = 20 : 1); ^{1}H NMR (400 MHz, $CDCl_3$) δ (ppm) 7.64-7.58 (m, 6H), 7.48 (d, *J* = 7.6 Hz, 1H), 7.38-7.32 (m, 10H), 7.24 (t, *J* = 7.6 Hz, 1H), 6.97 (t, *J* = 7.6 Hz, 1H), 6.68 (d, *J* = 8.0 Hz, 1H), 3.0 (s, 3H); ^{13}C NMR (100 MHz, $CDCl_3$) δ (ppm) 166.0, 144.4, 144.1, 135.6, 134.9, 134.8, 132.9, 130.5, 129.9, 129.2, 127.8, 127.7, 122.8, 127.7, 122.8, 121.9, 120.7, 108.0, 25.9; LRMS (EI, 70 eV) *m/z* (%): 417 (M^+, 16), 416 (100), 218 (11); HRMS *m/z* (ESI) calcd for $C_{28}H_{24}NOSi$ ($[M+H]^+$) 418.1622, found 418.1625.

N-benzyl-N-(2-((*E*)-3-oxo-1-(triethylsilyl)prop-1-en-2-yl)phenyl)cinnamamide (5eab):

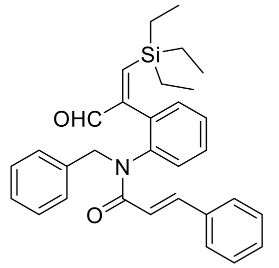

57.7 mg, 50% yield (*E/Z* = >99 : 1); Colorless oil; R_f = 0.2 (PE/EA = 10 : 1); ^{1}H NMR (500 MHz, $CDCl_3$) δ (ppm) 9.88 (s, 1H), 7.67 (d, *J* = 15.5 Hz, 1H), 7.37 (t, *J* = 6.5 Hz, 1H), 7.31 (s, 2H), 7.25 (t, *J* = 14.0 Hz, 10H), 6.97 (s, 1H), 6.79 (d, *J* = 7.5 Hz, 1H), 6.29 (d, *J* = 15.5 Hz, 1H), 5.66 (d, *J* = 14.0 Hz, 1H), 3.88 (d, *J* = 14.0 Hz, 1H), 0.96 (t, *J* = 7.0 Hz, 9H), 0.72 (q, *J* = 7.0 Hz, 6H); ^{13}C NMR (125 MHz, $CDCl_3$) δ (ppm) 191.7, 165.9, 155.1, 154.3, 142.4, 139.5, 137.6, 137.4, 135.1, 131.1, 129.8, 129.6, 129.1, 129.1, 128.7, 128.4, 128.2, 127.9, 127.4, 119.0, 77.3, 77.1, 76.8, 52.5, 7.4, 5.2; LRMS (EI, 70 eV) m/z (%): 481 (M^+, 1), 356 (26), 264 (5), 220 (17), 197 (29), 91 (100); HRMS *m/z* (ESI) calcd for $C_{31}H_{36}NO_2Si$ ($[M+H]^+$) 482.2510, found 304. 482.2524.

(*E*)-N-methyl-N-(2-(3-oxo-1-(triethylsilyl)prop-1-en-2-yl)phenyl)formamide (5dad)

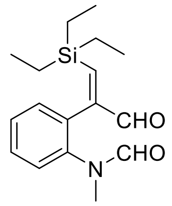

37.6 mg, 62% yield (*E/Z* = >99 : 1); Colorless oil; R_f = 0.2 (PE/EA = 20 : 1); ^{1}H NMR (500 MHz, $CDCl_3$) δ (ppm) 9.88 (s, 1H), 8.03 (s, 1H), 7.41 (t, *J* = 8.5 Hz, 1H), 7.37 (t, *J* = 8.0 Hz, 1H), 7.21 (t, *J* = 7.5 Hz, 2H), 7.08 (s, 1H), 3.09 (s, 3H), 1.03 (t, *J* = 7.0 Hz, 9H), 0.81 (q, *J* = 7.5 Hz, 6H); ^{13}C NMR (125 MHz, $CDCl_3$) δ (ppm) 191.2, 163.1, 155.4, 154.9, 139.8, 136.4, 131.0, 129.6, 128.1, 127.1, 77.3, 77.1, 76.8, 33.1, 7.5, 5.2; LRMS (EI, 70 eV) m/z (%): 303 (M^+, 1), 274 (58), 246 (38), 204 (79), 172 (100), 144 (82); HRMS *m/z* (ESI) calcd for $C_{17}H_{26}NO_2Si$ ($[M+H]^+$) 304.1727, found 304.1729.

(Z)-1-benzyl-3-propylideneindolin-2-one (6ea):

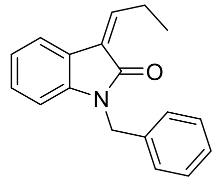

26.3 mg, 40% yield; Colorless oil; R_f = 0.2 (PE/EA = 20 : 1); ^{1}H NMR (400 MHz, $CDCl_3$) δ (ppm) 7.57 (d, *J* = 7.2 Hz, 1H), 7.32 (d, *J* = 4.4 Hz, 4H), 7.29 – 7.25 (m, 1H), 7.20 – 7.11 (m, 2H), 7.03 (t, *J* = 7.6 Hz, 1H), 6.74 (d, *J* = 8.0 Hz, 1H), 4.98 (s, 2H), 2.79 – 2.72 (m, 2H), 1.30 (t, *J* = 7.6 Hz, 3H); ^{13}C NMR (100 MHz, $CDCl_3$) δ (ppm) 168.1, 144.0, 142.7, 136.1, 128.7,

128.7, 127.5, 127.3, 127.0, 123.5, 122.4, 122.1, 109.1, 77.4, 77.0, 76.7, 43.6, 22.8, 13.1. HRMS *m/z* (ESI) calcd for $C_{18}H_{18}NO$ ($[M+H]^+$) 264.1383, found 264.1384.

1-benzyl-3-hydroxy-3-((triethylsilyl)methyl)indolin-2-one (7ea):

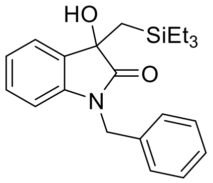

49.9 mg, 68% yield; Colorless oil; R_f = 0.2 (PE/EA = 5 : 1); ^1H NMR (400 MHz, $CDCl_3$) δ (ppm) 7.21 (d, *J* = 7.2 Hz, 1H), 7.15 - 7.10 (m, 4H), 7.08 (d, *J* = 4.4 Hz, 1H), 7.03 (t, *J* = 6.8 Hz, 1H), 6.86 (t, *J* = 7.6 Hz, 1H), 6.56 (d, *J* = 7.6 Hz, 1H), 4.88 (d, *J* = 15.6 Hz, 1H), 4.49 (d, *J* = 15.2 Hz, 1H), 2.69 (s, 1H), 1.39 (q, *J* = 8.0 Hz, 2H), 0.60 (t, *J* = 8.0 Hz, 9H), 0.14 – -0.06 (m, 6H); ^{13}C NMR (100 MHz, $CDCl_3$) δ (ppm) 178.5, 142.1, 135.4, 131.4, 129.6, 128.8, 127.7, 127.6, 124.0, 123.0, 109.5, 77.4, 77.0, 76.7, 75.7, 43.9, 23.3, 7.2, 3.4. HRMS *m/z* (ESI) calcd for $C_{22}H_{30}NO_2Si$ ($[M+H]^+$) 368.2040, found 368.2045.

(*E*)- and (*Z*)-N-methyl-N-(2-(2-(triethylsilyl)vinyl)phenyl)formamide (8xa):

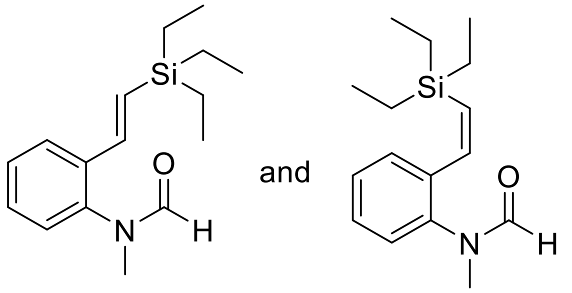

27.5 mg, 50% yield; (*E/Z* = 6.7 : 1); Colorless oil; R_f = 0.2 (PE/EA = 20 : 1); ^1H NMR (400 MHz, $CDCl_3$) δ (ppm) 8.11 (s, 1H), 8.10 (s, 0.41H), 7.59 (d, *J* = 7.6 Hz, 1H), 7.33 – 7.22 (m, 4H), 7.19 (s, 1H), 7.06 – 7.03 (m, 1.5H), 6.79 (d, *J* = 19.6 Hz, 1H), 6.42 (d, *J* = 19.2 Hz, 1H), 5.86 (d, *J* = 15.2 Hz, 0.43H), 5.73 (d, *J* = 18.0 Hz, 0.48H), 3.14 (s, 3H), 3.13 (s, 1.3H), 0.90 (t, *J* = 7.6 Hz, 9H), 0.80 (t, *J* = 9.2 Hz, 6H), 0.59 (q, *J* = 8.0 Hz, 6H), 0.46 (q, *J* = 8.0 Hz, 2.6H); ^{13}C NMR (100 MHz, $CDCl_3$) δ (ppm) 163.3, 163.2, 143.3, 139.9, 139.3, 139.2, 137.8, 136.2, 134.0, 132.8, 131.0, 130.0, 129.0, 128.8, 128.4, 127.6, 127.6, 127.0, 126.8, 77.4, 77.1, 76.7, 33.8, 33.3, 7.5, 7.4, 4.6, 3.4; LRMS (EI, 70 eV) m/z (%): 275 (M^+, 1), 246 (7), 145 (17), 144 (100), 94 (6), 87 (5).

(C) Spectra

(*Z*)-1-(4-bromobenzyl)-3-((triethylsilyl)methylene)indolin-2-one (4aa)

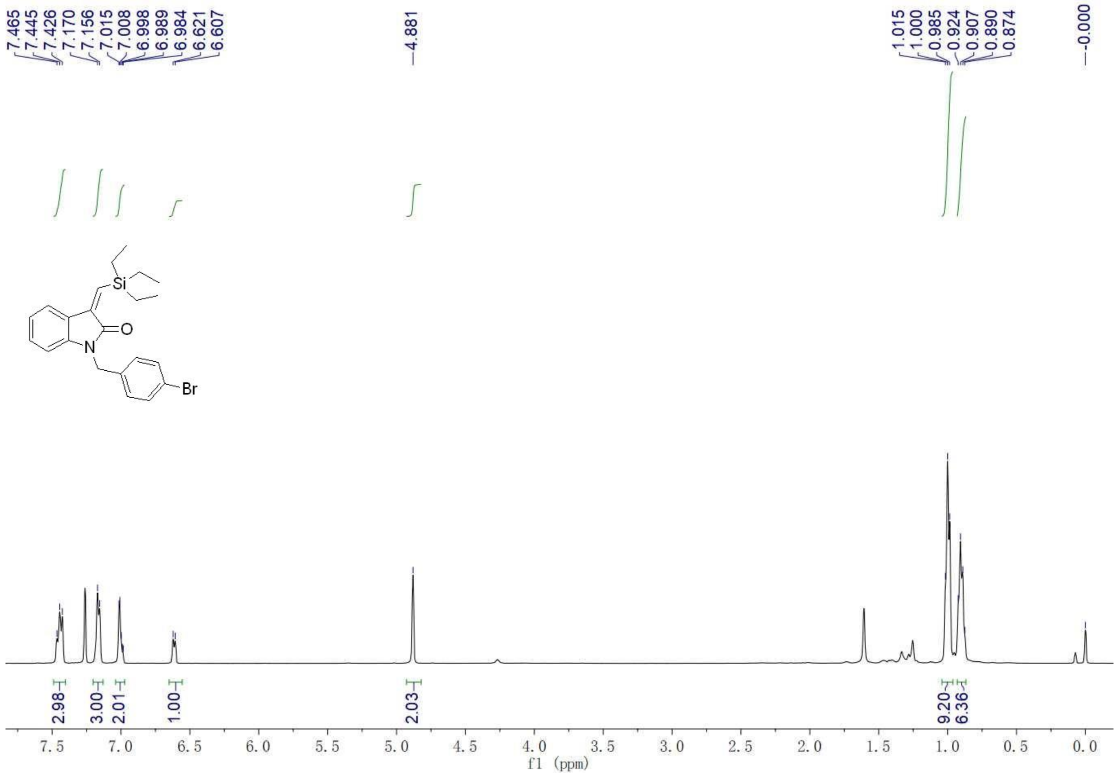

^{1}H-NMR (500 MHz, $CDCl_3$)

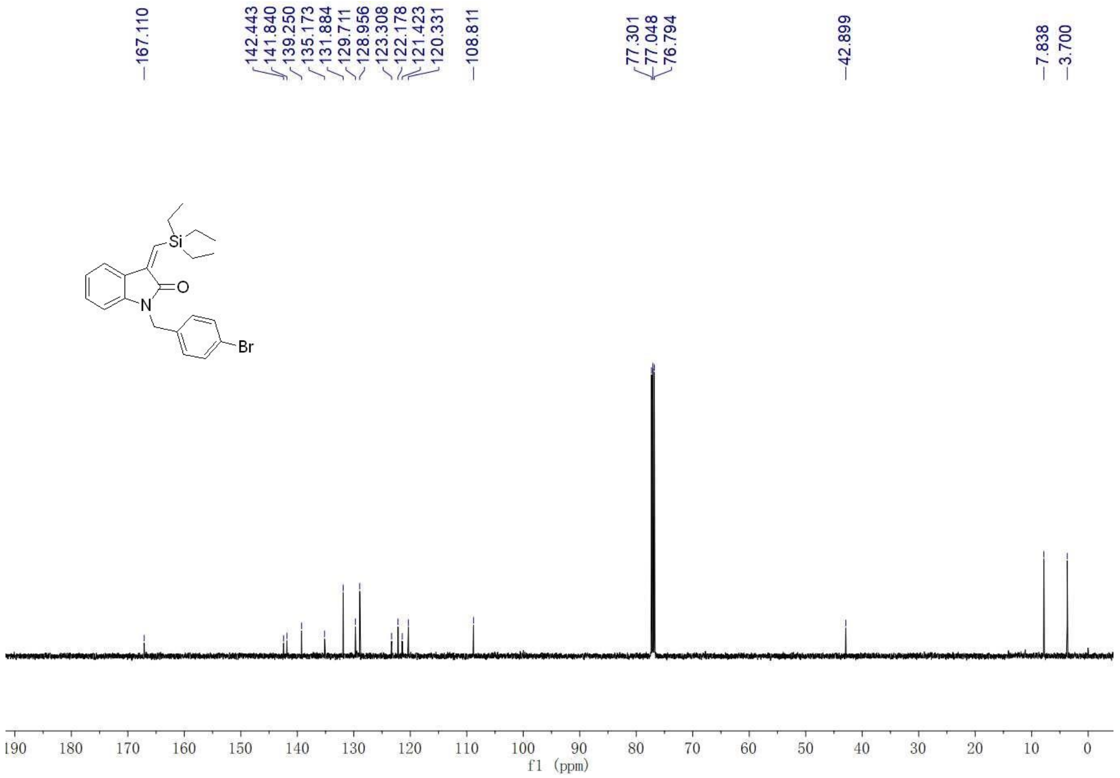

^{13}C-NMR (125 MHz, $CDCl_3$)

(*Z*)-1-methyl-3-((triethylsilyl)methylene)indolin-2-one (4ca)：

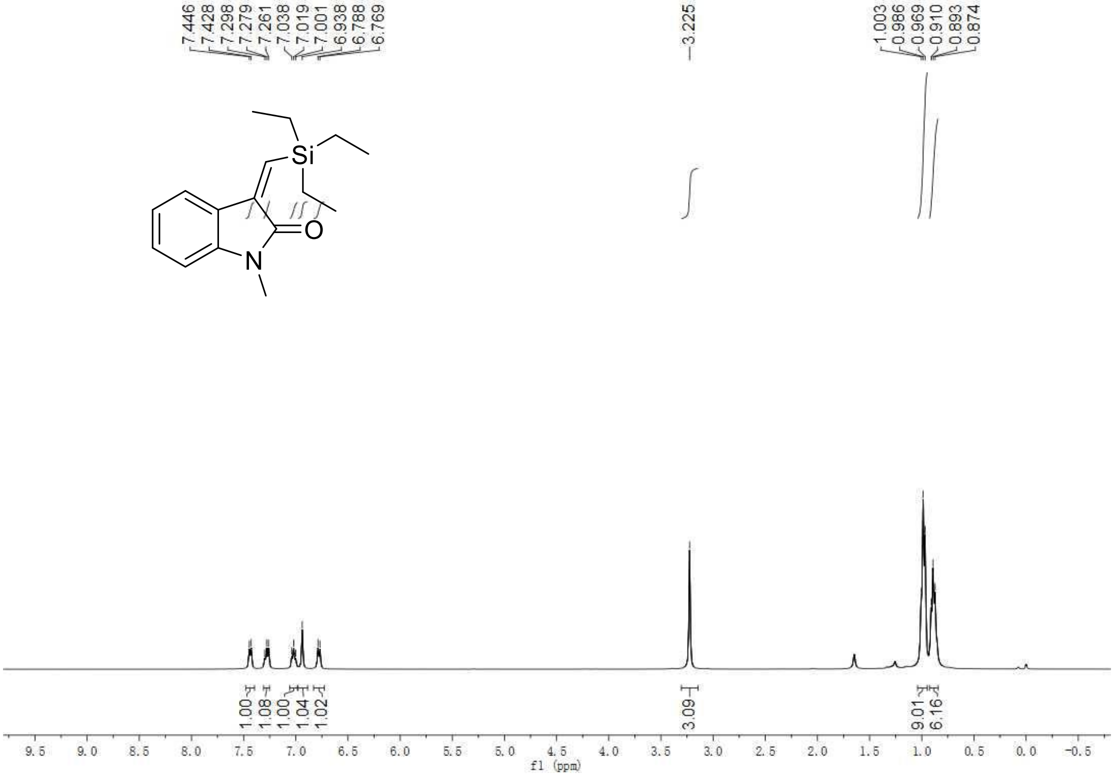

^{1}H-NMR (400 MHz, $CDCl_3$)

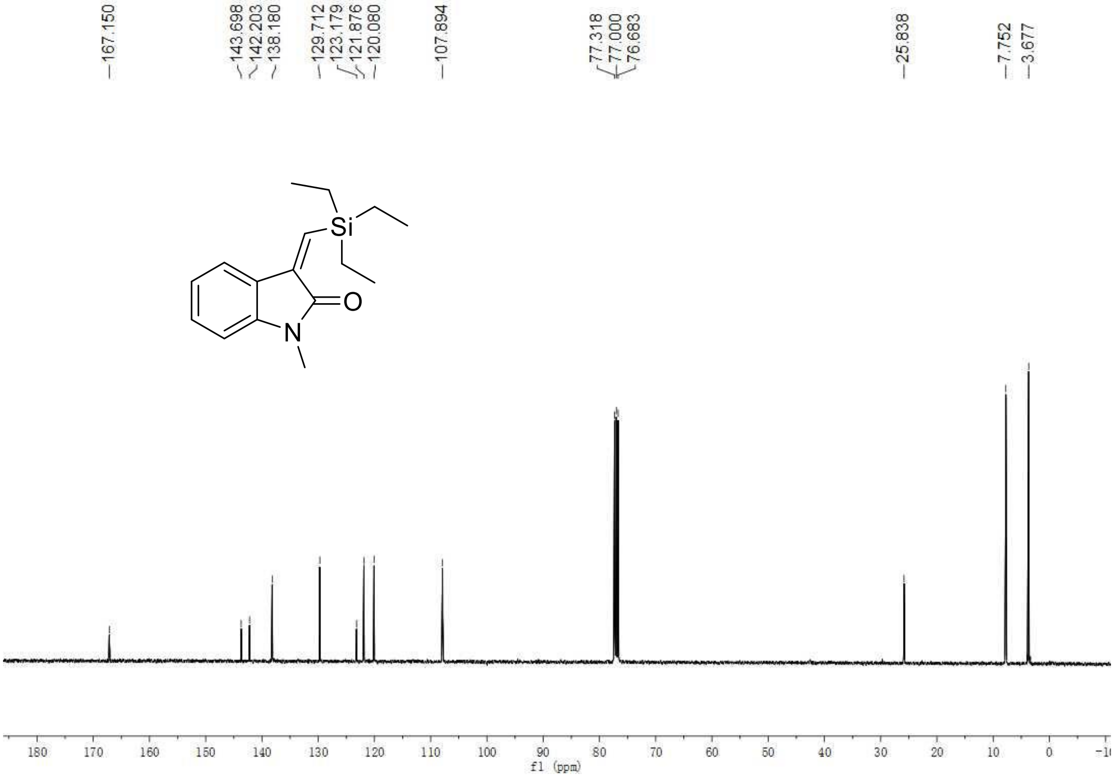

^{13}C-NMR (100 MHz, $CDCl_3$)

(*Z*)-1-benzyl-3-((triethylsilyl)methylene)indolin-2-one (4ea):

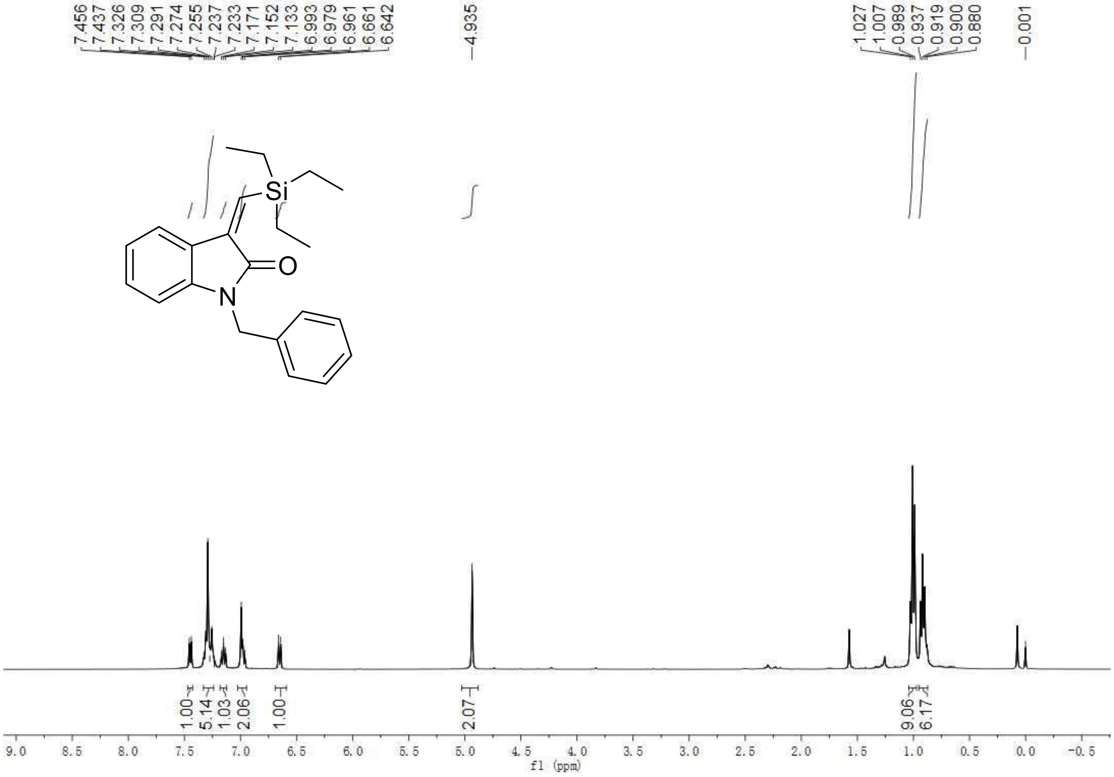

^{1}H-NMR (400 MHz, $CDCl_3$)

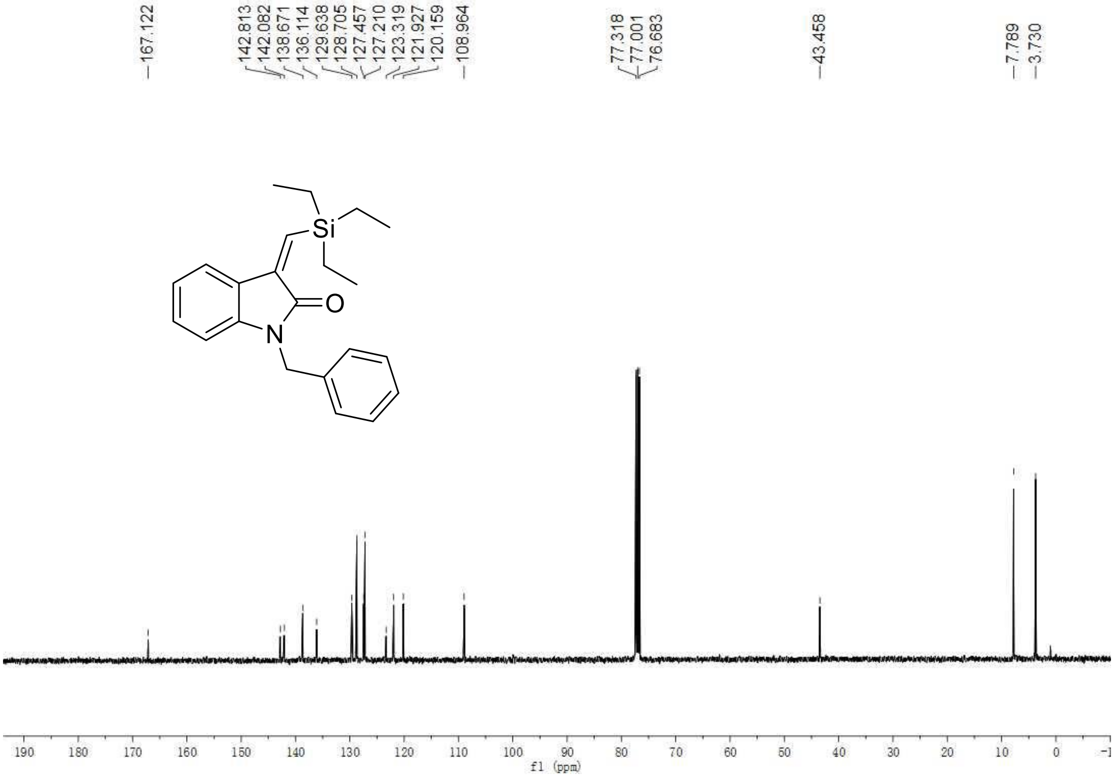

^{13}C-NMR (100 MHz, $CDCl_3$)

(*Z*)-1-(4-methoxybenzyl)-3-((triethylsilyl)methylene)indolin-2-one (4fa)

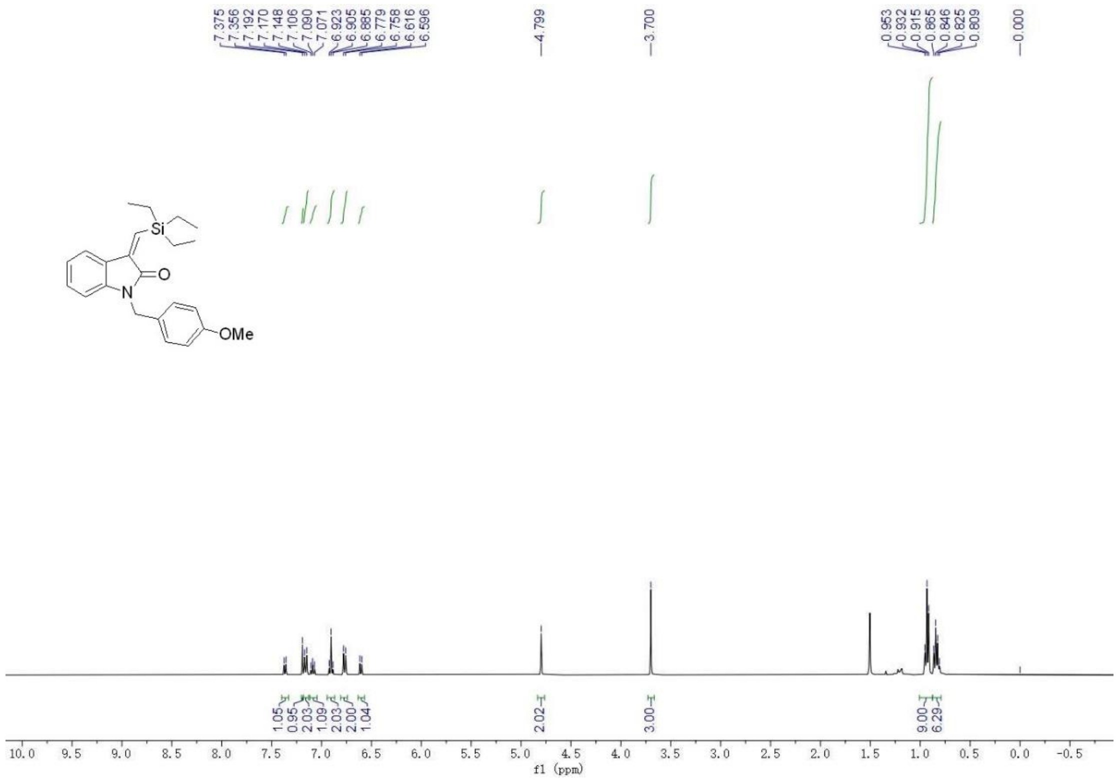

^{1}H-NMR (400 MHz, $CDCl_3$)

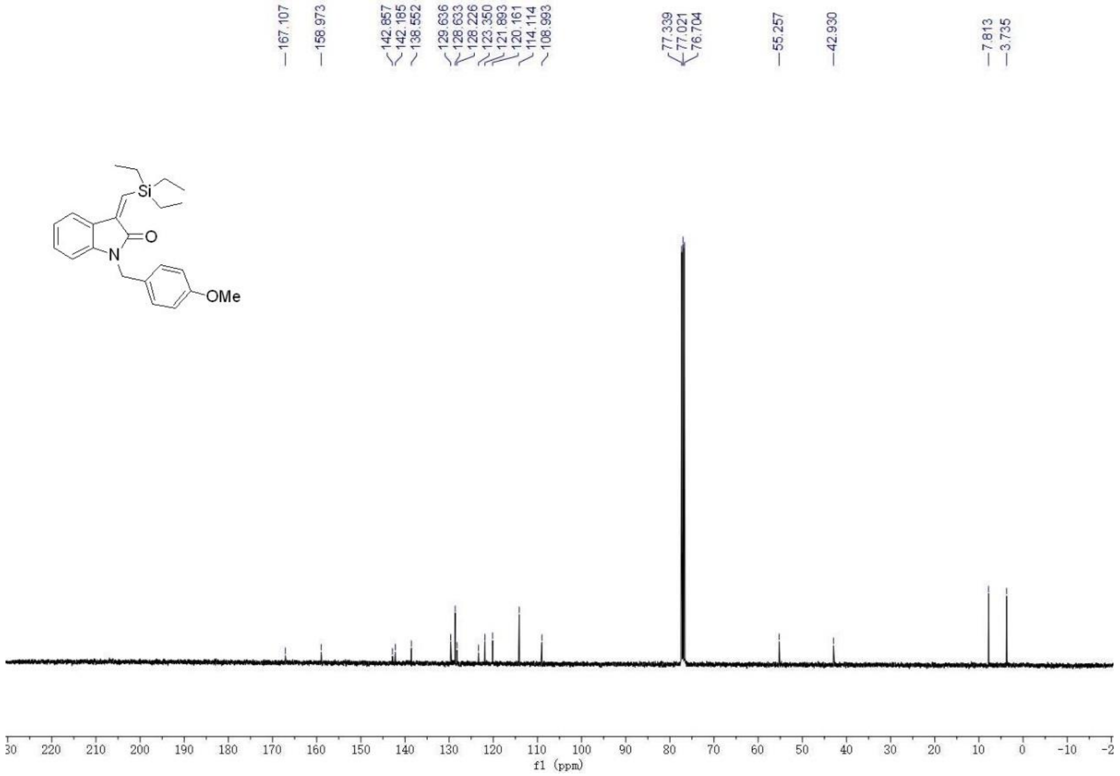

^{13}C-NMR (100 MHz, $CDCl_3$)

(*Z*)-3-((triethylsilyl)methylene)-1-(4-(trifluoromethyl)benzyl)indolin-2-one (4ga)

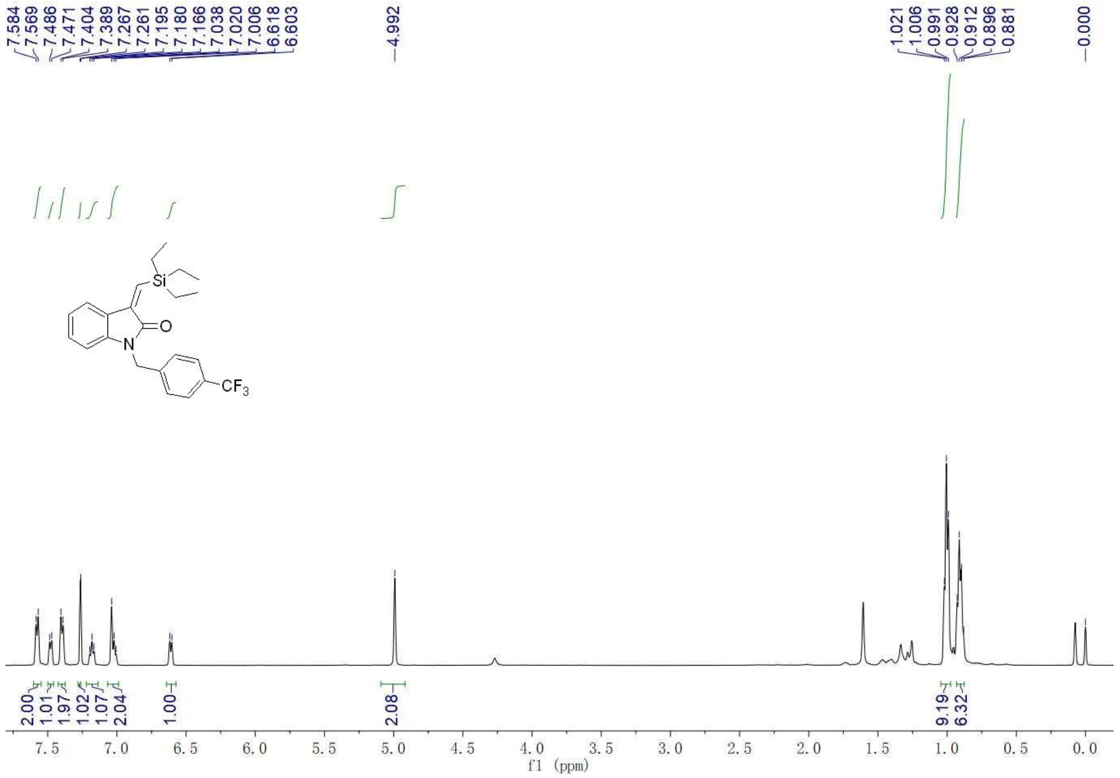

^{1}H-NMR (500 MHz, $CDCl_3$)

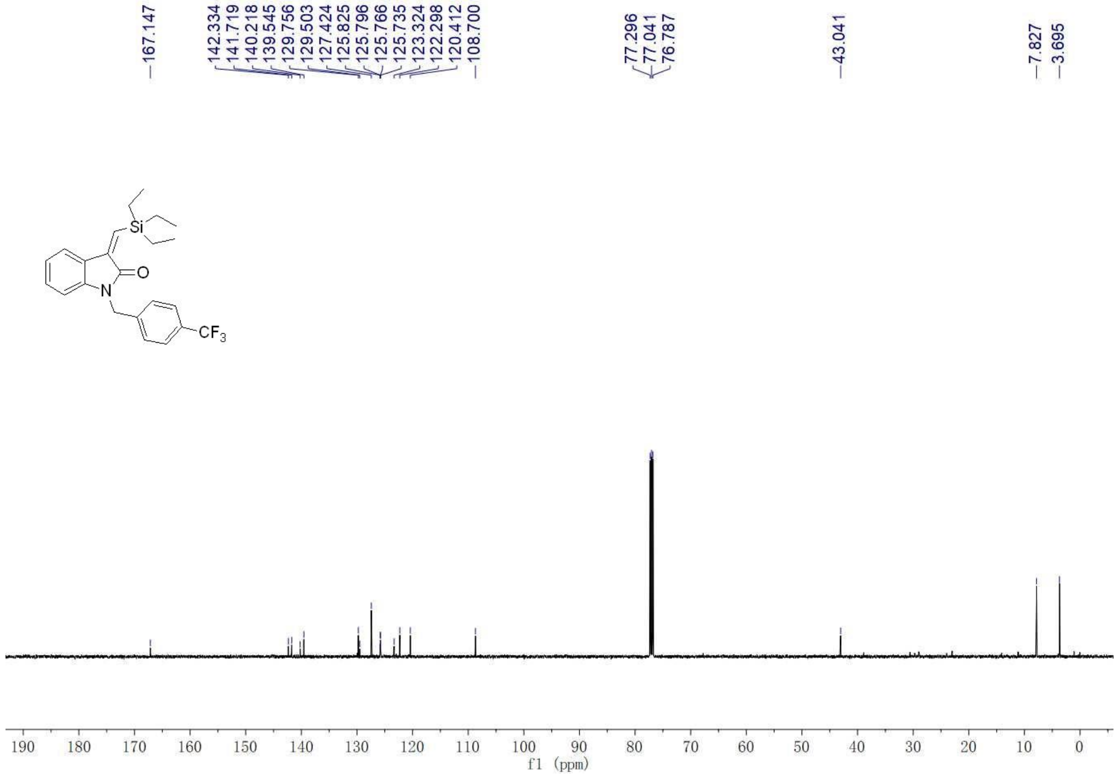

^{13}C-NMR (125 MHz, $CDCl_3$)

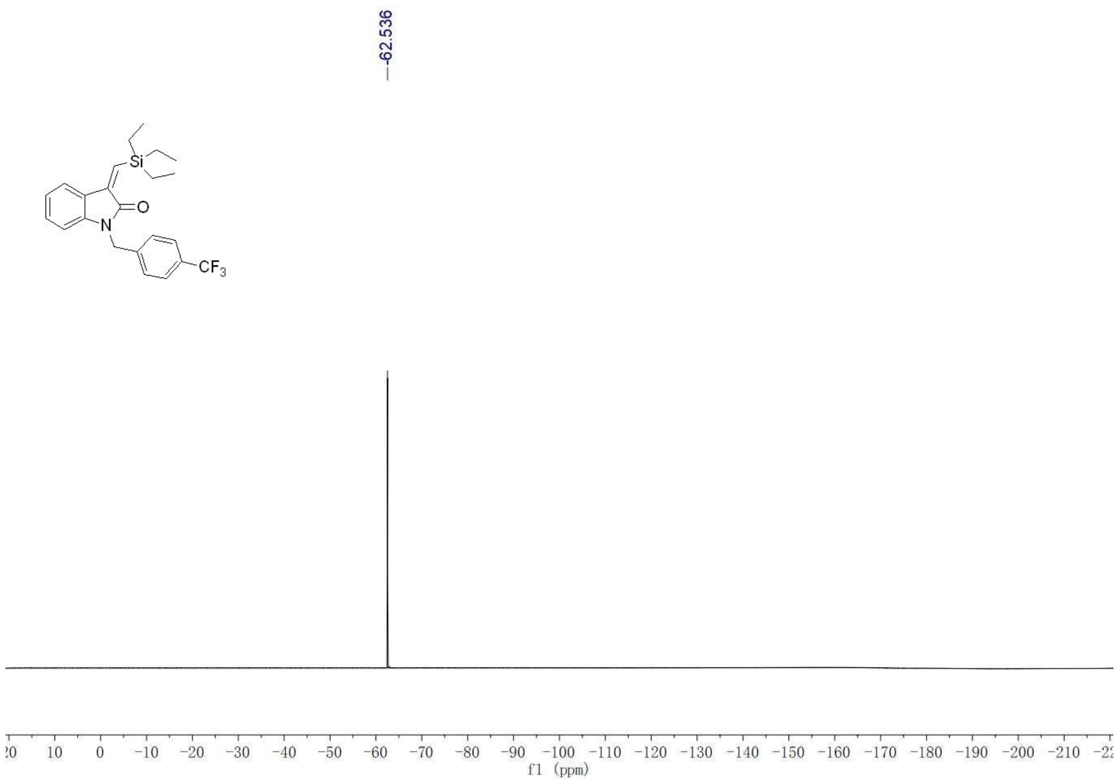

^{19}F-NMR (471 MHz, $CDCl_3$)

(*Z*)-1-(cyclopropylmethyl)-3-((triethylsilyl)methylene)indolin-2-one (4ha):

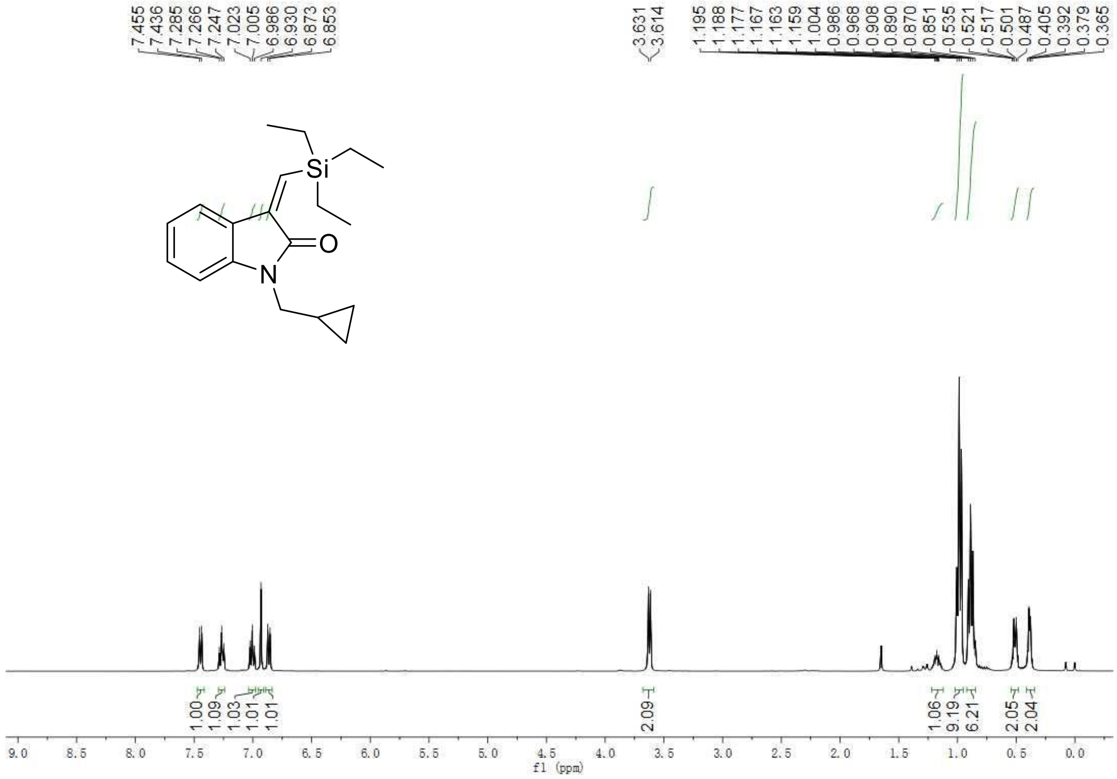

^{1}H-NMR (400 MHz, $CDCl_3$)

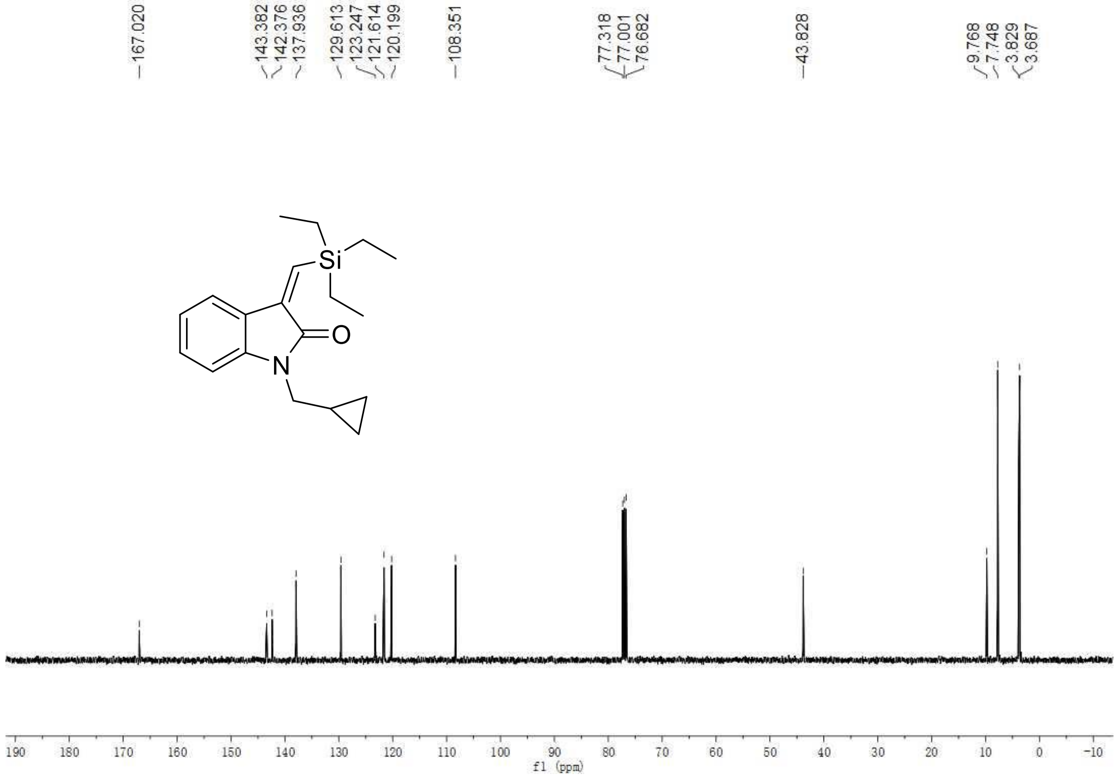

^{13}C-NMR (100 MHz, $CDCl_3$)

(*Z*)-1-allyl-3-((triethylsilyl)methylene)indolin-2-one (4ia)

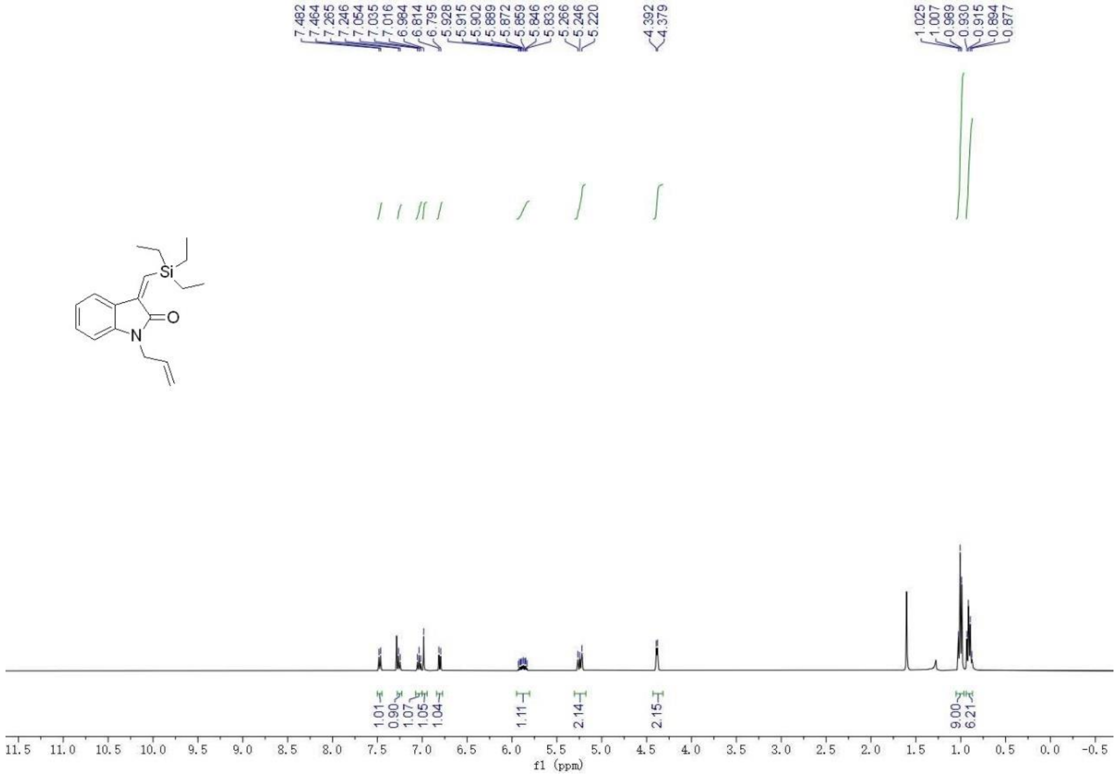

^{1}H-NMR (400 MHz, $CDCl_3$)

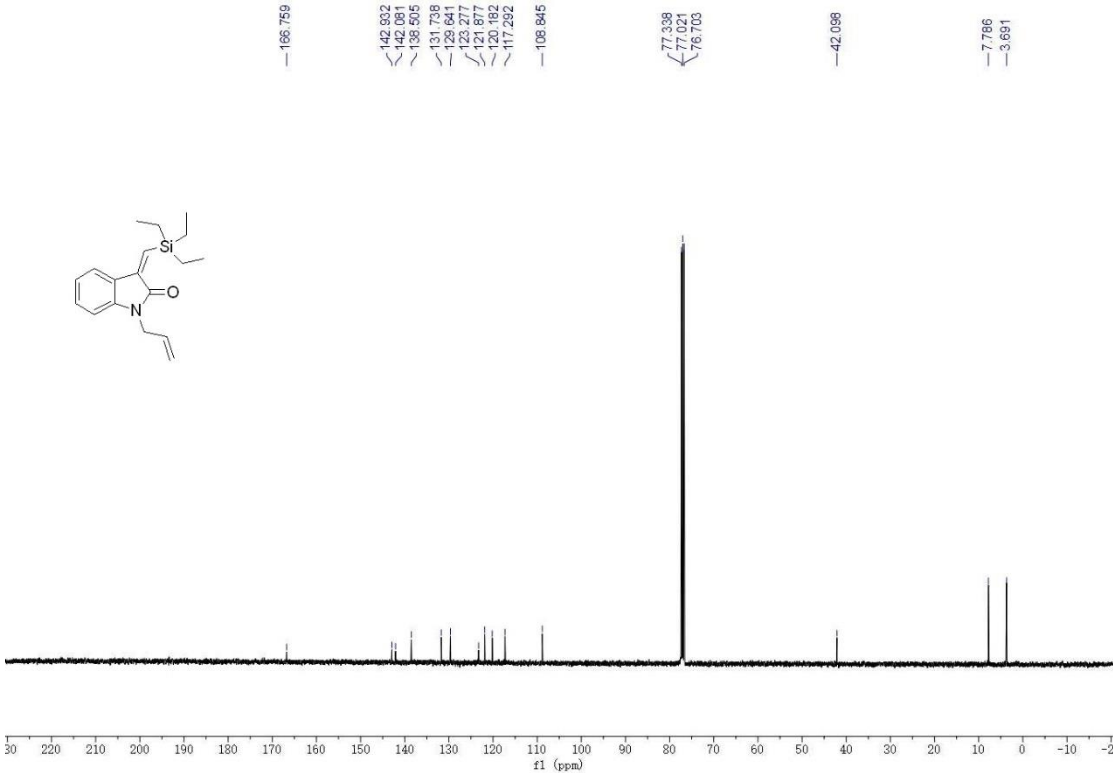

^{13}C-NMR (100 MHz, $CDCl_3$)

(*Z*)-1,6-dimethyl-3-((triethylsilyl)methylene)indolin-2-one (4ja):

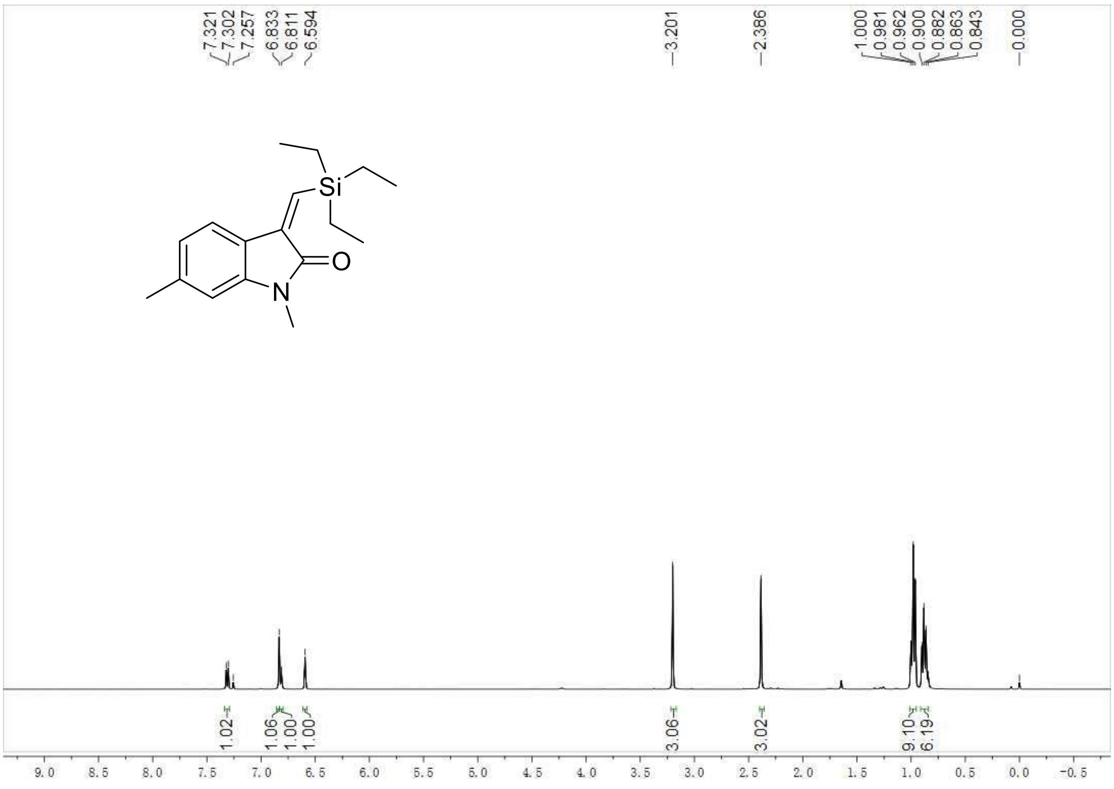

^{1}H-NMR (400 MHz, $CDCl_3$)

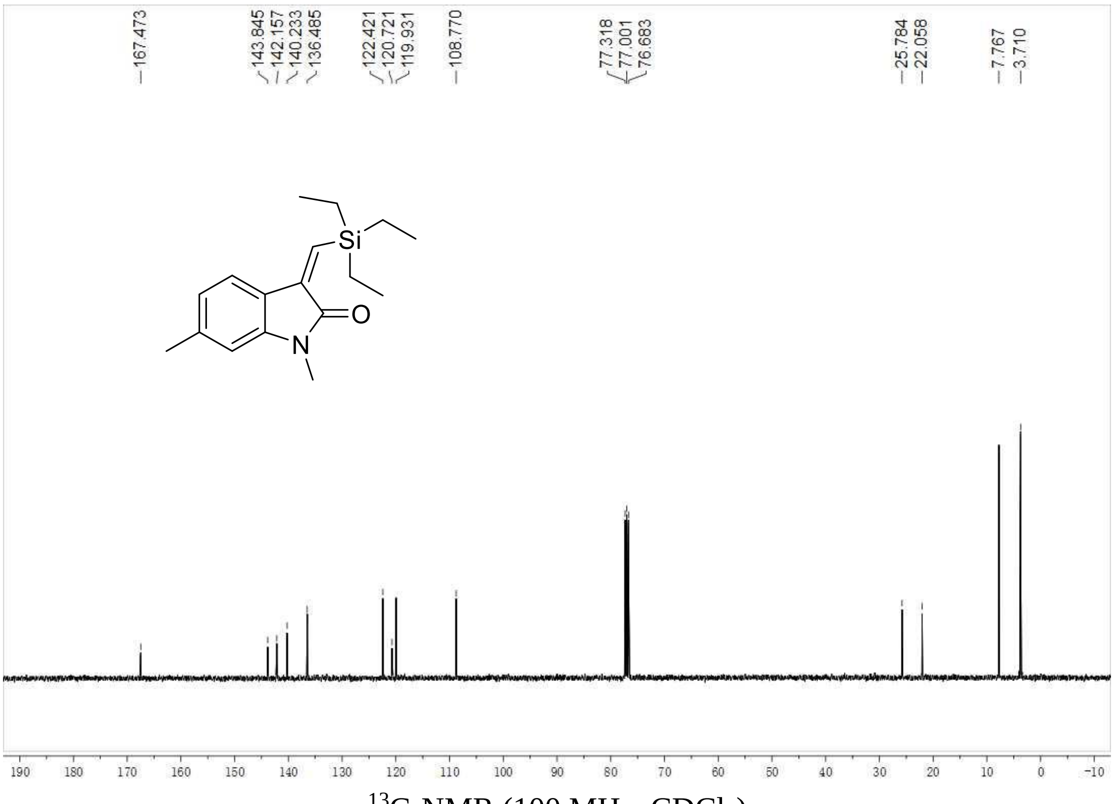

^{13}C-NMR (100 MHz, $CDCl_3$)

(*Z*)-6-fluoro-1-methyl-3-((triethylsilyl)methylene)indolin-2-one (4ka):

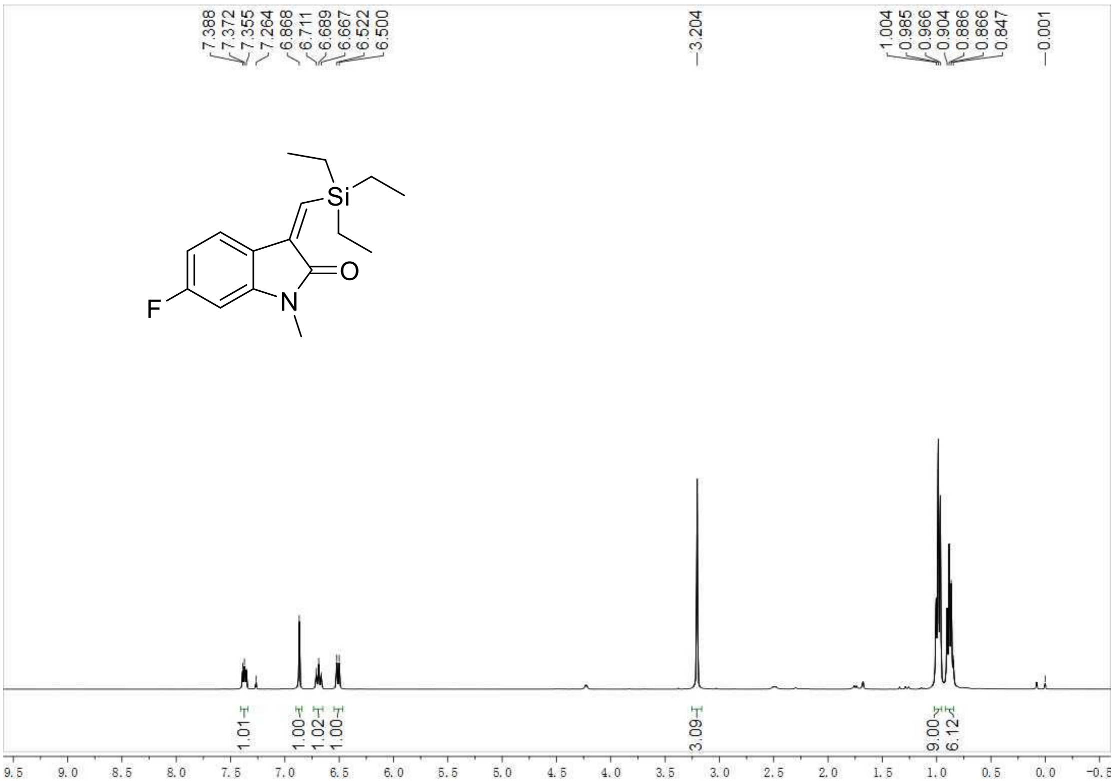

^{1}H-NMR (400 MHz, CDCl$_3$)

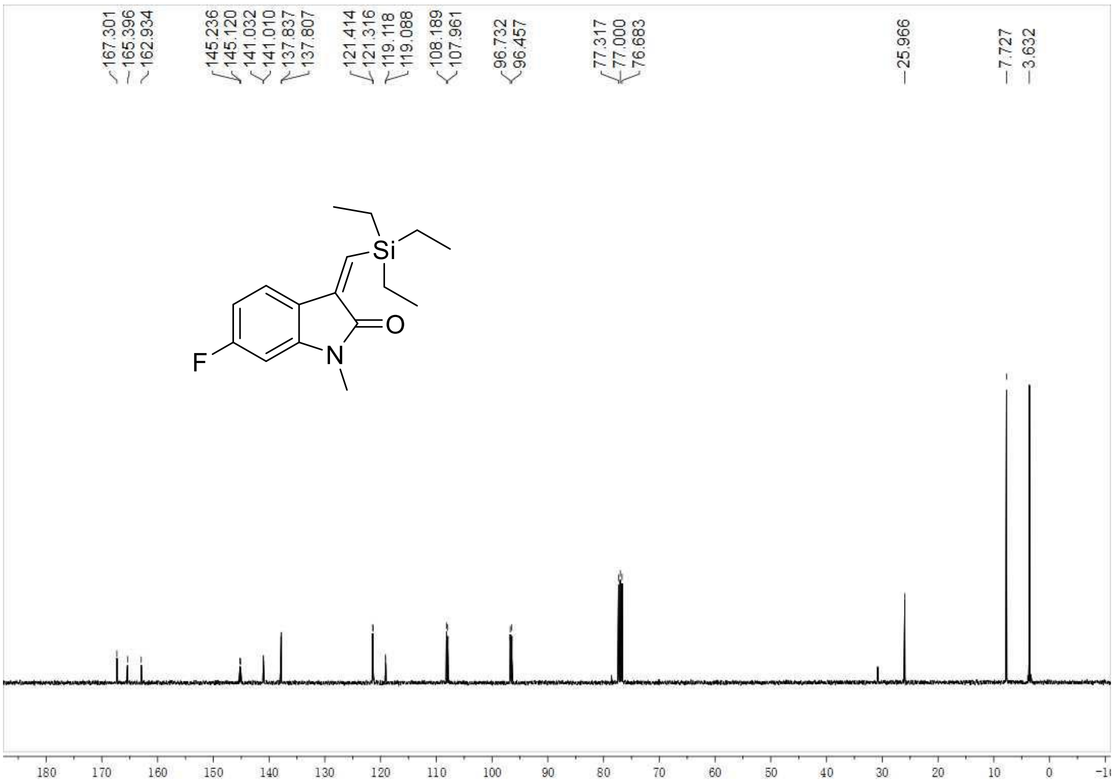

^{13}C-NMR (100 MHz, CDCl$_3$)

(*Z*)-6-chloro-1-methyl-3-((triethylsilyl)methylene)indolin-2-one (4la):

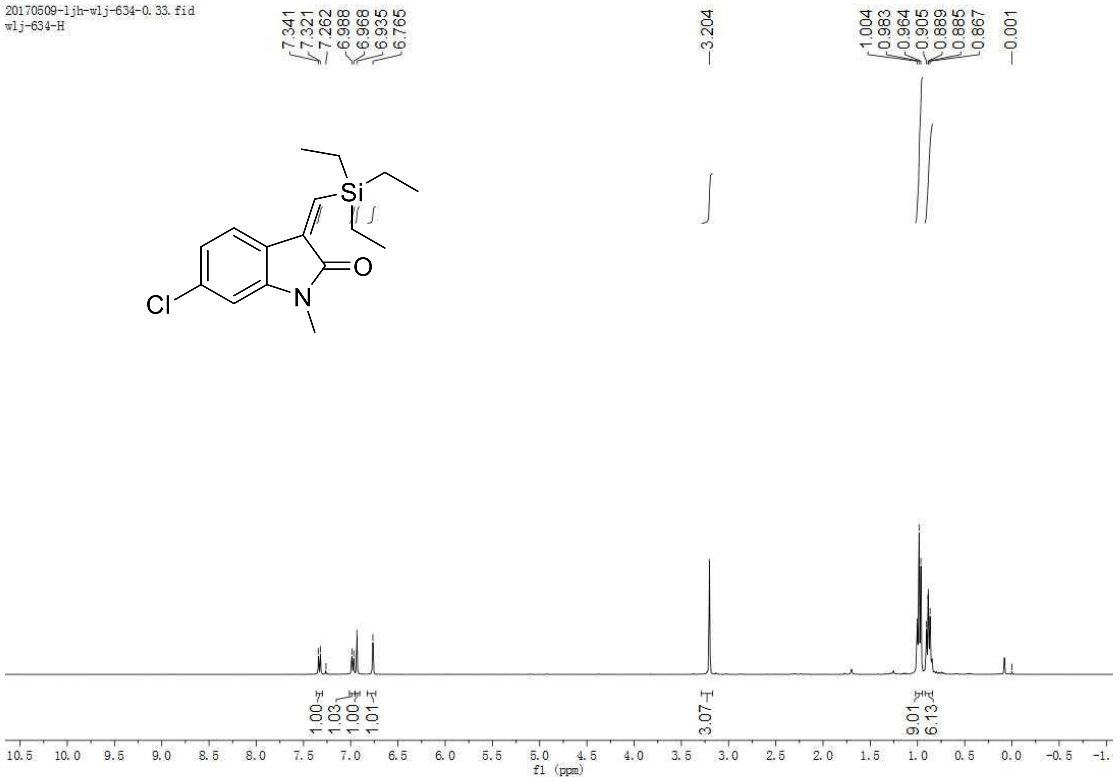

^{1}H-NMR (400 MHz, $CDCl_3$)

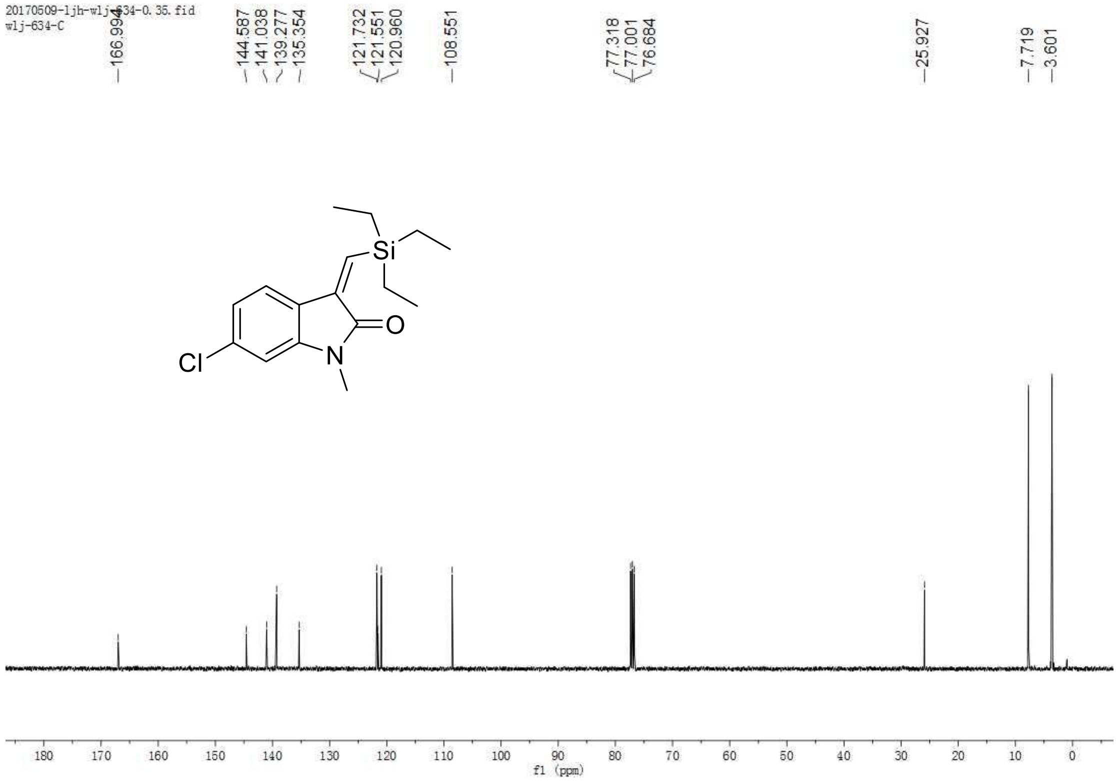

^{13}C-NMR (100 MHz, $CDCl_3$)

(*Z*)-1,5-dimethyl-3-((triethylsilyl)methylene)indolin-2-one (4ma):

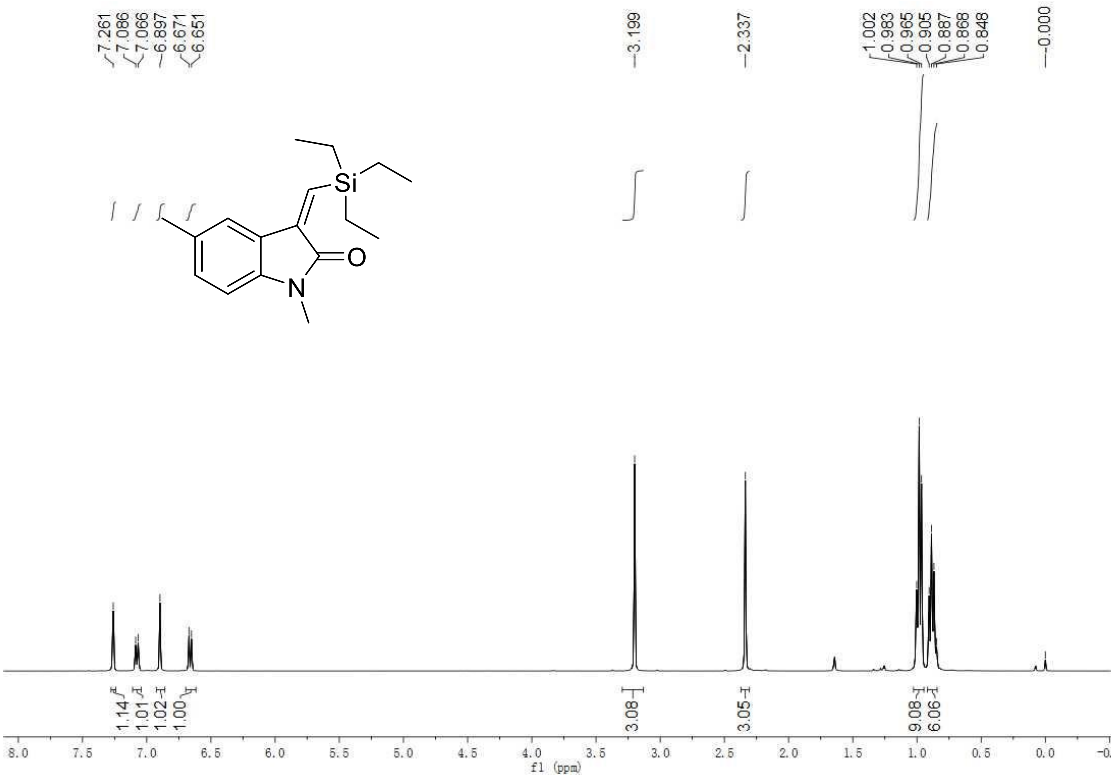

^{1}H-NMR (400 MHz, $CDCl_3$)

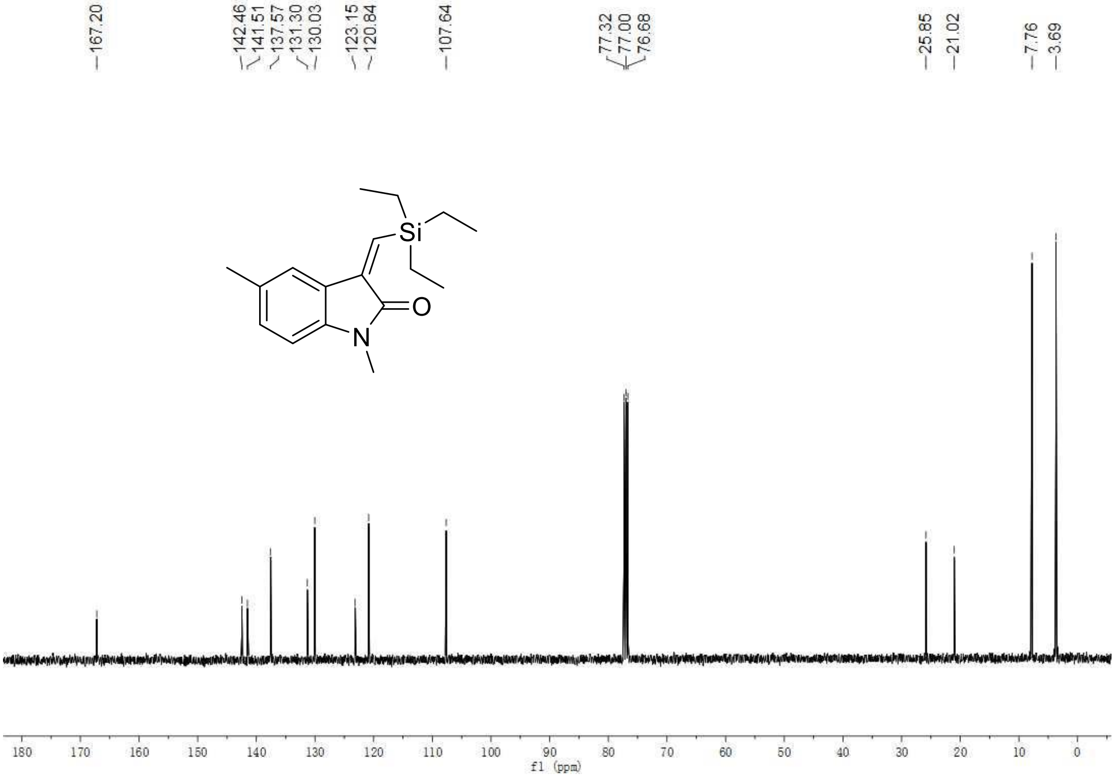

^{13}C-NMR (100 MHz, $CDCl_3$)

(*Z*)-1-methyl-2-oxo-3-((triethylsilyl)methylene)indoline-5-carbonitrile (4na):

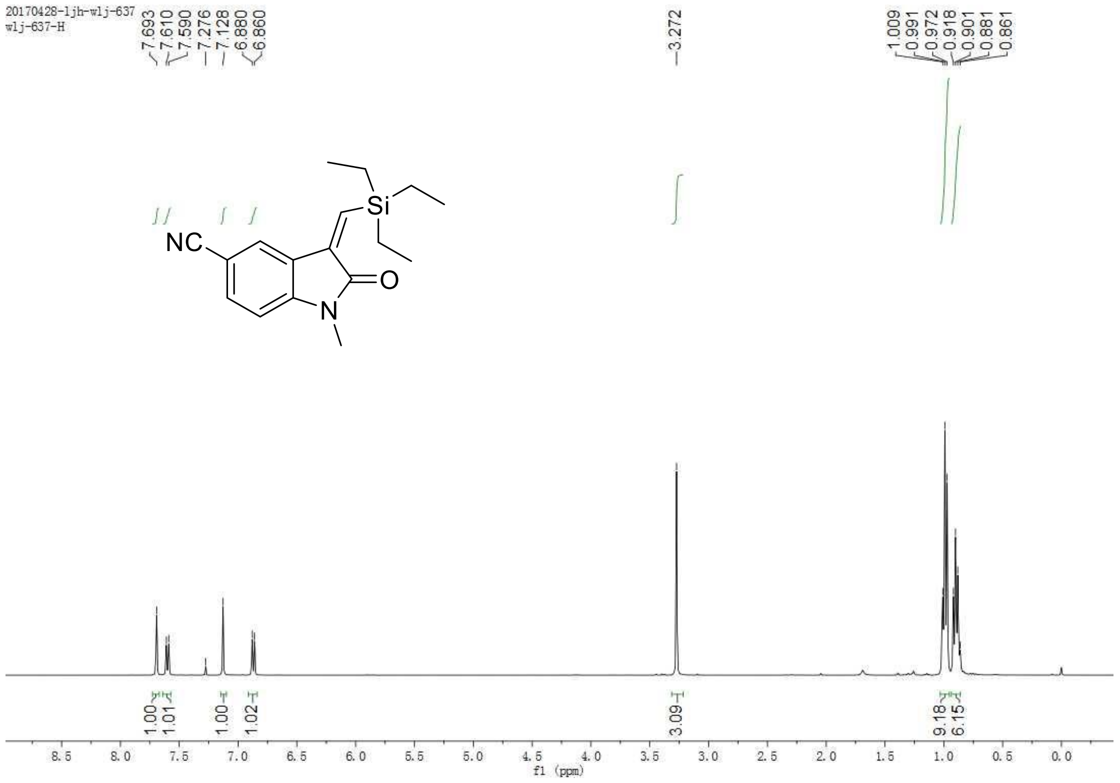

^{1}H-NMR (400 MHz, $CDCl_3$)

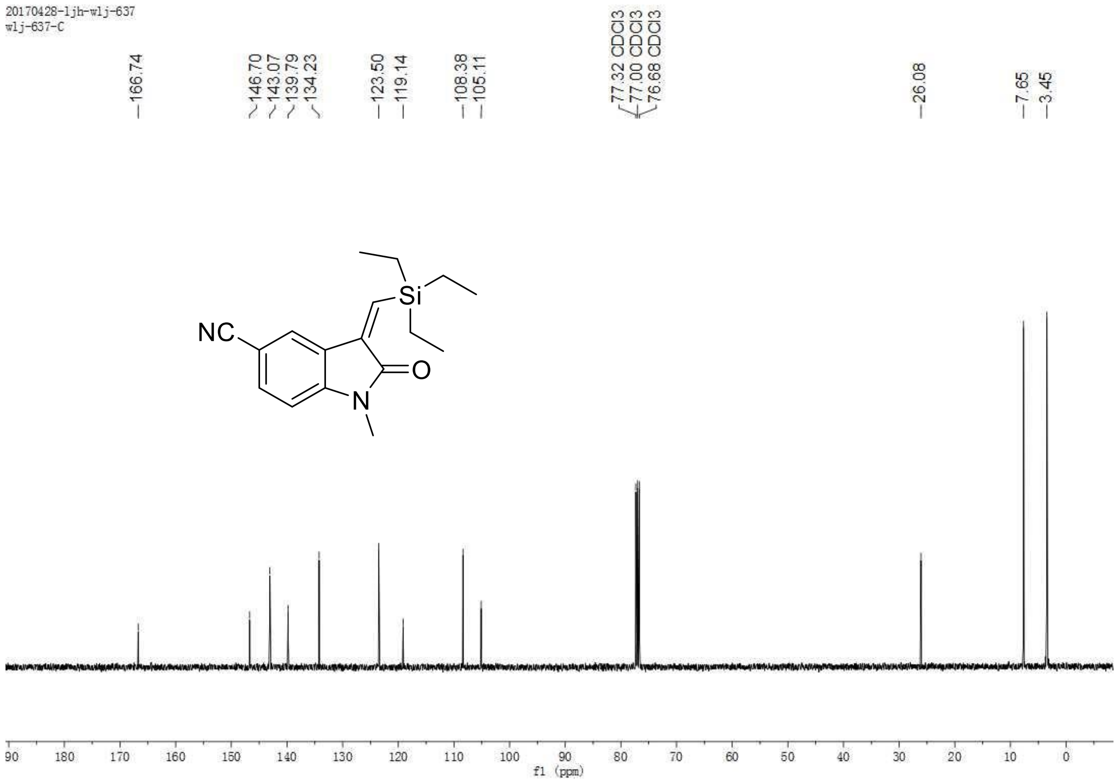

^{13}C-NMR (100 MHz, $CDCl_3$)

(*Z*)-1-methyl-3-((trihexylsilyl)methylene)indolin-2-one (4db):

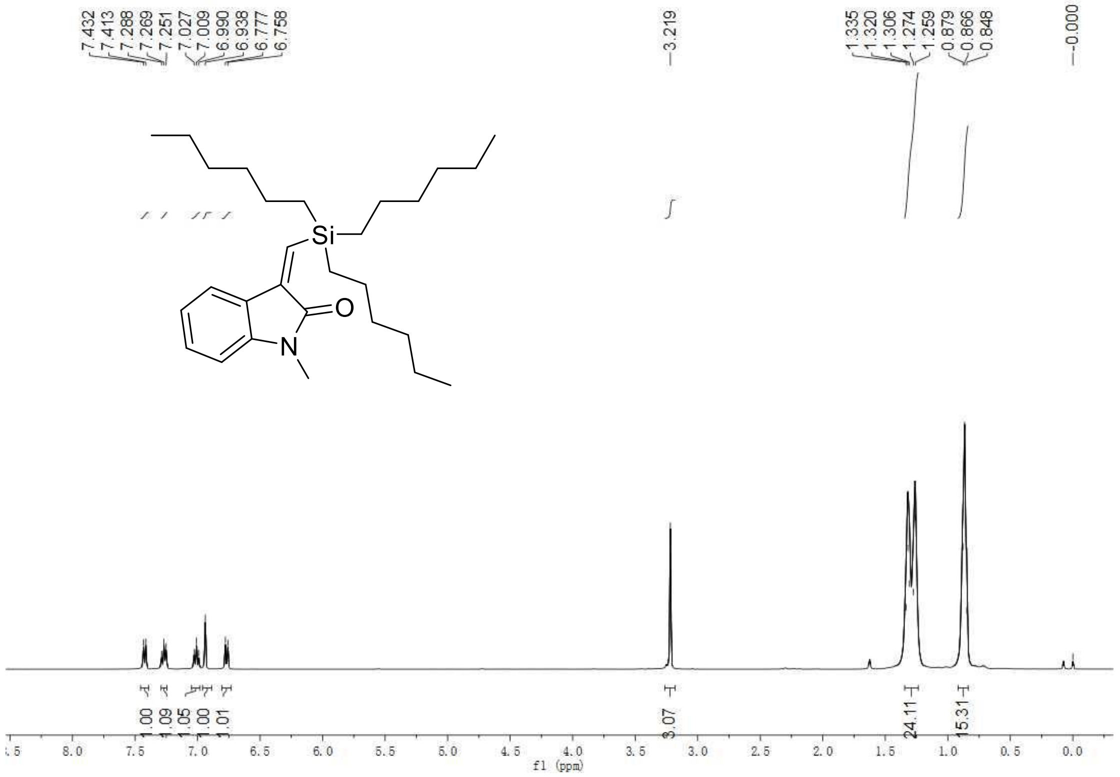

^{1}H-NMR (400 MHz, $CDCl_3$)

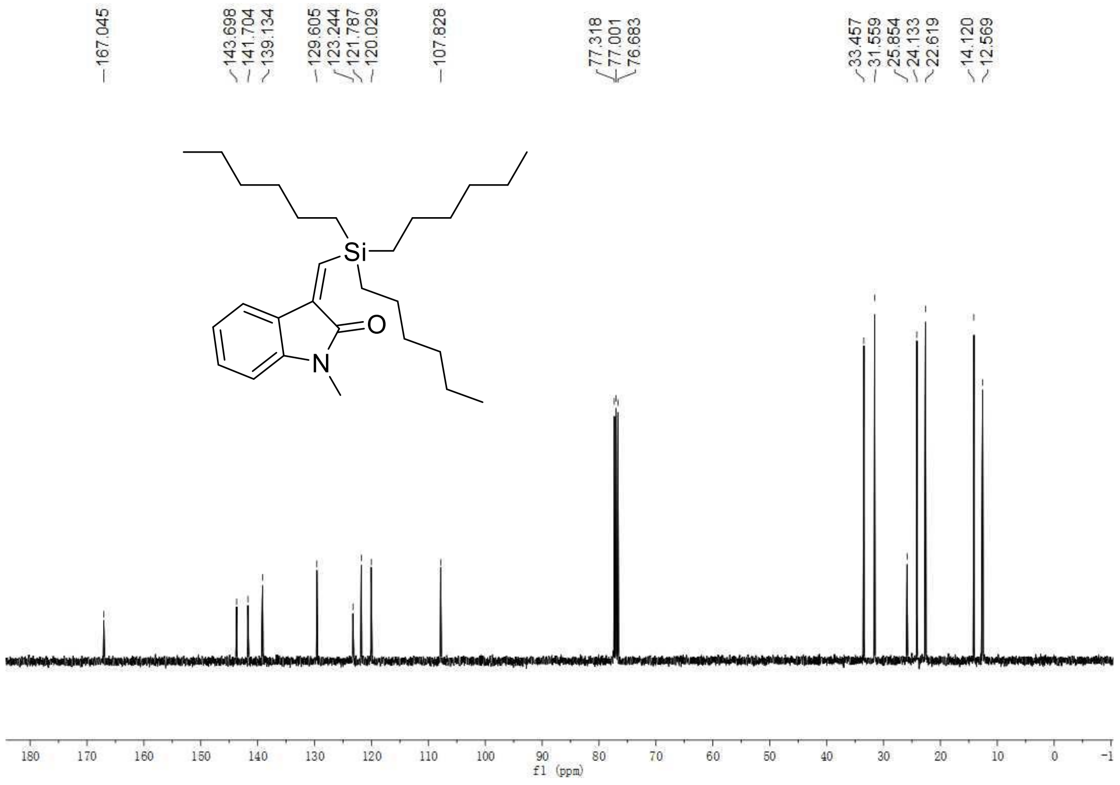

^{13}C-NMR (100 MHz, $CDCl_3$)

(*Z*)-1-methyl-3-((triisopropylsilyl)methylene)indolin-2-one (4dc):

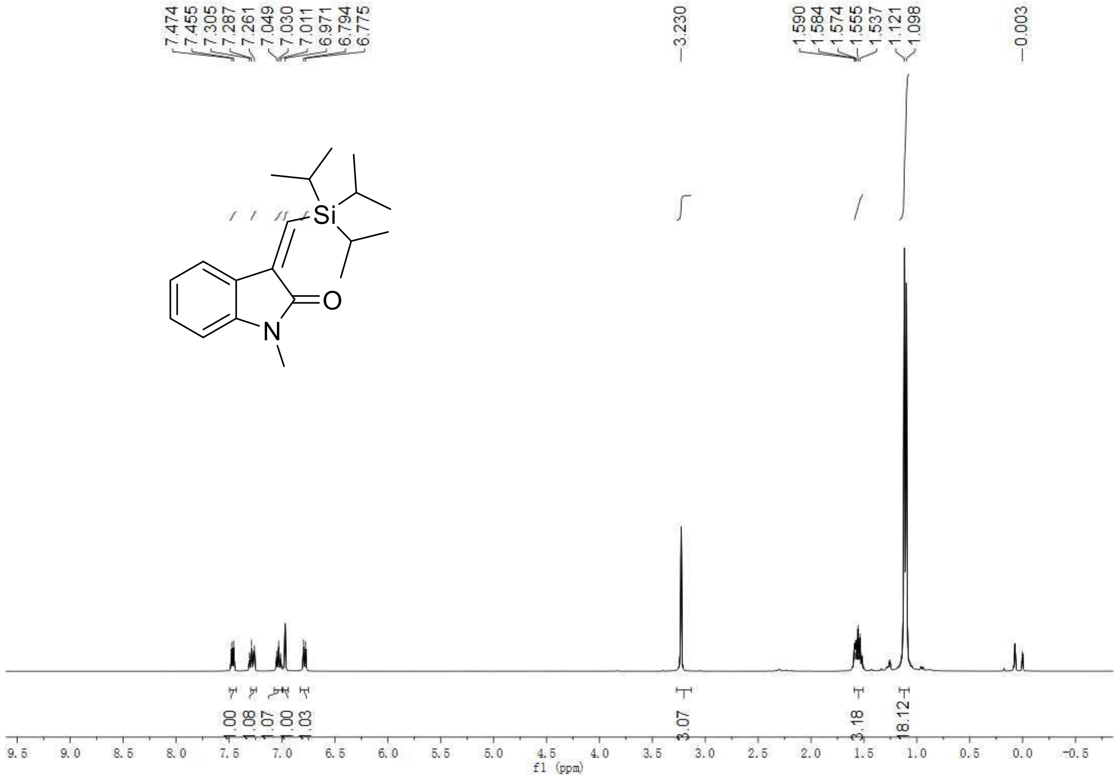

^{1}H-NMR (400 MHz, $CDCl_3$)

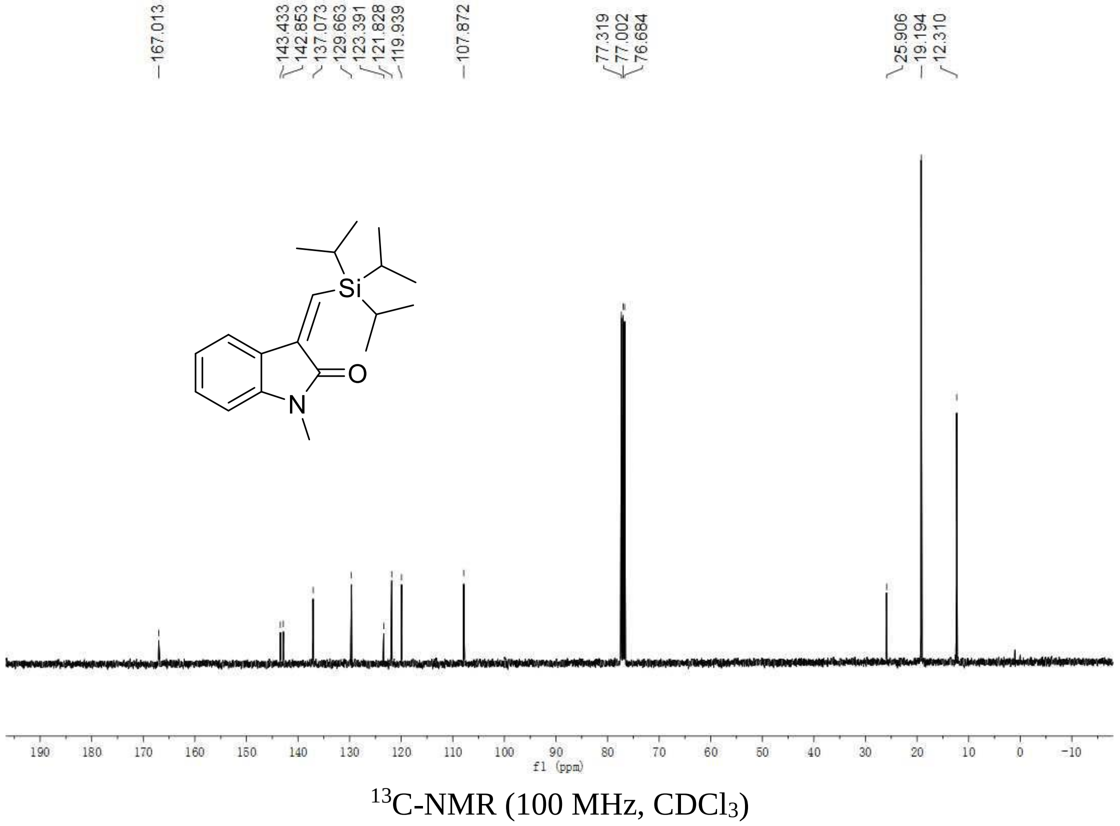

^{13}C-NMR (100 MHz, $CDCl_3$)

(*Z*)-3-((tert-butyldimethylsilyl)methylene)-1-methylindolin-2-one (4dd):

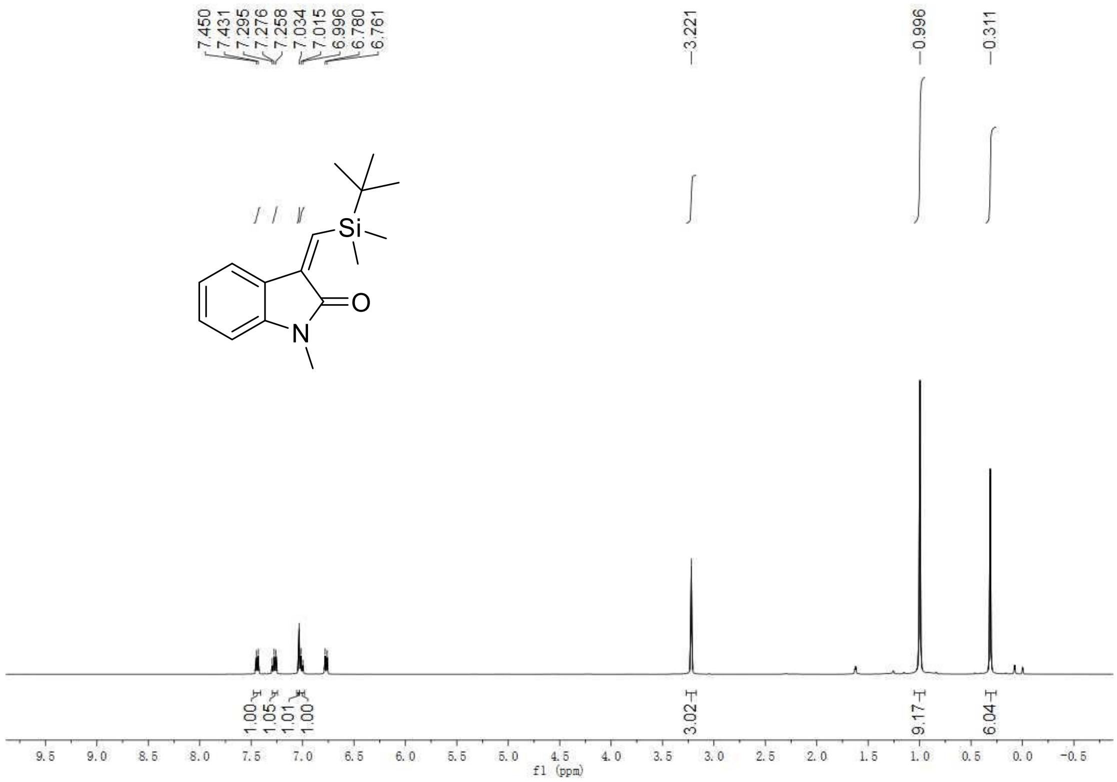

^{1}H-NMR (400 MHz, CDCl$_3$)

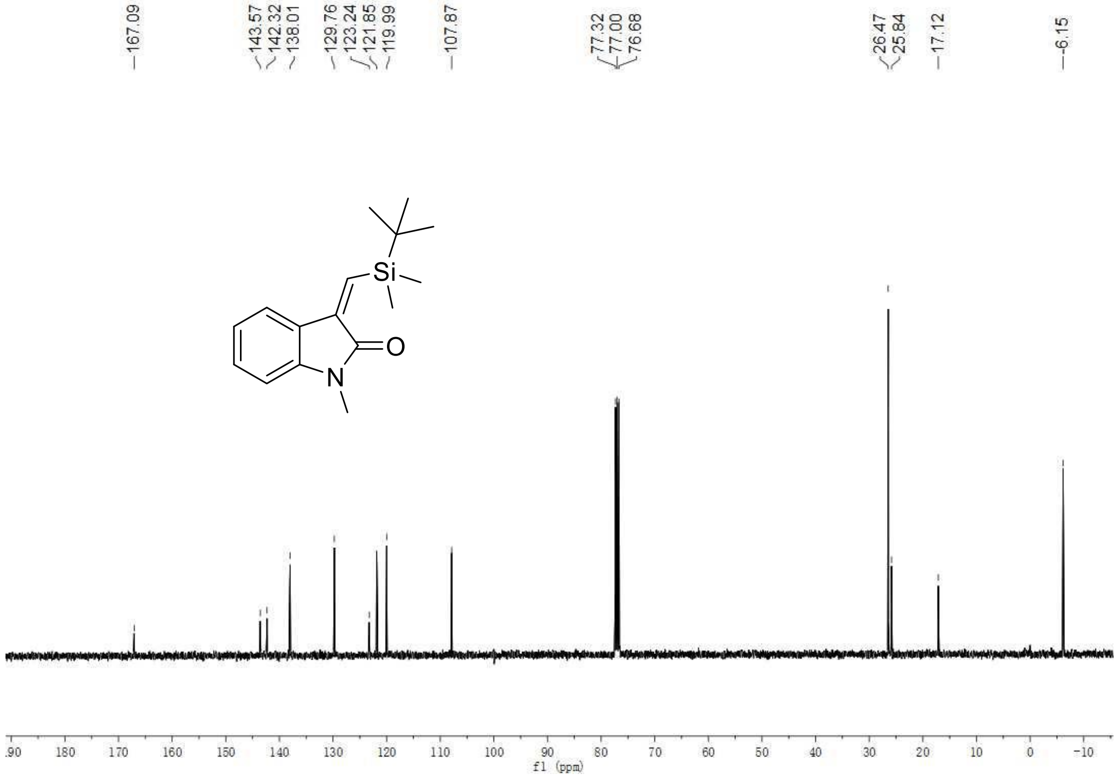

^{13}C-NMR (100 MHz, CDCl$_3$)

(*Z*)-3-((allyldimethylsilyl)methylene)-1-methylindolin-2-one (4de):

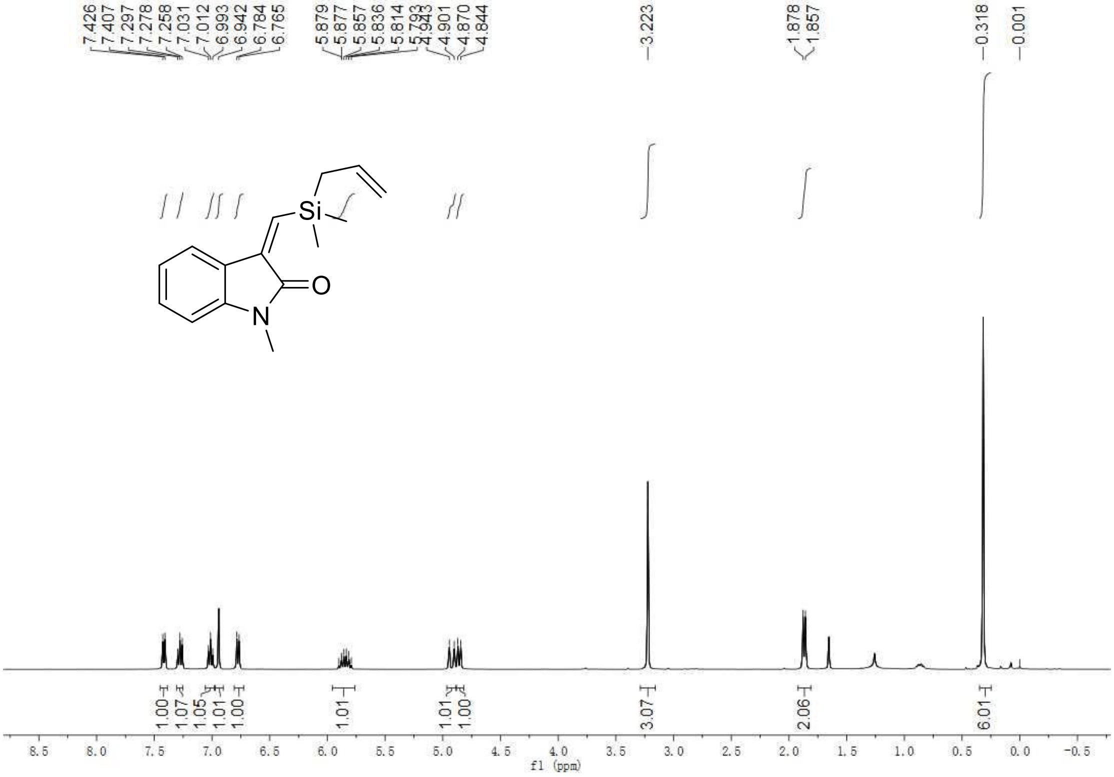

^{1}H-NMR (400 MHz, $CDCl_3$)

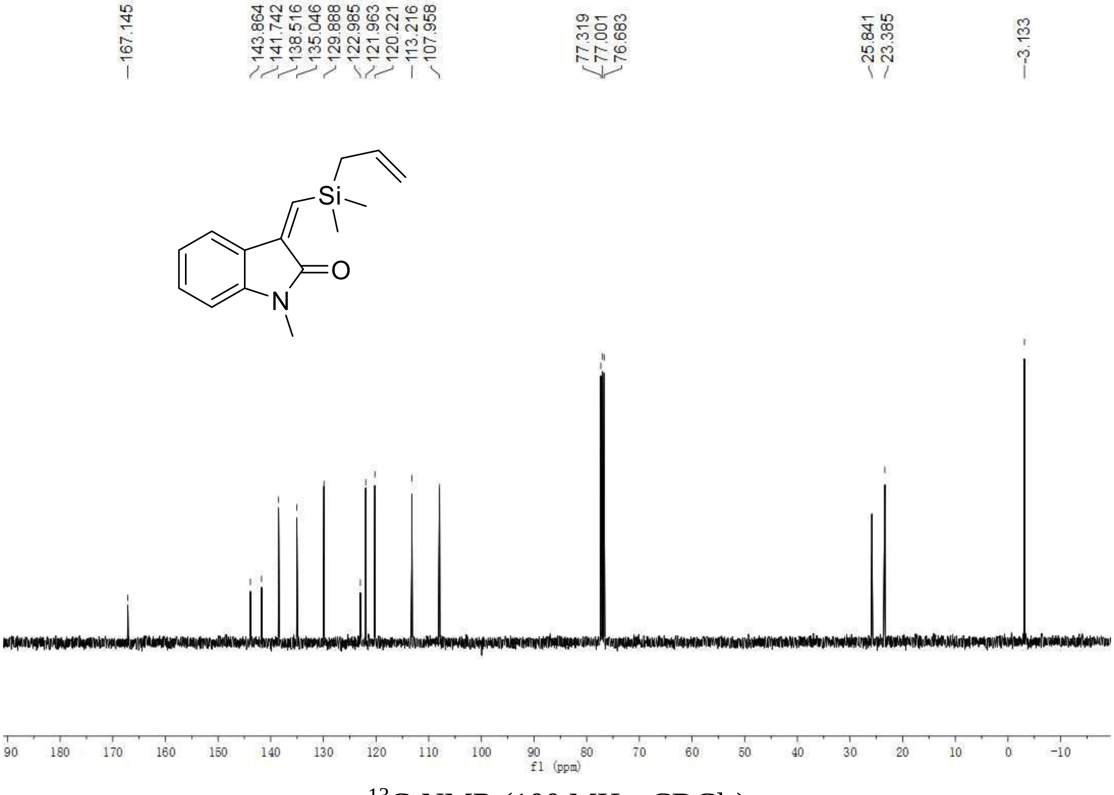

^{13}C-NMR (100 MHz, $CDCl_3$)

(*Z*)-3-((dimethyl(phenyl)silyl)methylene)-1-methylindolin-2-one (4df):

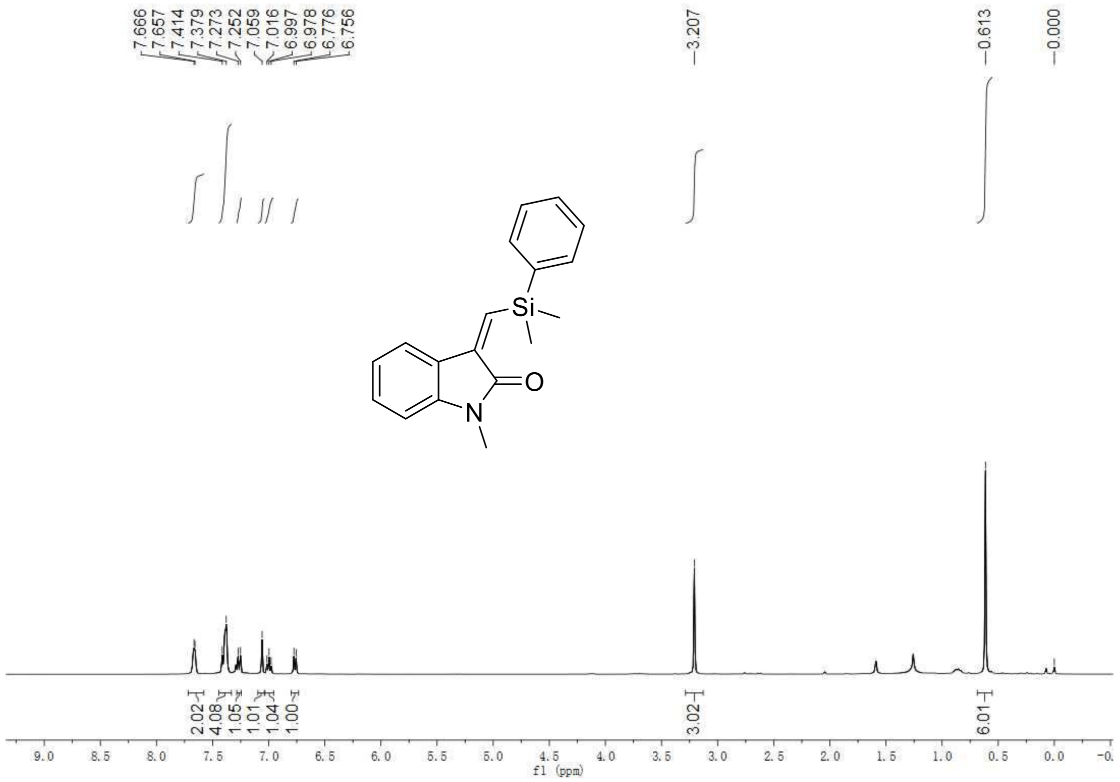

^{1}H-NMR (400 MHz, $CDCl_3$)

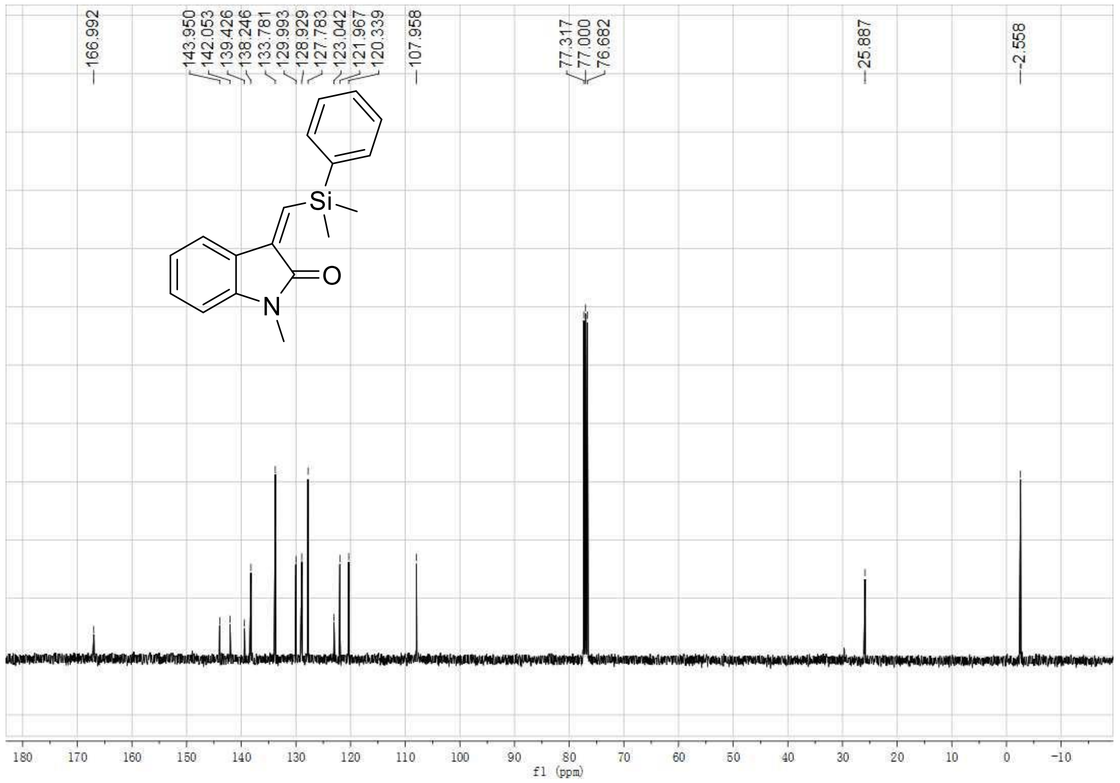

^{13}C-NMR (100 MHz, $CDCl_3$)

(*Z*)-1-methyl-3-((methyldiphenylsilyl)methylene)indolin-2-one (4dg):

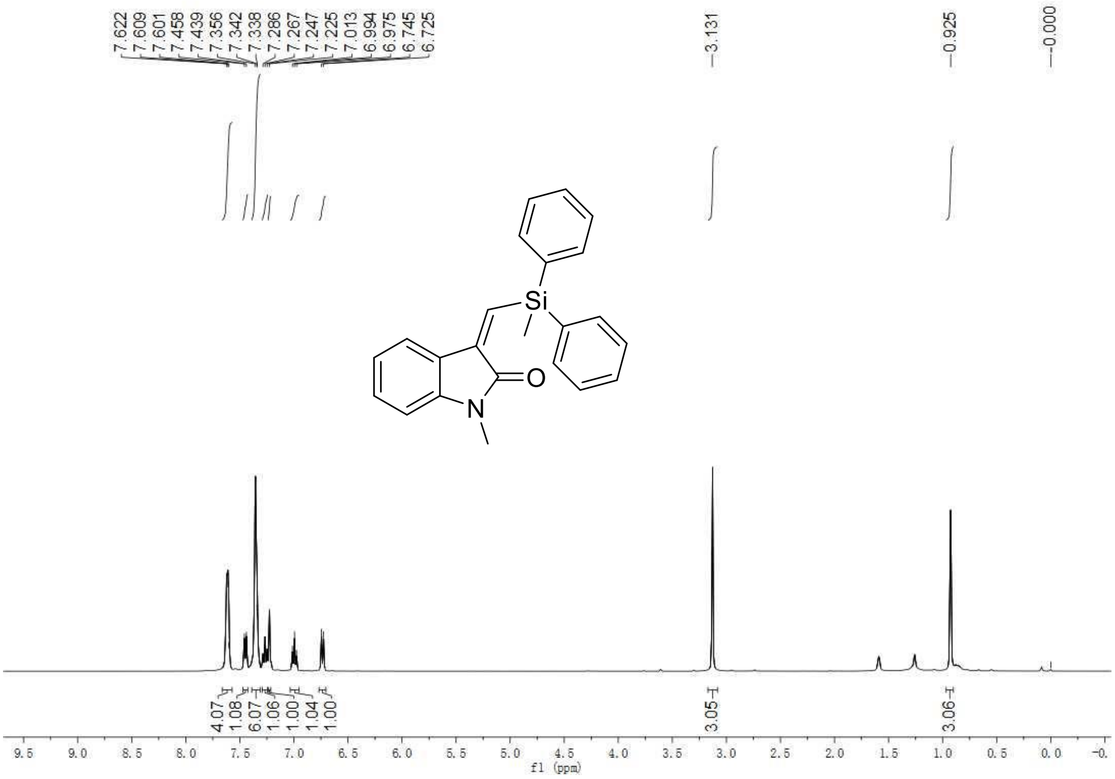

^{1}H-NMR (400 MHz, $CDCl_3$)

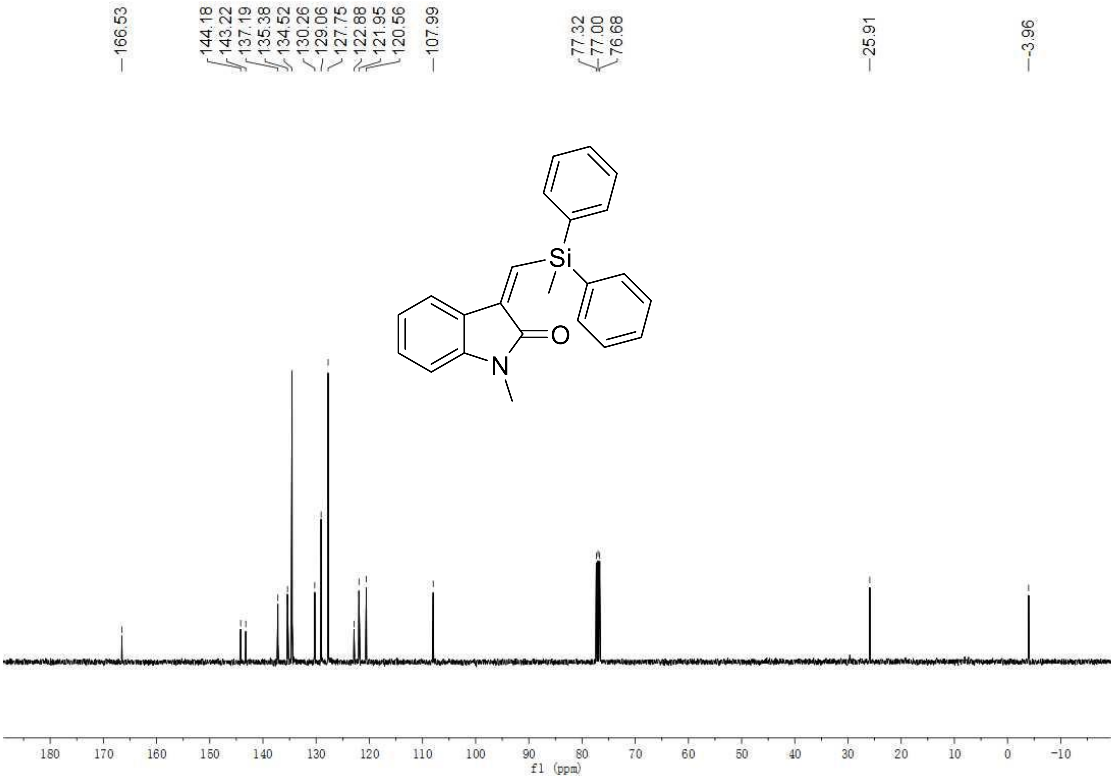

^{13}C-NMR (100 MHz, $CDCl_3$)

(*Z*)-1-methyl-3-((triphenylsilyl)methylene)indolin-2-one (4dh):

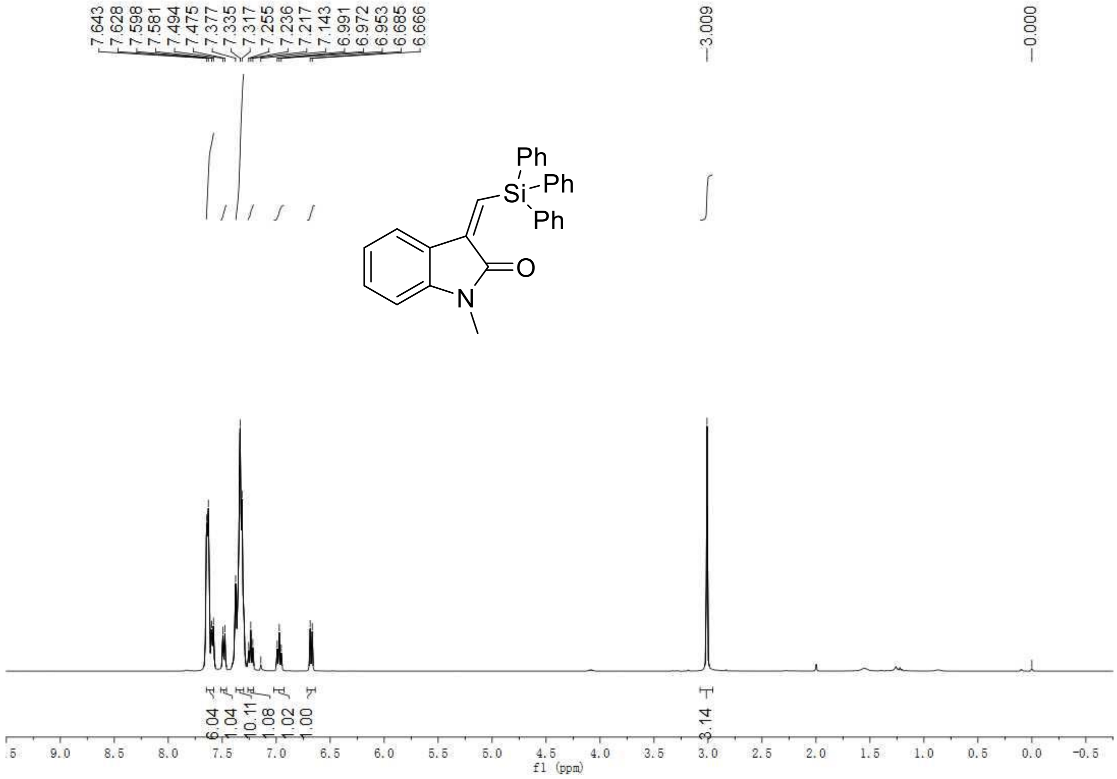

^{1}H-NMR (400 MHz, $CDCl_3$)

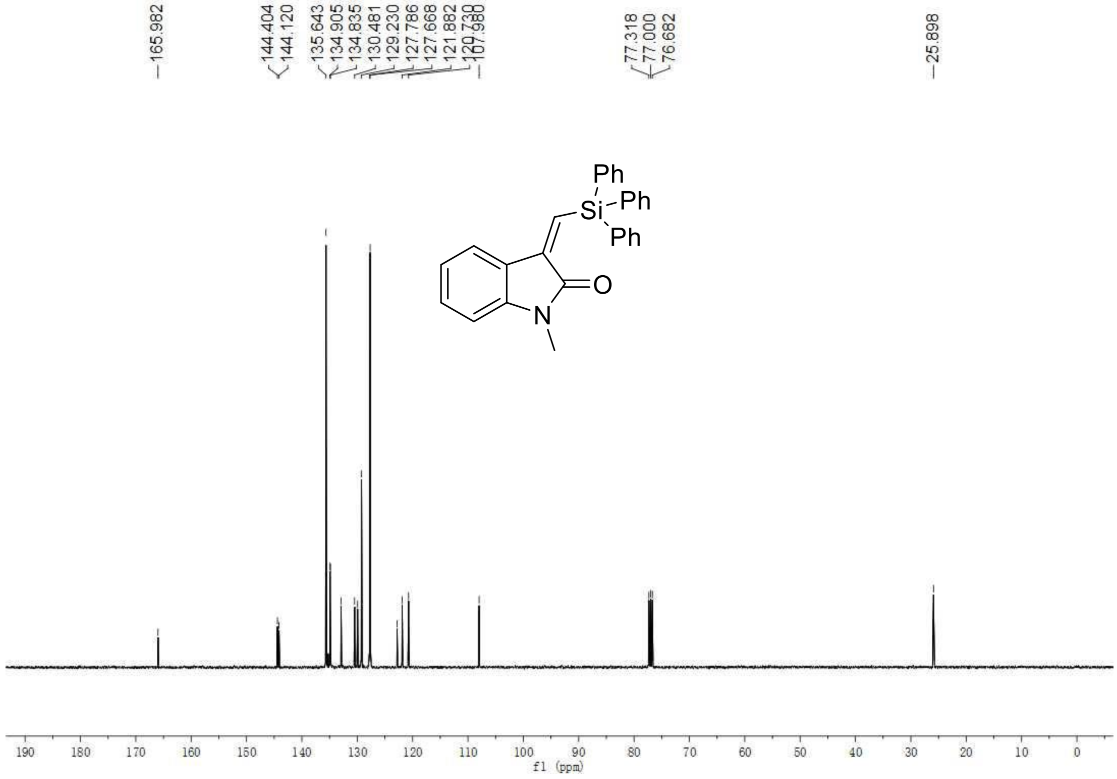

^{13}C-NMR (100 MHz, $CDCl_3$)

N-benzyl-N-(2-((*E*)-3-oxo-1-(triethylsilyl)prop-1-en-2-yl)phenyl)cinnamamide (5eab)

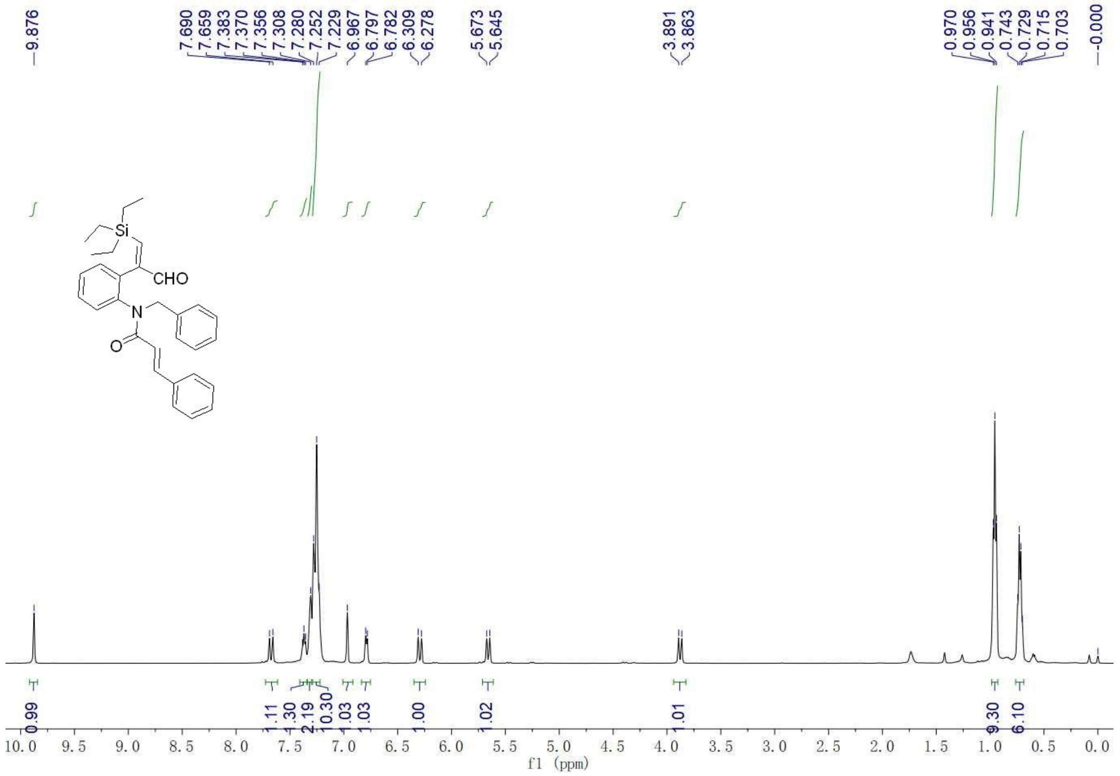

^{1}H-NMR (500 MHz, $CDCl_3$)

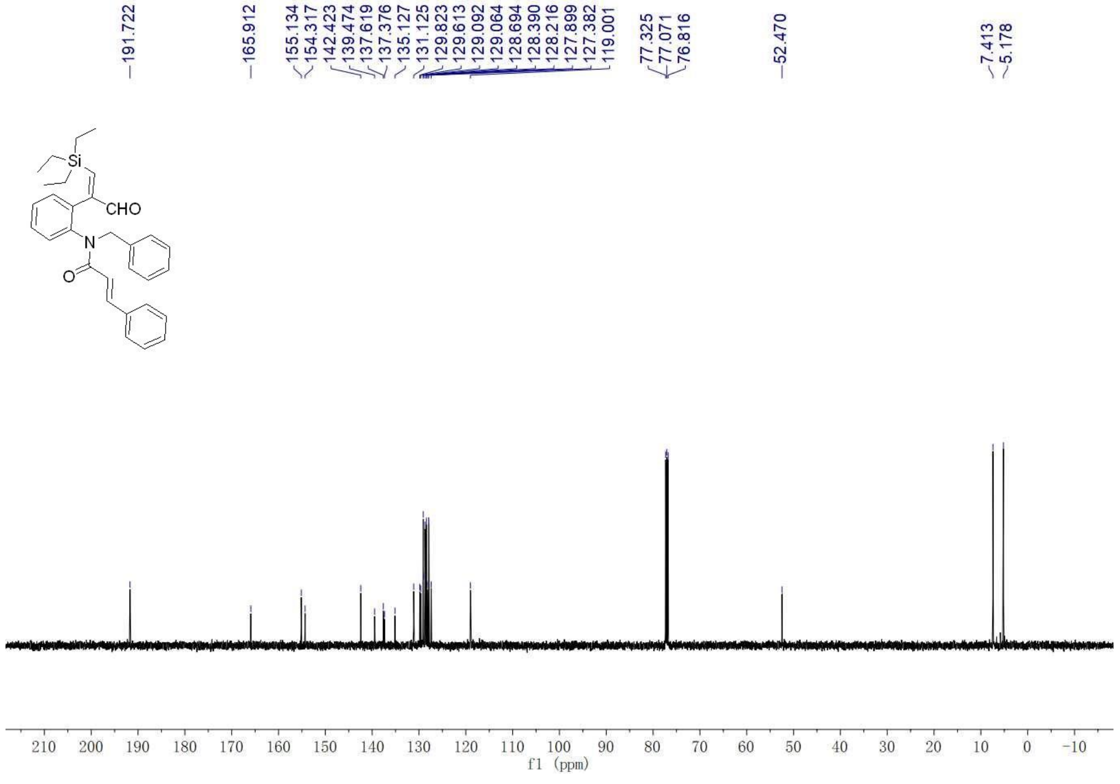

^{13}C-NMR (125 MHz, $CDCl_3$)

(*E*)-N-methyl-N-(2-(3-oxo-1-(triethylsilyl)prop-1-en-2-yl)phenyl)formamide (5dad)

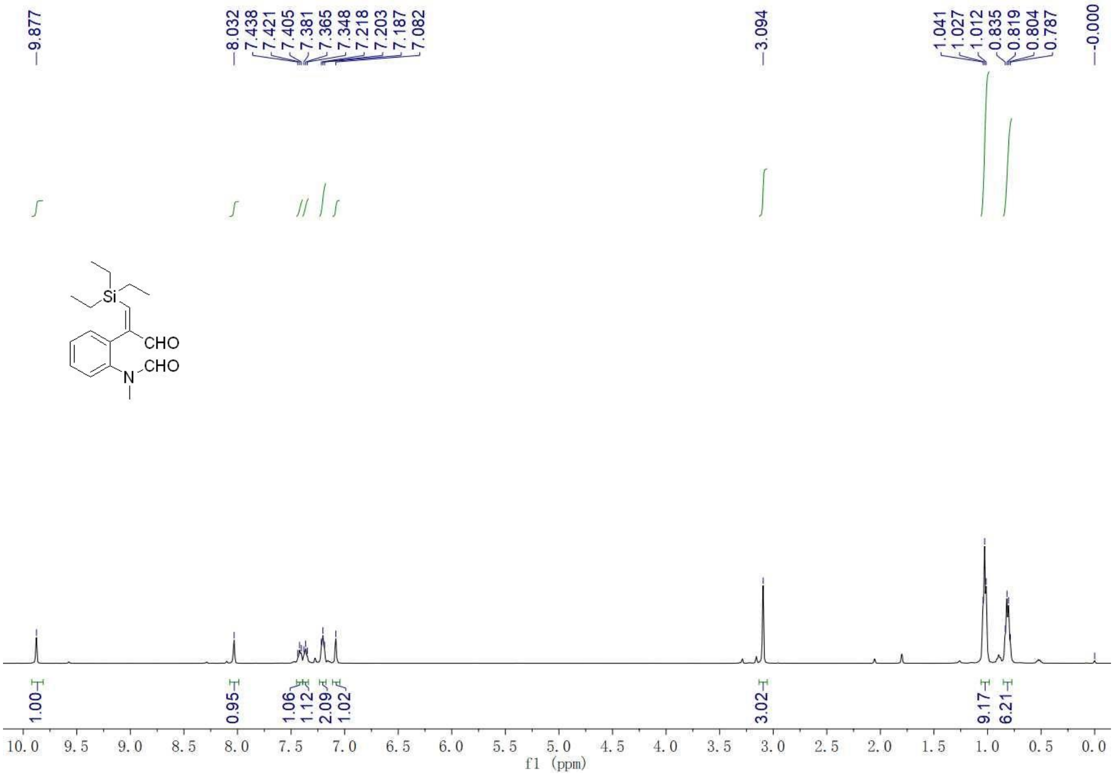

^{1}H-NMR (500 MHz, $CDCl_3$)

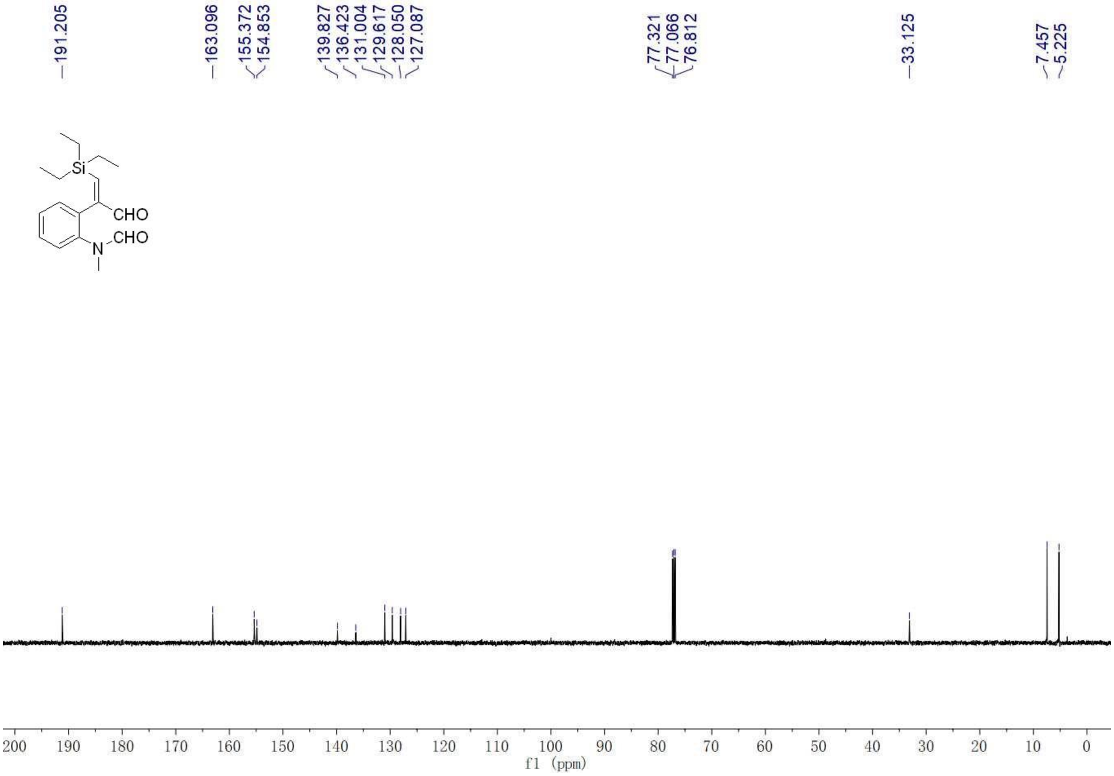

^{13}C-NMR (125 MHz, $CDCl_3$)

(Z)-1-benzyl-3-propylideneindolin-2-one (6ea)

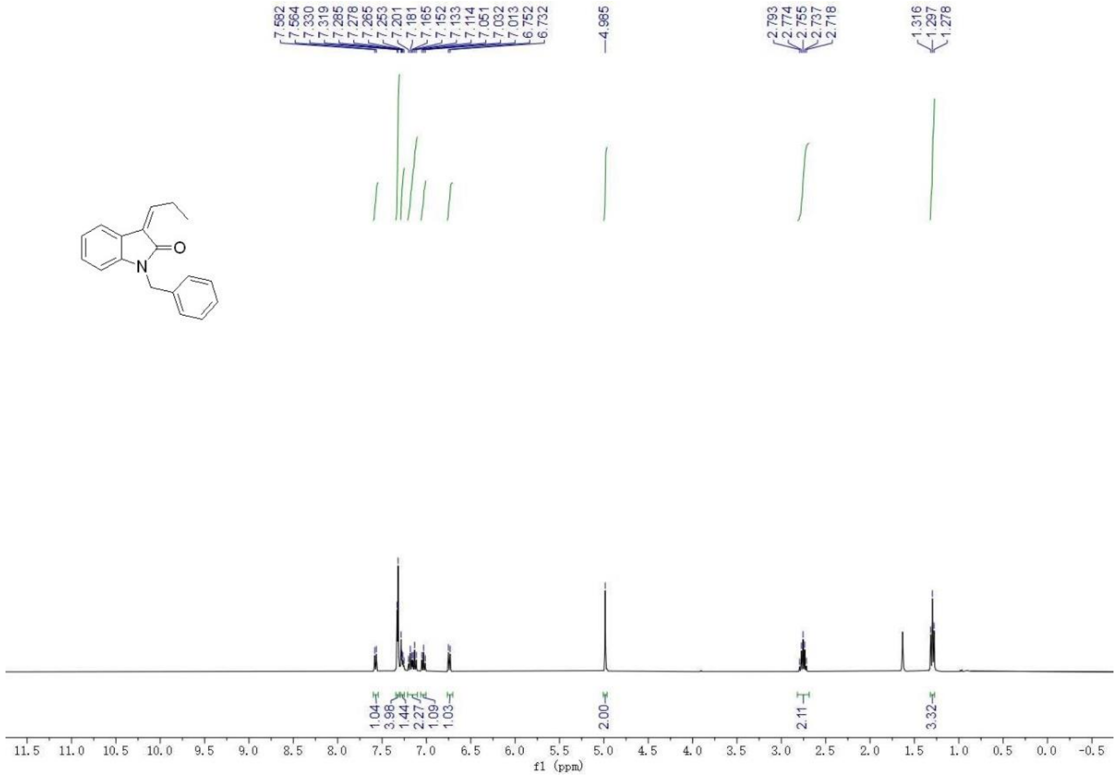

^{1}H-NMR (400 MHz, $CDCl_3$)

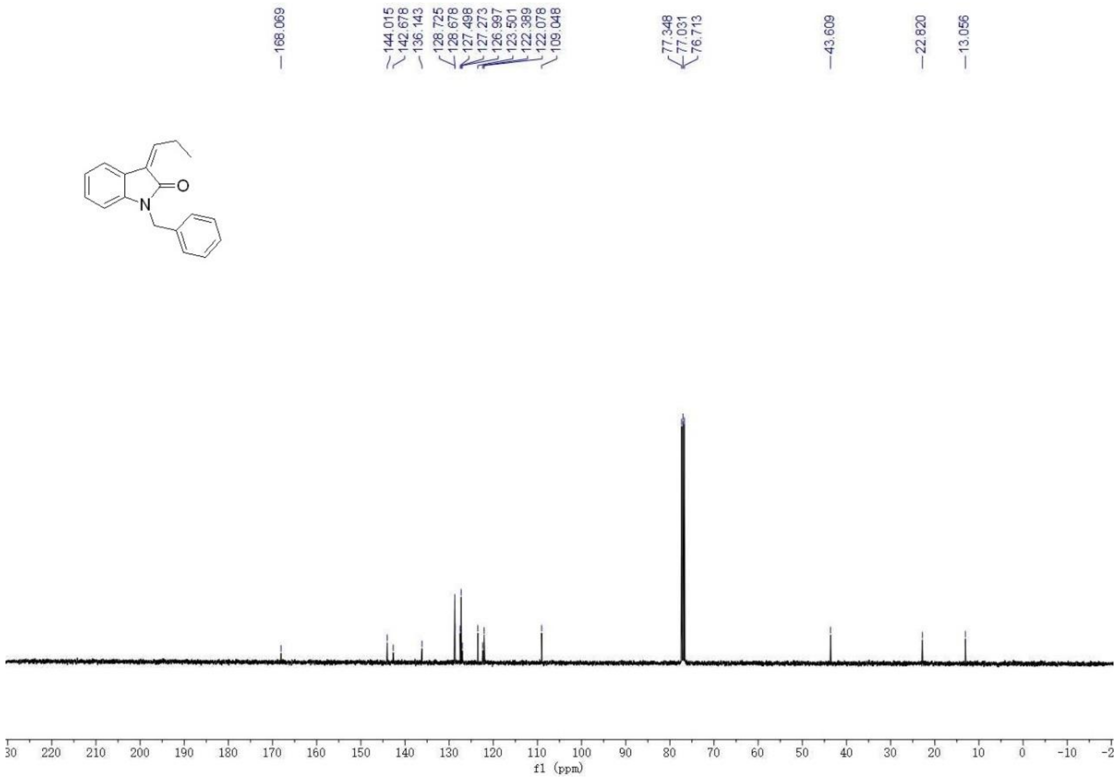

^{13}C-NMR (100 MHz, $CDCl_3$)

1-benzyl-3-hydroxy-3-((triethylsilyl)methyl)indolin-2-one (7ea)

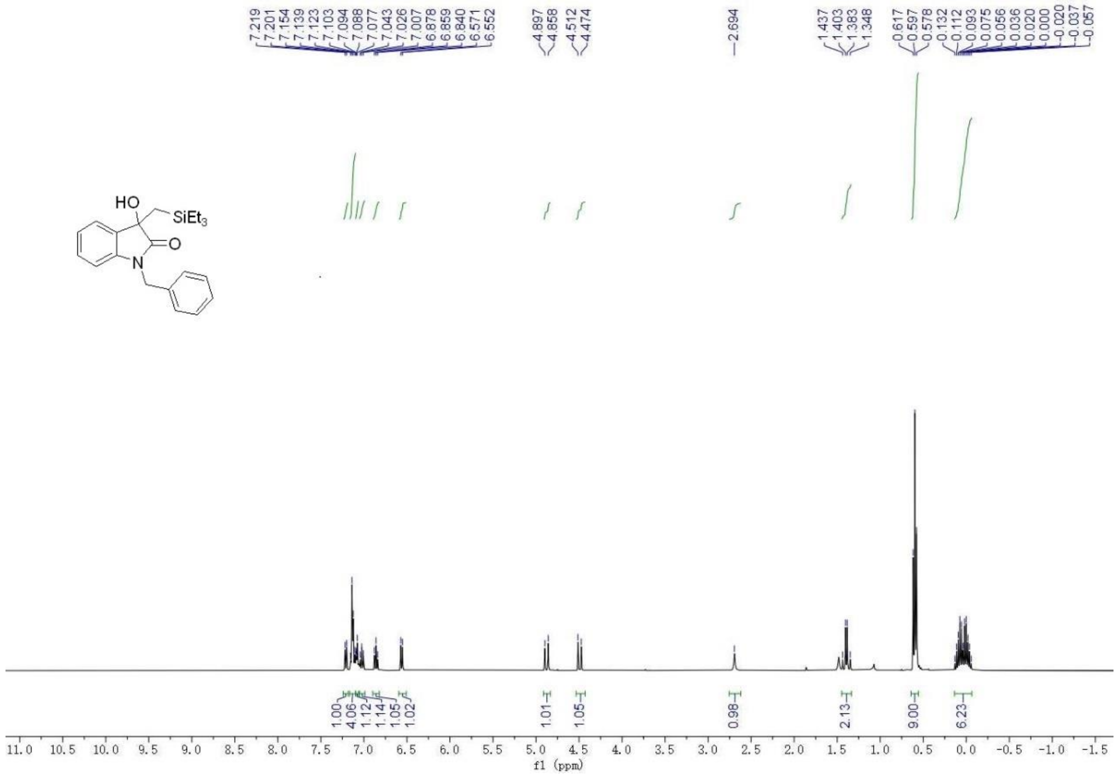

^{1}H-NMR (400 MHz, $CDCl_3$)

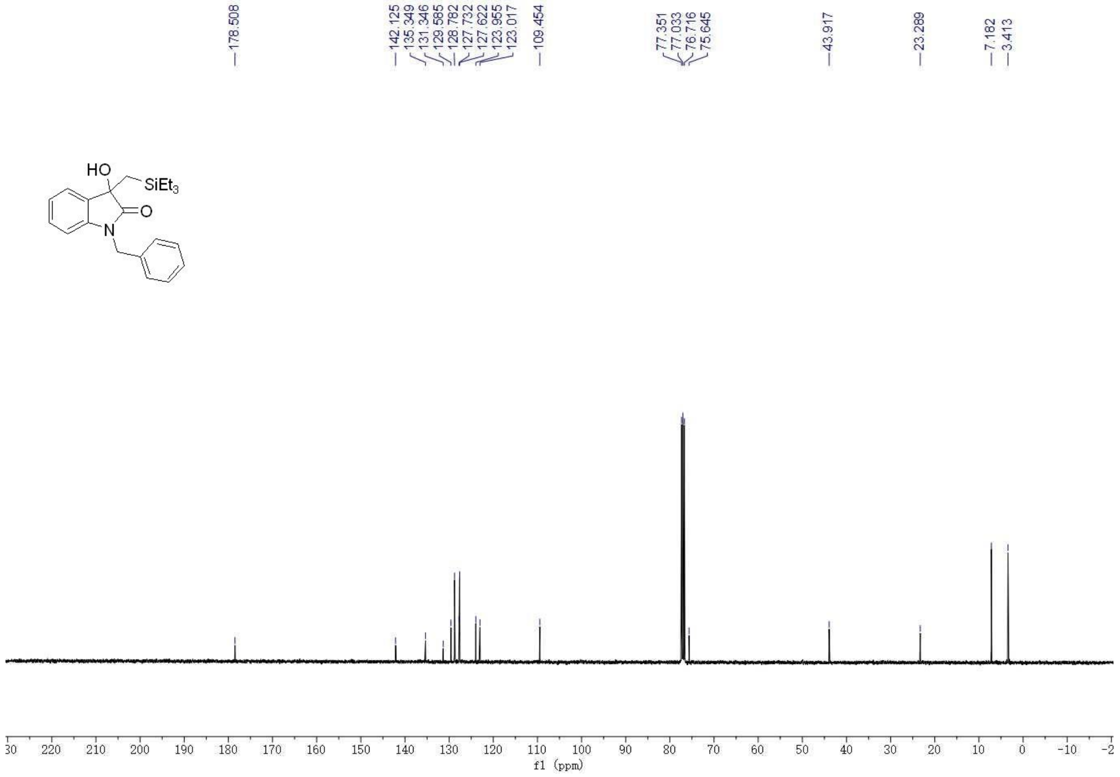

^{13}C-NMR (100 MHz, $CDCl_3$)

(*E*)- and (*Z*)-N-methyl-N-(2-(2-(triethylsilyl)vinyl)phenyl)formamide (8xa)

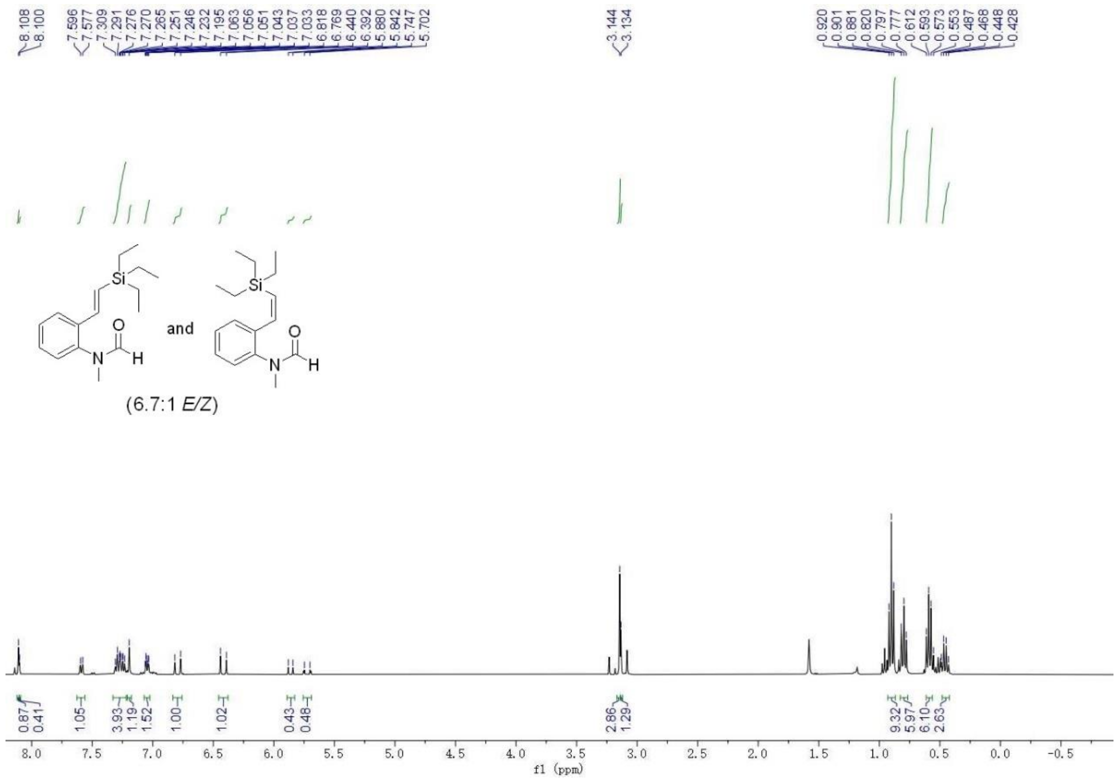

^{1}H-NMR (400 MHz, $CDCl_3$)

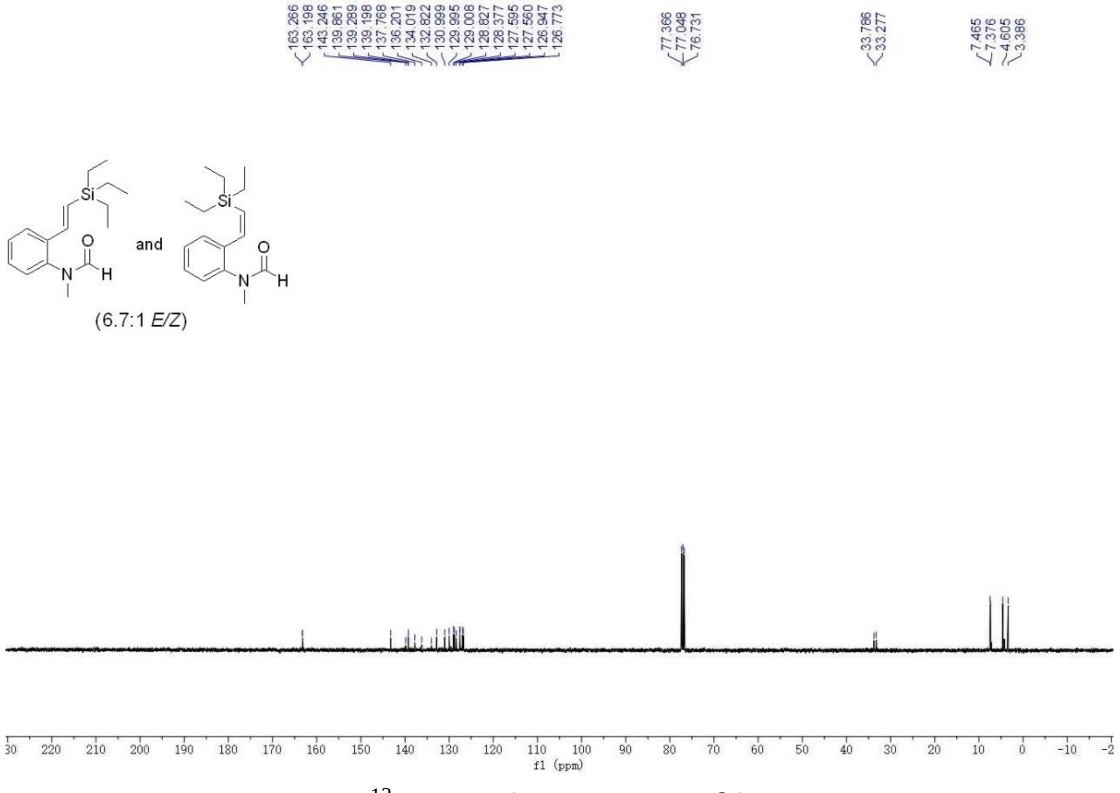

^{13}C-NMR (100 MHz, $CDCl_3$)

(E) References

(1) (*a*) W. Li, Y. Duan, M. Zhang, J. Cheng and C. Zhu, *Chem. Commun.*, 2016, **52**, 7596-7599; (*b*) X.-J. Liu, S. -Y. Zhou, Y. T. Xiao, Q. Sun, X. Lu, Y. Li and J. -H. Li, *Org. Lett.*, 2021, **23**, 7839-7844; (*c*) K. Takamoto, T. Shohei Ohno, S. Ohno, N. Hyogo, H. Fujioka and M. Arisawa, *J. Org. Chem.*, 2017, **82**, 8733-8742.
